# Copy number signatures and *CCNE1* amplification reveal the involvement of replication stress in high-grade endometrial tumors oncogenesis

**DOI:** 10.1007/s13402-024-00942-w

**Published:** 2024-04-02

**Authors:** Regine Marlin, Jean-Samuel Loger, Clarisse Joachim, Coralie Ebring, Guillaume Robert-Siegwald, Sabrina Pennont, Mickaelle Rose, Kevin Raguette, Valerie Suez-Panama, Sylviane Ulric-Gervaise, Sylvie Lusbec, Odile Bera, Alexis Vallard, Aude Aline-Fardin, Emeline Colomba, Mehdi Jean-Laurent

**Affiliations:** 1 Department of Cancer Molecular Genetics, University Hospital of Martinique, Fort-de-France, Martinique; 2 General Cancer Registry of Martinique, University Hospital of Martinique, Fort-de-France, Martinique; 3 Department of Gynecological and Breast Surgery, University Hospital of Martinique, Fort-de-France, Martinique; 4 MitoVasc Unit, SFR ICAT, Mitolab Team, UMR CNRS 6015 INSERM U1083, University of Angers, Angers, France; 5 Martinique Regional Oncology Platform, University Hospital of Martinique, Fort-de-France, Martinique; 6 Biological Resource Center, University Hospital of Martinique, Fort-de-France, Martinique; 7 Department of Oncology Hematology Urology, University Hospital of Martinique, Fort-de-France, Martinique; 8 Cerbapath, Cerba, Martinique; 9 Department of Cancer Medicine, Institut Gustave Roussy, University of Paris Saclay, Gif-sur-Yvette, France

**Keywords:** High-grade endometrial cancer, *CCNE1* amplification, Replication stress, Copy number signature

## Abstract

**Purpose:**

Managing high-grade endometrial cancer in Martinique poses significant challenges. The diversity of copy number alterations in high-grade endometrial tumors, often associated with a *TP53* mutation, is a key factor complicating treatment. Due to the high incidence of high-grade tumors with poor prognosis, our study aimed to characterize the molecular signature of these tumors within a cohort of 25 high-grade endometrial cases.

**Methods:**

We conducted a comprehensive pangenomic analysis to categorize the copy number alterations involved in these tumors. Whole-Exome Sequencing (WES) and Homologous Recombination (HR) analysis were performed. The alterations obtained from the WES were classified into various signatures using the Copy Number Signatures tool available in COSMIC.

**Results:**

We identified several signatures that correlated with tumor stage and disctinct prognoses. These signatures all seem to be linked to replication stress, with *CCNE1* amplification identified as the primary driver of oncogenesis in over 70% of tumors analyzed.

**Conclusion:**

The identification of *CCNE1* amplification, which is currently being explored as a therapeutic target in clinical trials, suggests new treatment strategies for high-grade endometrial cancer. This finding holds particular significance for Martinique, where access to care is challenging.

**Supplementary Information:**

The online version contains supplementary material available at 10.1007/s13402-024-00942-w.

## Background

A higher incidence of aggressive endometrial cancer (EC) has been reported in the Martinique population, with 25% of patients with uterine papillary serous carcinoma (UPSC), 12% of with uterine carcinosarcoma (UCS), and 3% with uterine clearcell carcinoma (UCCC) [1]. These subtypes are associated with a poor prognosis. The care approach for these tumors, often diagnosed at advanced stages, poses a challenge. Martinique, a French Caribbean Island, has a healthcare system like mainland France regarding resources and access. Nevertheless, it faces difficulties accessing care, which may explain why endometrial carcinomas are diagnosed at advanced stages [1]. As suggested by the ESGO/ESTRO/ESP and the National Comprehensive Cancer Network (NCCN) guideline, the primary treatment for EC combines surgery, chemotherapy and radiotherapy [2, 3]. However, this approach is unsuitable for managing high-grade EC, which carries a high risk of metastatic recurrence and mortality. Since 2013, the development of integrated genomic analysis has allowed for the proposal of molecular classification of endometrial tumors [4]. This classification identifies four categories of endometrial carcinomas with distinct clinical, pathologic, and molecular features: POLE (ultra-mutated) (7%) characterized by tumors with mutations in the POLE exonuclease domain [4, 5]; Microsatellite instability (MSI)/hypermutated (28%) by mismatch repair deficiency (MMRd) [4, 6]; serous-like/copy number high (26%) by *TP53* alterations [7, 8]; and copy number low/microsatellite stable (39%) [4]. Compared to the POLE-ultramuted and MSI subtypes, which have a good or intermediate prognosis [4–6], the p53-abnormal subtype has a poor prognosis [7, 8]. Similarly, this subtype does not benefit from therapeutic strategies, unlike the POLE-ultramuted and MSI subtypes, which could benefit from adapted treatment [9–13]. For a better management of patients who have developed a tumor with *TP53* alteration, it is crucial to understand the oncogenesis of this subtype. *TP53*-mutated tumors have been associated with copy number variations (CNVs) [11], which contribute to cancer progression and therapeutic resistance [12–16]. In recent years, the development of several algorithms has made it possible to interpret complex genomic changes by identifying CNVs [17–21]. The classification by signatures of CNVs, based on genome breakpoint number, segment copy number and segment size, reflects etiology and tumor progression. Its use as prognostic or therapeutic resistance markers was soon suggested. In ovarian carcinoma, these signatures has been shown to predict overall survival or the likelihood of resistance to certain therapies [19]. In their study, the authors showed that tumors with a signature characterized by segment amplifications appear to have a worse prognosis than those with a signature characterized by a predominantly diploid segment or by chromosomal rearrangements consisting mostly of Loss of Heterozygosity (LOH). In prostate cancer, tools for studying CNVs also seem promising. Indeed, the involvement of CNVs has been described, with indolent or low-grade tumors characterized by few alterations and primary or metastatic tumors by a greater number of alterations [22, 23]. An association between CNV signatures and pathways underlying oncogenesis has also been described. *CDK12* or homologous recombination gene mutations have been associated with tandem duplication, while *TP53* mutations have been associated with genome duplication. These signatures have also been associated with patient survival suggesting their value in patient management [24]. More recently, a study of all cancers demonstrated that CNV signatures were associated with patient prognosis, and that it was of interest to study their potential for assessing clinical response to certain therapies [21]. On the basis of these observations and with the aim of better understand the oncogenesis of high-grade endometrial tumors with *TP53* mutations, we initiated a molecular study. These objectives are to (i) identify the proportion of *TP53* mutations in a cohort of high-grade endometrial tumors and (ii) assess the signature of copy number alterations associated with *TP53*-mutated tumors. All high-grade endometrial tumors were analyzed using next-generation sequencing (NGS) for a panel of genes implicated in EC. The aim was to select tumors in the serous-like/copy number high category. Analyzing genomic signatures has enabled us to better understand the mechanism of oncogenesis and propose appropriate treatments. For example, PARP inhibitors (PARPi) improve progression-free survival in patients with tumors harboring a genomic scar characteristic of a deficiency in homologous recombination (HR) genes [24]. To better understand the oncogenesis of endometrial tumors with *TP53* mutations, we performed a Whole-Exome Sequencing (WES) to generate several alterations signatures of tumors and a Homologous Recombination (HR) analysis.

## Methods

### Patient selection

All patients presenting with high-grade endometrial cancer and referred to the University Hospital of Martinique were included in the study, which has received approval from the ‘Committee for the Protection of Individuals Sud Mediterranee IV’ (ID-RCB: 2018-A209154). Patients were enrolled over 18 months from November 2019 to March 2021 and were followed for 2 to 36 months with a median month of 22. Twenty five patients, representing all high-grade endometrial tumors diagnosed during this period, were included. The histological classification was made according to the recommendations of the World Health Organization [25] by a dedicated pathologist. Patients were included at the time of endometrial cancer diagnosis, which was determined either from a biopsy or curettage. They underwent an initial evaluation using a computed tomography scan and bone scintigraphy. Detailed clinical information related to the EC diagnosis was gathered, including age at diagnosis, body mass index (BMI), parity, menopausal status, comorbidities (such as arterial hypertension, diabetes, and others), treatment received, histological type, stage at diagnosis (TNM), and FIGO classification. Each patient provided written informed consent for genetic analysis of their tumor. Tumor DNA was extracted from a specimen prior to surgery to determine the initial mutation landscape using a panel of genes associated with EC (Supplemental Table 1). The primary treatment for patients without distant metastases typically involved a hysterectomy with bilateral salpingo-oophorectomy, followed by chemoradiotherapy (CRT). Depending on disease progression, some patients received only chemotherapy (CT) or radiotherapy (RT). For those with metastases, neoadjuvant treatment was administered.

### p53 immunohistochemistry (IHC)

For each case, a pathologist selected one representative formalin-fixed paraffin-embedded (FFPE) tumor block. p53 IHC was performed on the Ventana Benchmark autostaining system using a mouse monoclonal antibody (DO-7) at 0.5 µg/ml after antigen retrieval in CC1-COURT buffer followed by detection with the ULTRAVIEW-DAB system (Roche/Ventana Medical Systems). Cases were categorized into one of three groups. Wildtype p53 expression was defined as nuclear staining of variable intensity in 1–80% of the tumor. Null expression was characterized by the absence of p53 nuclear staining with a positive internal control, while overexpression was identified by uniform and intense nuclear staining in at least 80% of tumor cell nuclei. It’s important to note that, for the purposes of this study, ‘ambiguous’ p53 expression was not considered a category, ensuring that all cases were assigned one of the three p53 IHC patterns.

### Molecular analysis

#### Genes panel sequencing and selection of TP53 pathogenic mutations

Twenty-three patients underwent NGS analysis using a primary genes panel sequencing approach. For each patient, an endometrial curettage in abnormal uterine was carried out. Peripheral blood samples were collected. Human samples were obtained from the processing of biological samples by the biological resource center of Martinique. We ensured the variants were not germline mutations using comparative analysis ‘tumor-germline’ for sequencing analysis. Germline and tumor DNA were extracted using the Maxwell RSC platform (Promega, France). At first, according to the classification of EC, we conducted microsatellite instability (MSI) and tumor panel sequencing, which include genes incriminated in EC (Supplemental Table 1). The pipeline bioinformatic included the bcl2fastq tool for demultiplexing and generation of fastq files. Reads mapping to the human reference genome (GRCh38) was performed using BWA [26]; recalibration was performed using GATK software (Broad Institute) and variant calling using Varscan2 v.2.3.9 [27] and SomaticSniper v.1.0.5.0 [28]. Variants detected by this pipeline were annotated by SnpEff v.4.3 [29]. For each sequencing run, quality reports integrating the number of clusters/mm2, percentage of bases with a Qscore > 30, FastQC reports, percentage of mapped reads, on- and off-targets percentages, percentage of covered bases, and mean sequencing depth were generated using Samtools [30], bedtools [31] and Picard softwares. Only nonsense, frameshift, and missense variants described as pathogenic according to ClinVar were reported. We also used the framework VIPUR database [32] to predict the effects of missense mutations on the p53 protein and IARC TP53 Database to model the structure of missense mutant DNA-binding domain. Recently, VIPUR was used to model the structure of p53 missense protein mutants. High VIPUR scores (0.8-1.0) are associated with poorly folded or non-functional proteins, while the lowest VIPUR scores (0.1–0.5) are closer to the native structure of the proteins.

### Signatures of copy number alterations

The analysis of the rearrangement alteration was done through Whole-Exome Sequencing (WES). These examinations were conducted on tumors harboring *TP53* mutations. WES was performed on frozen tumors from a total of 17 patients. The library preparation was performed by the “DNA Prep with Exome 2.0 Plus Enrichment S Tagmentation” (Illumina). Libraries were sequenced on a NextSeq 1000 platform. The quality of raw sequencing data was assessed using FastQC v0.11.9 and MultiQC v1.13 [33]. The fastq files were subsequently mapped onto the reference human genome (GRCh38/hg38) using the Burrows-Wheeler Aligner’s maximal exact matches (MEM) algorithm in BWA v0.7.17 [34]. Post alignment, duplicates were identified and removed using the ‘markdup’ functionality within SAMtools v1.15.1 [30]. Copy Number Variations (CNVs) were detected using the Fraction and Allele-Specific Copy Number Estimates from Tumor Sequencing (FACETS) package v0.5.14 [35] within R v3.6.2. The wrapper tool CNV_facets v0.16.0 was used to execute FACETS analysis. FACETS requires a reference SNP panel; for this purpose, we used dbSNP (file accessed from https://ftp.ncbi.nlm.nih.gov/snp/organisms/human_9606/VCF/00-common_all.vcf.gz) [36]. We used the -T parameter in cnv_facets, employing the hg38_Twist_ILMN_Exome_2.0_Plus_Panel_annotated.BED file, to specify exomic target regions. Somatic and germline variant calling were performed using Strelka v2.9.10 [37] with default parameters. Annotations were subsequently made using Variant Effect Predictor (VEP) v109.3 [38]. We constructed profiles of the quantity of Copy Number Variations (CNVs) utilizing the Fraction and Allele-Specific Copy Number Estimates from Tumor Sequencing (FACETS) tool [35]. Subsequently, the CNVs were classified according to the framework outlined by Steele to generate Copy Number Signatures [20]. We generated the signatures utilizing SigProfiler Bioinformatic tools provided by COSMIC. Alterations are categorized based on three parameters: the total copy number of the segment (TCN) (TCN0: no copy of DNA segment; TCN1: one DNA segment copy; TCN2: two DNA segment copies; TCN3-4: minor gain DNA segment copies; TCN5-8: moderate gain DNA segment copies; TCN ≥ 9: high-level amplification DNA segment copies), the heterozygosity status, and the segment size (Segment size classes: 0 to 100 kb; 100 kb to 1 Mb; 1 Mb to 10 Mb; 10 Mb to 40 Mb; >40 Mb). By applying these parameters to TCGA tumors, a total of 21 pan-cancer signatures of copy number variations have been generated (see Supplemental Table 2). We selected this tool due to its optimization for WGS, WES, and SNP6-profiling platforms and its utilization with data from the Cancer Genome Atlas (TCGA) [20]. Additionally, the tools available in COSMIC enabled us to generate copy number signatures rapidly. We successfully extracted signatures from our own tumor genome sequencing data, which were then compared with the 21 existing set of reference signatures [20] (https://cancer.sanger.ac.uk/signatures/cn/). Supplemental Fig. 1 illustrates the Copy Number Signatures generated for the 17 tumors.

### Single-base substitution (SBS), double-base substitution (DBS) and insertion/deletion (ID) signatures

All signatures are generated using SigProfiler Bioinformatic tools provided by COSMIC. Mutational spectra were obtained from Mutect2 [39], which generates VCF files. The variants were classified according to the framework described by Alexandrov [40]. We extracted single-base substitution (SBS), double-base substitution (DBS) and insertion/deletion (ID) signatures from our own tumor exome sequencing data, which were then compared with the set of reference signatures available in COSMIC [40]. For SBS, there are a total of six possible classes of base substitutions at each variant site: C > A, C > G, C > T, T > A, T > C, and T > G (for each base pair, the mutated base is represented by the pyrimidine). By considering the bases immediately 5′ and 3′ to each mutated base, there are a total of 96 possible mutation classes, referred to as triplets, in this classification [40] (https://cancer.sanger.ac.uk/signatures/sbs/). For DSB signature, there are 78 strand-agnostic DBS mutation types available in Cosmic (https://cancer.sanger.ac.uk/signatures/dbs/). ID signature corresponds to the classification of small insertions or deletions de fragments between 1 and 50 base pairs in a specific genomic location. According to the framework of Alexandrov, a compilation of 83 different types considering size, nucleotides affected and presence on repetitive and/or microhomology regions was used to extract 23 mutational signatures (https://cancer.sanger.ac.uk/signatures/id/).

### Tumor mutational burden (TMB)

We employed the SigProfilerAssignment tool to analyze the somatic mutation signatures present in our samples. This involved a precise computational subtraction process, where germline variants were subtracted from somatic tissue variations, effectively isolating pure somatic mutations. To quantify the tumor mutational burden (TMB), we counted the number of non-synonymous mutations and normalized this count by dividing it by the total number of bases in our target region, as defined by our BED file. This approach provided us with a nuanced measure of the mutation rate across the tumor exome. Furthermore, to ensure the accuracy and reliability of our TMB calculations, we utilized the TMBler tool as a validation mechanism. This rigorous methodology allowed for a robust analysis of mutational landscapes, offering valuable insights into the genomic alterations characteristic of our samples.

### Homologous recombination repair (HRD) analysis

For HR analysis of EC with a *TP53* mutation, we utilized the commercial SOPHiA DDM™ Homologous Recombination Deficiency (HRD) Solution from SOPHiA GENETICS™. Due to insufficient material for one tumor, 16 tumors were analyzed. Sophia Solution combines analysis of genomic instability (Genomic Integrity Index or GII) with the mutational status of 28 homologous repair (HR) genes, including *BRCA1/2*. This analysis is generated through a single genomic workflow. In detail, 50 ng of DNA was used for library preparation with the SOPHiA DDM HRD solution panel. This assay is a capture approach of 28 genes combined with the analysis of Whole Genome Sequencing (WGS) to quantify genomic aberrations. The genomic scar was calculated by an optimized analytic pipeline based on deep learning algorithms for the analysis of low-pass whole genome sequencing data. Sequencing was performed on the Illumina NextSeq 1000 platform (Illumina, San Diego, California, USA). HRD score was automatically calculated by SOPHiA DDM software which generates a GII score. A score > 0 was used as the cut-off to determine the genomic integrity. A sample with low genomic integrity results in a positive GII index. On the other hand, a sample with high genomic integrity results in a negative GII index. The HRD status combines the detection of pathogenic *BRCA* mutations and/or the GII index. SOPHIA GENETICS reports that the evaluation of its solution, defined from ovarian cancer samples, shows a concordance of 90% with the reference HRD method myChoice® CDx (Myriad Genetics, Inc., Salt Lake City, UT, USAC) (Data not published). Little data in the literature compares the test developed by SOPHiA GENETICS™ with the reference method myChoice® CDx (Myriad Genetics, Inc., Salt Lake City, UT, USAC). A recent study on 20 epithelial ovarian cancer samples showed a concordance rate of > 90.0% when they compared the SOPHiA GENETICS™ test to the myChoice® CDx test [41].

For each tumor sample, we present the HR status and associated score.

## Results

### Clinicopathologic characteristics

Patient enrollment comprised 25 high-grade EC cases over 18-months: 14 cases of UPSC, 8 cases of UCS, and three cases of mixed types. Our study specifically focused on high-grade tumors. Considering the reported annual incidence of approximately 33 new cases of EC in Martinique [1], the number of cases we included in this cohort over the specified timeframe appears to be representative of the expected caseload.

**Fig. 1:**
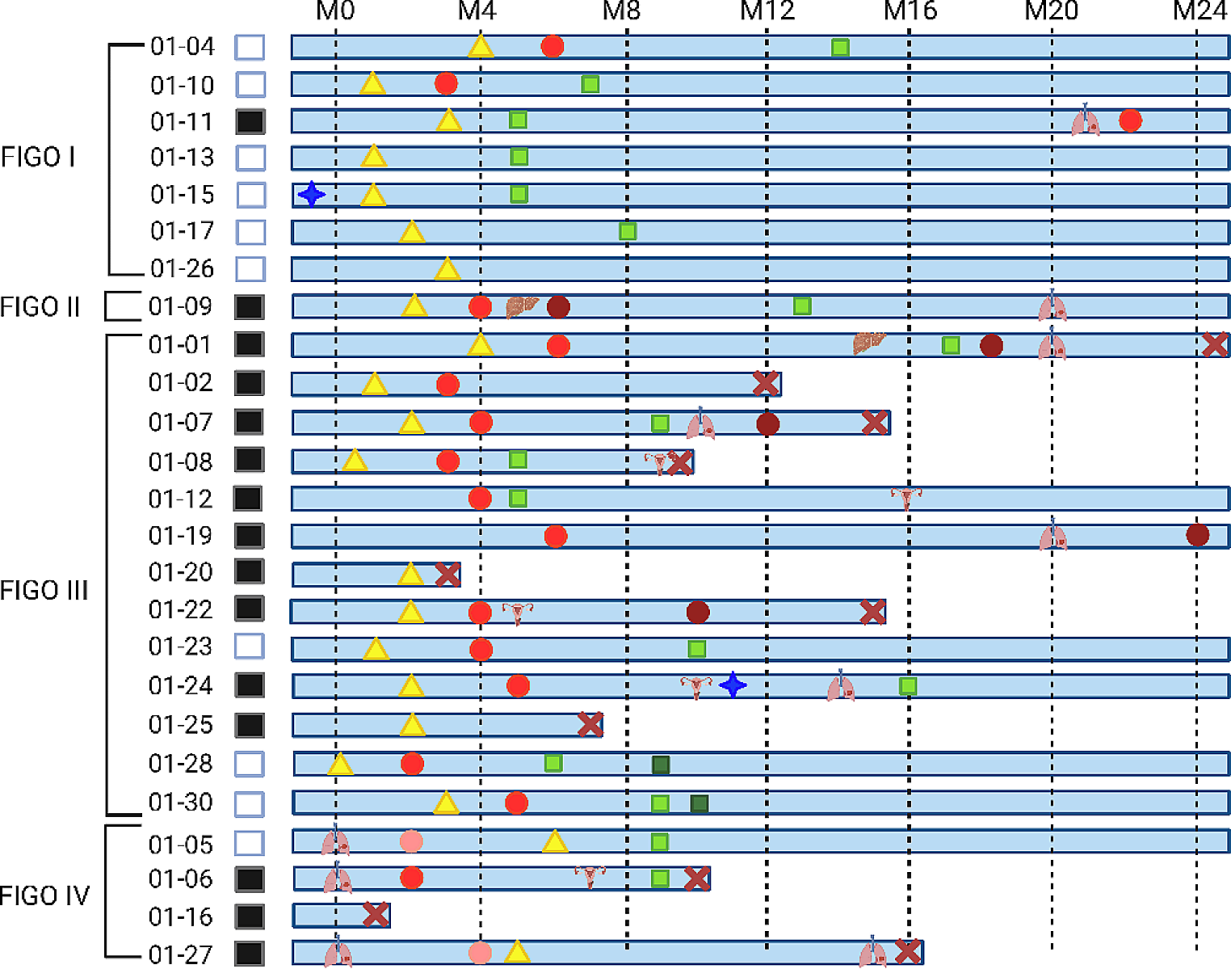
Clinical characteristics. Swimmer plot depicting the timing of following of 25 patients with high-grade endometrial cancer. The patients are grouped according the FIGO stage at diagnosis. The treatments are indicated by specific icon, surgery: yellow triangle, chemotherapy: red circle, radiotherapy and brachytherapy: green square, hormonotherapy: blue star. Recurrences are listed according to the organ affected. Deaths are marked with a red cross

We report most patients (71%) diagnosed at an advanced FIGO stage > II (Fig. 1). These patients have a poorest prognosis. Indeed, among the 18 patients diagnosed at an advanced stage, 14 underwent a recurrence or had a deterioration in their overall health. Conversely, of the 7 diagnosed at an early stage only one patient underwent disease progression with the onset of pulmonary lesions at M20.

Even though adjuvant CRT is the current recommended treatment for high-grade endometrial cancers, only 7 patients were able to receive this treatment, 2 at FIGO stage I (Endo-04 and Endo-10) and 5 at FIGO stage > II (Endo-07, Endo-08, Endo-23, Endo-28, Endo-30). Additionally, 5 other patients diagnosed at FIGO stage > II could not be treated after adjuvant chemotherapy (Endo-01, Endo-02, Endo-09, Endo-22, and Endo-24). Among those, 4 relapsed shortly after chemotherapy (Endo-02, Endo-09, Endo-22, and Endo-24).

Among the patients diagnosed at an advanced stage, we report 2 patients (Endo-16 and Endo-20) who experienced a rapid deterioration of their overall health followed by death. Similarly, Endo-25 died shortly after the surgery.

### Selection of copy-number-high category/TP53 tumors

**Fig. 2:**
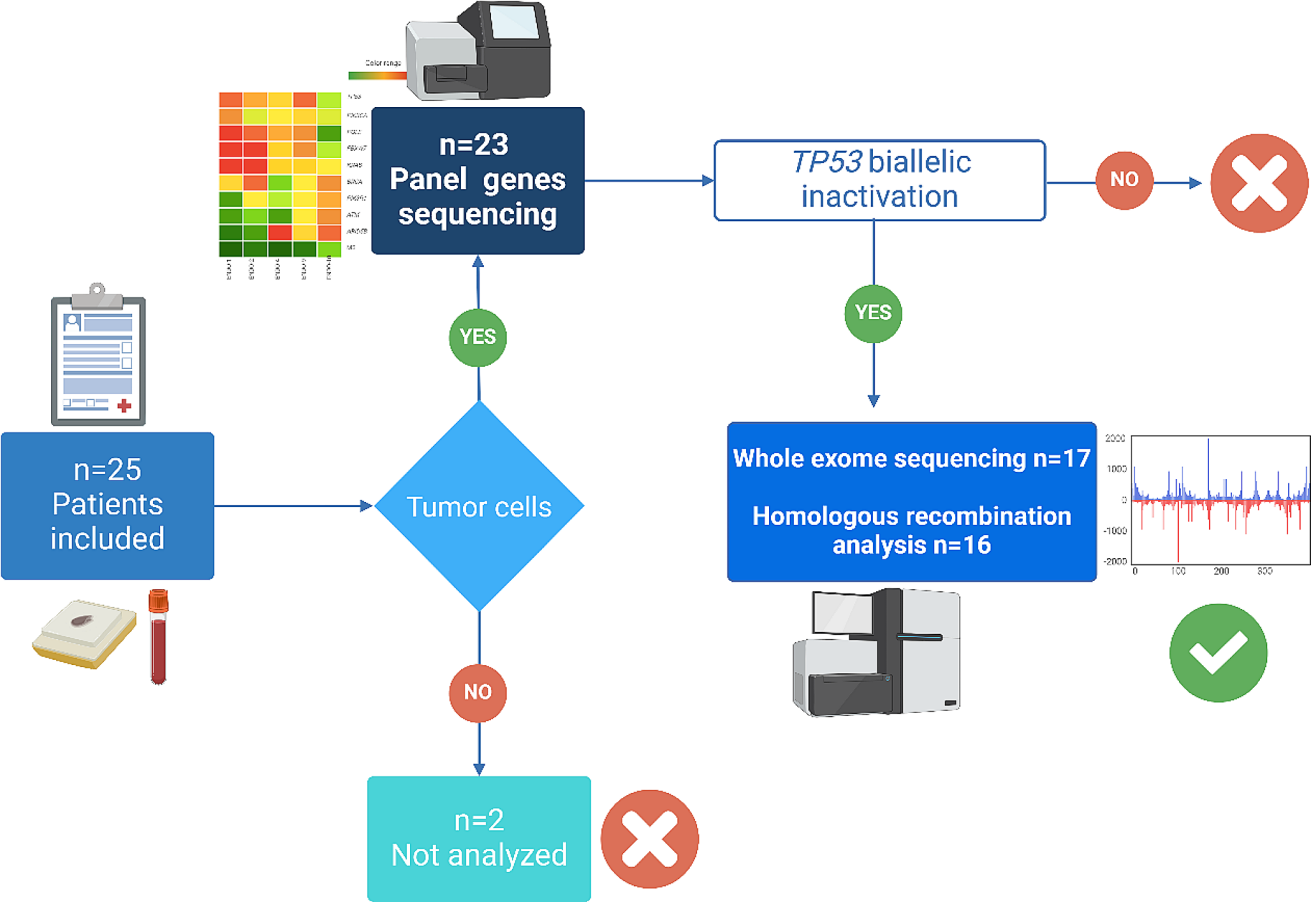
Patients and molecular analysis. 23 of the 25 included patients underwent sequencing analysis of a panel of 102 genes (Supplemental Table 1). Tumors carrying a *TP53* biallelic inactivation then underwent comprehensive genomic analysis, including Whole-exome Sequencing (WES) and Homologous Recombination (HR) analysis

A total of 23 patients underwent molecular analysis, including microsatellite testing and sequencing of a gene panel (Supplemental Table 1). Two patients were excluded due to the absence of tumor cells in the sample (Fig. 2). Among the tumors that underwent pangenomic analysis, our cohort consisted of 11 cases of UPSC, three cases of UCS, and three mixed types. The results of the microsatellite testing indicated that all tumors were proficient in mismatch repair (MMRp). Molecular characteristics of the tumors, focusing only on deleterious or probably deleterious variants, are presented in Table 1. Among these mutations, a *TP53* pathogenic mutation was identified in 21 tumors. These mutations are considered pathogenic, according to ClinVar, and all affect the DNA-binding domain. Hotspot mutations accounted for 26% (*n* = 5) of the identified mutations, with a variant allele frequency (VAF) greater than 0.01. The remaining mutations were infrequent or not described in the TGCA database (Table 1). Based on VIPUR predictions, all *TP53* missense variations were deemed deleterious, with confidence scores ranging from 0.63 to 0.99 (Table 2). However, VIPUR predictions suggested that the R248Q variant (structure-only confidence score: 0.33) and H179R variant (structure-only confidence score: 0.45) may have a more wild-type protein structure (Table 2). Nonetheless, the R248 residue directly interacts with DNA, and mutant alleles at position 248 are known to have reduced DNA-binding capacity. Similarly, the H179R variant near the zinc-binding site, is expected to fail in zinc binding. The wild-type p53 protein contains a zinc molecule that contributes to properly folding the DNA-binding domain. For all other missense variants, VIPUR predictions suggested an impact on protein folding. The p53 immunohistochemistry pattern also supported the functional effects of *TP53* missense mutations, as most tumors with missense variations exhibited p53 overexpression. Loss of protein expression in tumors was associated with truncating mutations (Table 2). Notably, VIPUR predicted that the R248Q and H179R mutations would have no impact on protein conformation. Interestingly, the p53 immunohistochemistry of the tumor with the R248Q variant showed a wild-type pattern. Furthermore, 18 tumors out of 21 with primary analysis exhibited loss of the wild-type *TP53* allele (Table 1), indicating that 86% of *TP53* mutated tumors experienced biallelic inactivation.

**Table 1:** Molecular characteristics

ID	Cell Tumor	Microsatellite	Genotype	Sequence	Consequence	Variant Allele
	Frequency	instability		Reference		Frequency
1	0.9	MSS	*TP53* c.722 C > G p.Ser241Cys	NM_000546.4	Missense	0.58
			*LRP1B* c.6291 C > A p.Tyr2097*	NM_018557	Nonsense	0.22
2	0.9	MSS	*TP53* c.839G > C Arg280Thr	NM_000546.4	Missense	0.95
			*PIK3CA* c.1133G > A p.Cys378Tyr	NM_006218.4	Missense	0.48
4	0.9	MSS	*SPOP* c.126_128delCTT F43del	NM_001007226	Frameshift	0.8
			*TP53* c.551_554delATAG D184fs	NM_000546.4	Frameshift	0.7
0.6	0.9	MSS	*TP53* c.462del p.Thr155Profr*15	NM_000546.4	Frameshift	0.7
			*PIK3CA* c.1035G > T p.Glu345Lys	NM_006218.4	Missence	0.7
7	0.5	MSS	*TP53* c.529_546del p.Pro177_Cys182del	NM_000546.4	Inframe	0.5
8	0.9	MSS	*TP53* c.743G > A Arg248Gln	NM_000546.4	Missense	0.9
9	0.8	MSS	*TP53* c.814G > A Val272Met	NM_000546.4	Missense	1
10	0.6	MSS	*TP53* c.743G > A Arg248Gln	NM_000546.4	Missense	0.9
11	0.8	MSS	*TP53* c.797G > A Gly266Glu	NM_000546.4	Missense	0.6
14	0.9	MSS	*PIK3CA* c.1635G > T Glu545Asp	NM_006218.4	Missense	0.93
			*KRAS* c.35G > A Gly12Asp	NM_033360	Missense	0.5
			*FGFR1* c.2123delT F708fs	NM_023110	Frameshift	0.42
15	0.5	MSS	*TP53* c.836G > A Gly279Glu	NM_000546.4	Missense	0.94
			*FBXW7* c.1394G > T Arg465Leu	NM_033632	Missense	0.48
16	0.1	MSS	*TP53* c.405 C > G Cys135Trp	NM_000546.4	Missense	0.35
			*FAT1* c.6188_6212 delCCCACGTTGTCGTGAAGGTCATTGT A2063fs	NM_005245|	Frameshift	0.1
17	0.9	MSS	*TP53* c.406 C > T Gln136*	NM_000546.4	Nonsense	0.5
19	0.8	MSS	*TP53* c.743G > A Arg248Gln	NM_000546.4	Missense	0.8
			*PIK3CA* c.1624G > A Glu542Lys	NM_006218.4	Missense	0.7
20	0.9	MSS	*TP53* c.814G > A Val272Met	NM_000546.4	Missense	0.35
22	0.7	MSS	*ATM* c.4852 C > T Arg1618*	NM_000051	Nonsense	0.8
			*PIK3CA* c.241G > A Glu81Lys	NM_006218.4	Missense	0.4
23	0.5	MSS	*TP53* c.524G > A Arg175His	NM_000546.4	Missense	0.9
24	0.8	MSS	*TP53* c.536 A > G His179Arg	NM_000546.4	Missense	0.8
25	0.9	MSS	*TP53* ex7 c.733G > A Gly245Ser	NM_000546.4	Missense	0.8
26	0.4	MSS	*TP53* ex7 c.733G > A Gly245Ser	NM_000546.4	Missense	0.2
			*KDR* c.2684_2701	NM_002253	Frameshift	0.1
			delATCTCAATGTGGTCAACC			
			His895_Asn900del			
27	0.6	MSS	*TP53* c.818G > A Arg273His	NM_000546.4	Missense	0.9
28	0.6	MSS	*TP53* c.824G > A Cys275Tyr	NM_000546.4	Missense	0.6
			*PIK3CA* c.1633G > A Glu545Lys	NM_006218.4	Missense	0.2
30	0.8	MSS	*TP53* c.797G > T Gly266Val	NM_000546.4	Missense	1

**Table 2:** VIPUR Predictions missense mutations

Variant	Label	Confidence	Structure-only	Structure-only	Sequence-only	Sequence-only	Exposure
	Prediction	Score	Label Prediction	Confidence Score	Label Prediction	Confidence Score	
TP53 C135W	deleterious	0.9958	deleterious	0.9802	deleterious	0.9304	interior
TP53 G279E	deleterious	0.9876	deleterious	0.9711	deleterious	0.8525	interior
TP53 C275Y	deleterious	0.9743	deleterious	0.8856	deleterious	0.9463	interior
TP53 G266V	deleterious	0.9640	deleterious	0.8929	deleterious	0.8970	interior
TP53 G266E	deleterious	0.8921	deleterious	0.9310	deleterious	0.8545	interior
TP53 H179R	deleterious	0.8556	neutral	0.4588	deleterious	0.8622	surface
TP53 V272M	deleterious	0.8198	deleterious	0.8024	deleterious	0.7462	interior
TP53 G245S	deleterious	0.8179	deleterious	0.6686	deleterious	0.8287	interior
TP53 R280T	deleterious	0.7989	deleterious	0.6000	deleterious	0.8512	surface
TP53 S241C	deleterious	0.7591	deleterious	0.6866	deleterious	0.7051	surface
TP53 R273H	deleterious	0.7426	deleterious	0.6933	deleterious	0.7160	surface
TP53 R248Q	deleterious	0.6309	neutral	0.3348	deleterious	0.7848	surface
TP53 R175H	deleterious	0.8317	deleterious	0.5867	deleterious	0.7561	interior

### Global pangenomic analysis

#### Identification of copy number signatures

**Fig. 3:**
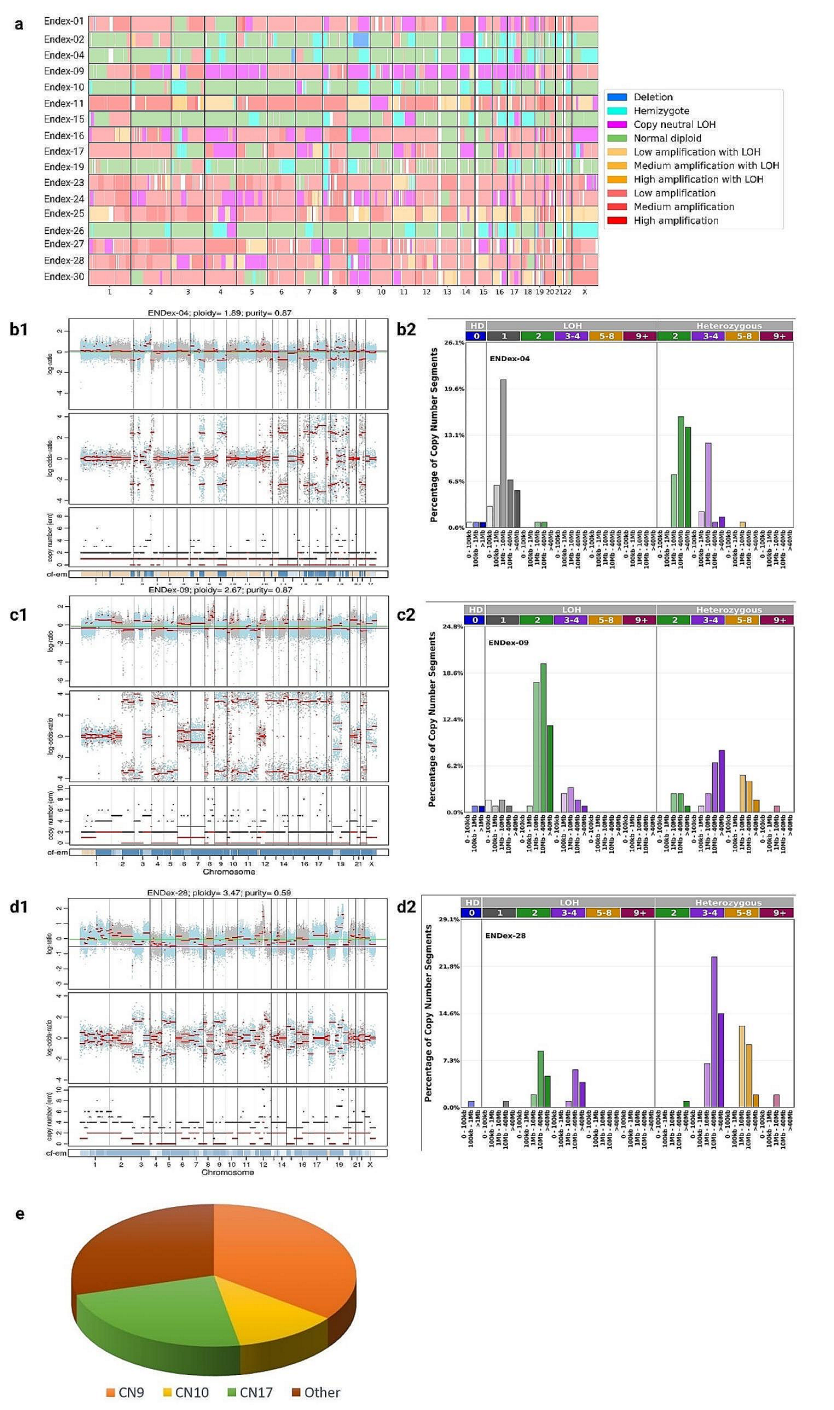
Copy numbers alteration of 17 high-grade EC with *TP53* mutation. **a** Global copy number signature for 17 endometrial tumors. **b** patterns of hypodiploidy with heterozygous diploid segments. **c** patterns of LOH with heterozygous duplicated segments. **d** patterns of heterozygous duplicated segments. **1** Plots generated by FACETS tool. **2** Plots generated by sigProfiler Script. **e** Distribution of signatures according Steele framework. (Note: Other copy number signatures are shown in Supplemental Fig. 1)

A concise overview of the copy number alterations of 17 tumors analyzed is presented in Fig. 3a. Six high-grade endometrial cancers (EC) harbor chromosomal rearrangements consisting mostly of Loss of Heterozygosity (LOH) segments with Total Copy Numbers (TCNs) between 1 and 2 (TCN1loh) and heterozygous segments with TCNs between 2 and 4 (TCN2het and TCN3-4het). These tumors are Endex-02, Endex-04, Endex-10, Endex-15, Endex-19, and Endex-26, which have been classified in the CN9 group. Two tumors, Endex-09, and Endex-30, had a rearrangement profile primarily involving copy-neutral LOH (TCN2loh and TCN3-4loh); the remaining genome was affected by heterozygous segments with low and medium duplication (TCN3-4het and TCN5-8het). They were categorized in the CN10 group. Five tumors (Endex-01, Endex-16, Endex-24, Endex-27, and Endex-28) fell into the CN17 group, characterized by predominantly duplicated heterozygous segments (TCN3-4het and TCN5-8het) with LOH segments (TCN2loh and TCN3-4loh). Finally, four tumors were placed in other signatures or were not categorized. These tumors had a genomic profile closely resembling the CN10 and CN17 signatures (duplicated heterozygous segments) (Supplemental Fig. 1).

### SBS, DBS, IDS signatures and TMB analysis

**Fig. 4:**
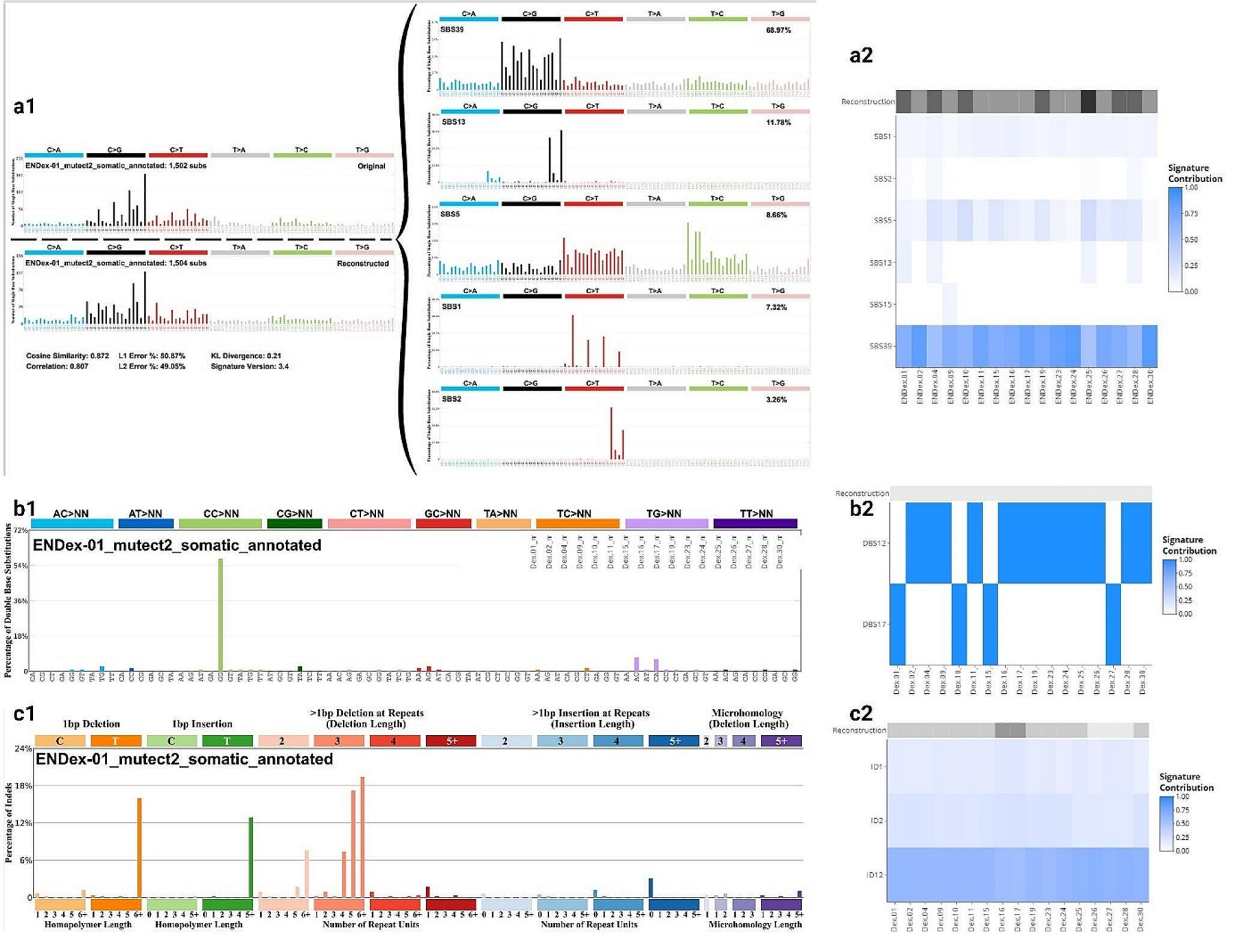
Pattern of signature. **a** Single-base substitution signatures. **b** Double-base substitution signatures. **c** Insertion/deletion signatures. **1** Plots generated by sigProfiler Script. **2** Signature for all patients (Note: Other copy number signatures are shown in Supplemental Figs. 2, 3 and 4)

Analysis of the SBS, DBS and IDS signatures, using the tool developed by Alexandrov [40], suggests that the molecular profile of the variants is homogeneous across the 17 tumors analyzed. Indeed, we detected the SBS39 signature in all tumors, DBS12 in 70% of tumors and ID12 as the majority signature in all tumors (Fig. 4; Supplemental Figs. 2, 3 and 4). SBS39 are presented by predominantly C > G transversion. DBS12 is characterized by mutations of CG dinucleotides and significantly correlated with SBS39 signature [40]. ID12 is characterized by deletions of more than one base in the repeat unit. Additionally, all tumors analyzed were associated with moderate TMB (median = 38.7 Mutations/Mb; range = 23.8–50.9) (Supplemental Fig. 5).

### Signatures and mutational processes

**Fig. 5:**
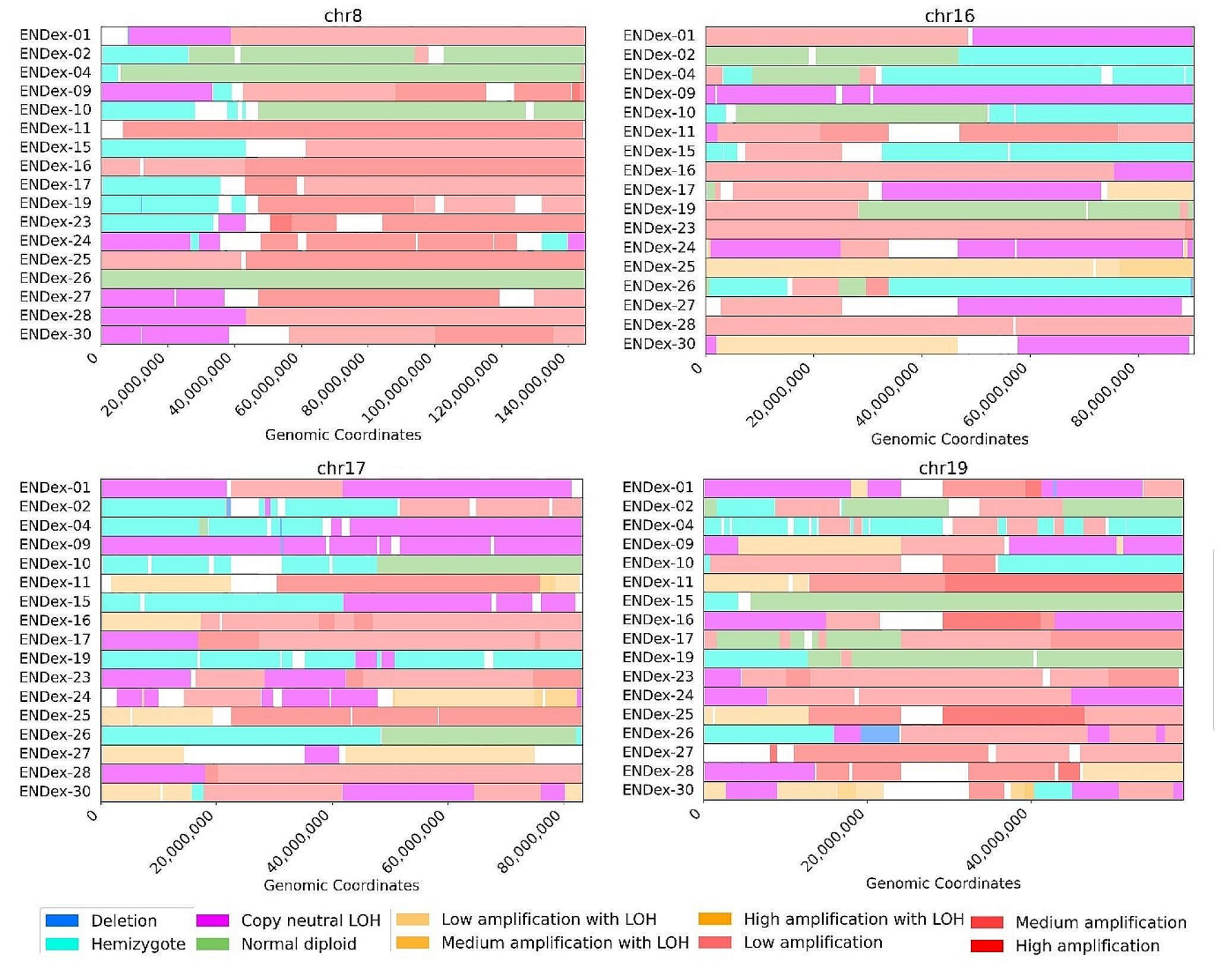
Common alterations to 17 high-grade endometrial tumors. Plot of chromosomes 8, 16, 17 and 19. Visualization of deleted regions of 8p, 16q and 17p and amplified region of chromosome 19

The CN9 and CN10 signatures indicate of a type of structural chromosomal instability (CIN) often induced by replication stress [42]. Replication stress encompasses events that contribute to obstacles in replication forks, potentially leading to stalled forks. Cleavage of stalled forks can cause fork collapse and generate double-strand breaks (DSBs), contributing to CIN [42–44]. Two observations support the connection between replication stress and the oncogenesis of other tumors in our analysis: (i) identification of deletions in regions 8p, 16q, and 17p in most tumors, and (ii) amplification of 19q12 in most tumors, including *CCNE1* (Fig. 5; Table 3). CCNE1 positively regulates the cell cycle transition from the G1 to S phase. Dysregulation of CCNE1 can promote premature entry into the S phase, leading to increased replication forks and DSBs [45–47]. In our cohort, we noted amplification of the 19q12 region in 12 tumors with *CCNE1* amplification (chr19:29,811,991 − 29,824,312), accounting for 70% of cases (Table 3). Interestingly enough, among the 5 tumors without *CCNE1* amplification, one harbored the *FBXW7* c.1394G > T Arg465Leu mutation described as pathogenic (Table 1). This hotspot mutation is located in the FBXW7 WD40 domain [48] and may be important for proper folding of the substrate binding pocket. Computational analysis showed a significant deviation in structural configuration and stability of FBXW7 mutants R465H. The FBWX7 protein being known to act as a negative regulator of CCNE1 activity by binding directly to CCNE1 and targeting it for ubiquitin-mediated degradation [49], a deleterious mutation of *FBXW7* could alter FBXW7-CCNE1 interaction, leading to phenocopy of *CCNE1* amplification [50]. Additionally, three other tumors without *CCNE1* amplification carry a *PIK3CA* mutation (Table 1). Our results are consistent with data from TGCA database. Indeed, in the population of African descent, the alterations most frequently encountered in endometrial tumors with *TP53* mutation are *PIK3CA* mutation, *CCNE1* amplification and *FBXW7* mutation in 25%, 20% and 19% respectively. According to this database, the distribution of the most frequent variants is not the same in the Caucasian population, with the most frequent alterations affecting the *PIK3CA*, *CASP8AP2* and *TTN* genes, and a 10% amplification rate for the *CCNE1* gene.

**Table 3:** Copy number alterations

ID	Copy number signature	CCNE1 (amplified locus)	19q12	8p	16q	17p	Single-base substitution signature	Status HRD	HRD GII
Endo-01	CN17	chr19:29207561–39,335,510	Medium amplification	cn-LOH	cn-LOH	cn-LOH	SBS2 SBS13 SBS1	Negative	-2
Endo-02	CN9			Hemizygote deletion	Hemizygote deletion	Hemizygote deletion	SBS3 SBS1	Negative	-6
Endo-04	CN9	chr19:29207593–35,944,857	Low amplification	Hemizygote deletion	Hemizygote deletion	Hemizygote deletion	SBS2 SBS3	Negative	-10
Endo-09	CN10	chr19:24127972–36,748,376	Low amplification	cn-LOH	cn-LOH	cn-LOH	SBS1 SBS3	Negative	-3,4
Endo-10	CN9	chr19:29207671–35,642,626	Medium amplification	Hemizygote deletion	Hemizygote deletion	Hemizygote deletion	SBS5 SBS1	Negative	-10,4
Endo-11	NA	chr19:12885927–58,572,600	Medium amplification			cn-LOH	SBS3 SBS5	Negative	-4,9
Endo-15	CN9			Hemizygote deletion	Hemizygote deletion	Hemizygote deletion	SBS3 SBS1	Negative	-17,2
Endo-16	CN17	chr19:29207561–41,124,804	High amplification		cn-LOH	Amplification with LOH	SBS3 SBS1	NA	NA
Endo-17	NA	chr19:24127741–42,403,032	Low amplification	Hemizygote deletion	cn-LOH	cn-LOH	SBS3 SBS13	Negative	-6,7
Endo-19	CN9			Hemizygote deletion		Hemizygote deletion	SBS3 SBS13	Negative	-11,9
Endo-23	NA	chr19:13073088–41,439,890	Low amplification	Hemizygote deletion		cn-LOH	SBS3 SBS6	Negative	-10
Endo-24	CN17	chr19:18995149–44,916,309	Low amplification	cn-LOH		cn-LOH	SBS3 SBS12	Negative	-0,2
Endo-25	CN18	chr19:29207641–46,608,866	High amplification		Amplification with LOH	Amplification with LOH	SBS3 SBS13	Negative	-4,5
Endo-26	CN9	chr19:24087318–46,919,400	Low amplification		Hemizygote deletion	Hemizygote deletion	SBS3 SBS1	Negative	-13,4
Endo-27	CN17	chr19:11025540–34,767,319	Medium amplification	cn-LOH	cn-LOH	Amplification with LOH	SBS3 SBS1	Negative	-3,7
Endo-28	CN17			cn-LOH		cn-LOH	SBS3 SBS2	Positive	0,9
Endo-30	CN10			cn-LOH	cn-LOH	Amplification with LOH	SBS3 SBS1	Negative	-6,3

Data availability statements

The data used to support the findings of this study are included within the article, or available from the corresponding author upon request

Activated oncogenes were described as promoting replication stress. Alteration of these oncogenes, like *CCNE1* amplification, could generate DSBs and account for the genomic rearrangements identified in our tumors. The frequently deleted regions 8p, 16q, and 17p (Fig. 5; Table 3) can be considered common fragile sites (CFSs) [43]. DNA strand breaks often occur during replication stress in specific regions known as CFSs. Intriguingly, these regions are also found to be deleted in UPSCs according to the TCGA database (Supplemental Fig. 6). Usually, a well-described mechanism detects and repairs stalled and collapsed replication forks. However, a double-strand break repair system must be activated when breaks occur. We hypothesized that the intervention of different double-strand break repair mechanisms—homologous recombination (HR) repair, single-strand annealing (SSA), alternative end-joining (alt-EJ), and non-homologous end-joining (NHEJ)—could explain the different signatures observed. Each pathway generates distinct genomic rearrangements (deletions or insertions), leading to varying consequences for genome integrity. HR is a high-fidelity repair system that copies an intact homologous sequence, thereby preserving genome stability. This mechanism may underlie the formation of copy-neutral loss of heterozygosity (cn-LOH). SSA involves the single-strand end joining of homologous repeated regions, potentially leading to large deletions. Finally, the alt-EJ mechanism, involving repeated micro-homologies, leads to insertion/deletion-type rearrangements. The SSA and HR pathways could elucidate groups with predominant loss of heterozygosity (LOH) segments, such as the CN9 signature, and copy-neutral LOH, like the CN10 signature. The involvement of the Break-induced Replication (BIR) pathway, a subtype of the HR pathway, could elucidate the CN17 signature phenotype. This subtype has been linked to the repair of collapsed replication forks, leading to duplications and rearrangements [51, 52].

Analysis of the other signatures is consistent with the involvement of replication stress in the oncogenesis of the tumors analyzed. The SBS39 signature could be a consequence of the involvement of replication stress. This signature has been attributed to the involvement of error-prone DNA polymerases [53] such as translesion synthesis (TLS) polymerase [54]. As these polymerases are characterized by low fidelity, they introduce mutations [54–56], contributing to the increase in the rate of variation. A recent review has confirmed the involvement of TLS in replication stress, suggesting that it enables replication to continue, preventing the emergence of broken forks or facilitating their repair after collapse [57]. The increased error rate due to TLS activity could explain the TMB results, which describe a mutation rate between 23.8 and 50.9 Mutations/Mb (Supplemental Fig. 5). A recent study incriminated replication stress induce by oncogenes in genomic instability and increased tumors mutational burden [58]. Interestingly, the DBS12 signature has been associated with SBS39 [40], confirming the consistency of the different signatures detected in the 17 tumors analyzed, despite their unknown etiology.

### Signatures of the number of copies and involvement of repair DNA pathways

We investigated into whether deleterious gene mutations associated with repair pathways could account for the observed signature variations. Prior investigations have highlighted an overexpression of the CN17 signature in samples harboring germline and/or somatic mutations in pivotal homologous recombination (HR) genes, such as *BRCA1*, *BRCA2*, *PALB2*, and *CDK12* [20]. Nonetheless, our analysis detected no mutations in any of the HR genes. This observation is further substantiated by the homologous recombination deficiency (HRD) analysis, where most tumors exhibit a genomic profile indicative of homologous recombination proficiency. The HRD scores span from − 17.2 to + 0.9 (Table 3), and only one tumor (Endex-28) demonstrated homologous recombination deficiency, as indicated by a GII index of + 0.9. Notably, no deleterious mutations were identified in the 28 homologous repair (HR) genes within this specific tumor. Of interest, four tumors exhibited HRD scores close to the defined threshold. These tumors were classified in the CN17 group, which has been previously described as enriched with HRD cases [20]. It is important to note that tumors with deleterious mutations in HRD-associated genes have the highest HRD scores [59].

### Copy number signature and clinicopathologic characteristics

**Fig. 6:**
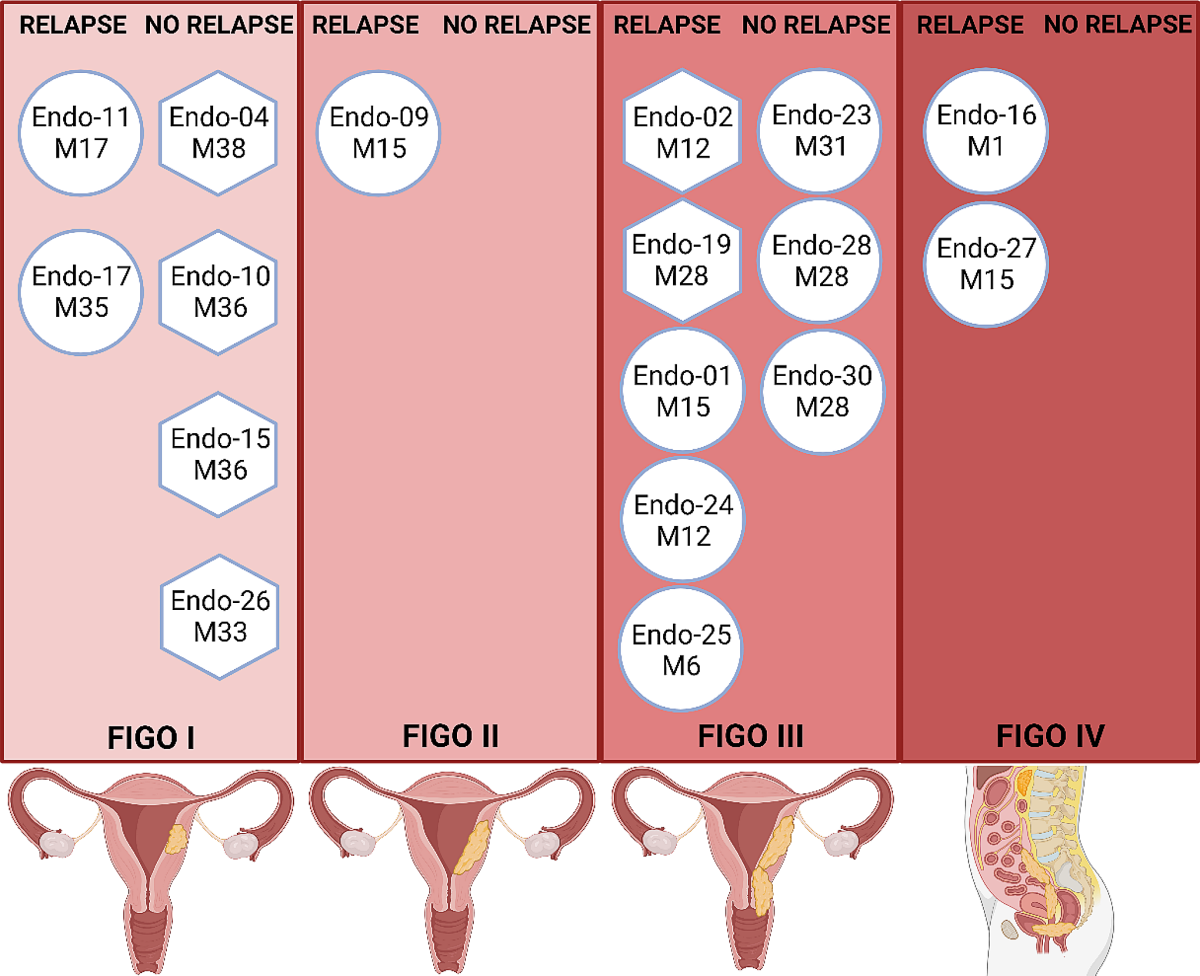
Clinicopathologic characteristics and copy number signatures. Classification of 17 high-grade endometrial tumors according to stage. Hexagonal forms represent tumors with the most deleted segments (CN9). Circle forms represent tumors with the most amplified segments (CN10, CN17 and others). For relapse, M is the time between diagnosis and clinical relapse. For no relapse, M is the study participation time

We have classified the CNV signatures into two main groups, the first characterized by a genomic profile harboring a majority of deleted segments (CN9) and the second by a genomic profile harboring a majority of amplified segments such as CN10 and CN17. Although our study involved only a limited number of patients, we evaluated the utility of signatures in predicting tumor progression. This study suggests that the copy number signature framework outlined by Steel [20] could hold promise in predicting outcomes for tumors diagnosed at an early stage. As depicted in Fig. 6, tumors diagnosed at an early stage and associated with a CN9 signature demonstrated a favorable prognosis. Notably, none of the tumors—Endo-04, Endo-10, Endo-15, and Endo-16—diagnosed at FIGO stage I experienced recurrence after 36 months of follow-up. These tumors exhibit a genomic profile characterized by deleted segments. Conversely, tumors Endo-11 and Endo-17, which possess a profile featuring amplified segments, experienced recurrence at M17 and M35, respectively, despite their early stage diagnosis. It’s worth highlighting that a notable number of tumors with a signature predominantly marked by amplified segments (Endo-01, Endo-09, Endo-11, Endo-16, Endo-17, Endo-24, Endo-25, and Endo-27) demonstrated a poor prognosis, with recurrences.

Given our cohort’s and the study’s duration, these findings are primarily observational. Nonetheless, they suggest the potential applicability of copy number signatures in managing high-grade endometrial tumors in Martinique. A recent report emphasized that 3 out of 4 high-grade endometrial tumors were diagnosed at an advanced stage [1]. Genomic stratification of these tumors could provide additional for improved management. Tumors characterized by an amplified genome might become targets for innovative targeted therapies.

## Discussion

High-grade ECs are known for their poor prognosis, regardless of the histological subtype, particularly in Martinique, where we recently reported an elevated incidence and greater challenges in their management compared to mainland France [1]. Our study demonstrates the utility of utilizing the copy number signature tool provided by COSMIC to classify tumors. Indeed, for the first time, we associate CN9 and CN10 signatures with high-grade EC. We also identify other CNV signatures. All these signatures appear to be associated with tumor stage that may carry prognostic value. The challenging prognosis of high-grade ECs, regardless of the histological subtype, is emphasized, particularly in regions like Martinique where management difficulties are reported. Despite the molecular classification allowing the categorization of high-grade tumors into a TP53-mutated subgroup [4], effective therapeutic management remains elusive. Stratifying these high-grade tumors based on their copy number signatures and detecting intricate molecular rearrangements, could offer improved management approaches. The study suggests that despite different copy number signatures, the underlying oncogenic mechanism for high-grade endometrial tumors might be shared, with replication stress as a pivotal factor in oncogenesis. *CCNE1* amplification emerges as a driving force in over 70% of tumors, rendering it a potential target for novel therapies. CCNE1 overexpression in tumors has been associated with platinum resistance [60, 61], potentially accounting for the limited success of existing treatments for high-grade EC. *CCNE1* encodes cyclin E protein, interacting with CDK2 to facilitate cell-cycle progression from G1 to S phase. Overexpression of cyclin E1 seems to hinder replication, leading to conflicts between replication and transcription mechanisms [62], ultimately causing replication stress. CCNE1 overexpression is linked to genomic instability, highlighting the importance of S and G2/M checkpoints for cell survival [63]. Emerging therapies targeting the dependency on S and G2/M checkpoints for cell survival have shown promise. Recent studies indicate that CCNE1 overexpression enhances sensitivity to replication checkpoint inhibitors. Targeting effectors of replication checkpoints like ATR, CHK1, and WEE1 have exhibited potential in cells with *CCNE1* amplification. These proteins are part of the ATR/CHK1/WEE1 pathway, responsible for sensing DNA single-strand breaks and replication stress. ATR, crucial for checkpoint transitions in S and G2/M phases, is recruited at DSBs to slow down and stabilize replication forks, preventing their collapse [64] by phosphorylating CHK1. Phosphorylated CHK1 inhibits its substrates, CDC25C and CDC25A phosphatases, leading to the arrest of the G2/M transition [65–67]. WEE1, a serine-threonine kinase, regulates the G2/M checkpoint transition [62, 68, 69]. WEE1 induces G2/M arrest by inhibitory phosphorylation of CDK1 and CDK2, preventing mitosis entry for DNA repair during damage [62, 68, 69]. Enhanced sensitivity to ATR, CHK1, and WEE1 inactivation has been demonstrated in cells with *CCNE1* amplification [63, 70, 71]. Inhibiting ATR, CHK1, or WEE1 impacts cell cycle progression, exacerbating mitotic aberrations induced by Cyclin E1 overexpression, leading to cytotoxicity and cell death [63, 72]. Moreover, in vivo evaluation of combining these inhibitors on endometrial cell lines demonstrated antitumor effects, especially in cells with *TP53* alterations. Notably, all tumors benefiting from pangenomic analysis that identified *CCNE1* amplification carried bi-allelic *TP53* inactivation, suggesting potential interest in these inhibitors. *TP53* is now considered a barrier against oncogene-induced DNA damage due to replication stress, leading to apoptosis or senescence. While not the driver of tumor initiation and progression, TP53 offers a proliferative advantage under selective pressure [43]. Given *CCNE1* amplification’s prevalence in over half of analyzed high-grade endometrial tumors, it’s conceivable to consider these molecules in strategies for managing such tumors [72, 73]. Several of these inhibitors are undergoing clinical investigation [72, 74, 75]. While the outcomes of these clinical trials are significant, it will be crucial to differentiate the subgroup of tumors carrying *CCNE1* amplification and *TP53* alteration. Cell vulnerability to cell cycle checkpoint inhibitors is oncogene-dependent. Furthermore, a Phase I trial analysis of a WEE1 inhibitor exhibited a response in two ECs with *CCNE1* mRNA overexpression [76].

Although *CCNE1* amplification wasn’t detected in all tumors analyzed, our findings suggest that most tumors might be sensitive to cell-cycle checkpoint inhibitors. In fact, 90% of tumors show a copy number signature indicating of replication stress, along with the detection of common region deletions referred to as CSFs. These therapies aim to induce mitotic catastrophe by exacerbating DNA alterations, leading to cell death in cells with genomic instability.

This study underscores the benefits of employing multiple pangenomic analyses. Even though we propose replication stress’s involvement in the oncogenesis of all tumors, it is important to combine the results of other signatures, such as HRD scores close to the threshold cycle or SBS39 signatures and TMB, which indicates a high level of mutations. In fact, all these results suggest that it is possible to combine a cell cycle checkpoint inhibitor with another therapy as PARPi or immunotherapy. A recent study demonstrated combinatorial efficacy with an ATR inhibitor and Olaparib [70]. In cells harboring potential replication stress drivers like *CCNE1* amplification, antitumor activity appears effective when combined with simultaneous inhibition of homologous recombination and the PARP repair pathway [70].

While our study has added valuable insights, the cohort’s size prevented us from defining distinct copy number signature subgroups precisely. Associations between clinical and biological features with each subgroup, such as histological type, stage, or prognosis, couldn’t be established. More extensive association studies are warranted. To better understand the oncogenesis of all endometrial tumors, particularly those with a favorable initial prognosis and rapid evolution, a complementary study is needed, including all tumors diagnosed in Martinique. These additional studies will be essential to improve the management of high-grade ECs. However, genome-wide analyses are not feasible for diagnostic purposes, as they require considerable resources. It is therefore essential to identify markers that are specific to each signature and easy to detect. The identification of *CCNE1* gene amplification and *PIK3CA* mutations, using more common laboratory techniques, could provide an alternative means of identifying replication stress, and be sufficient to guide patients towards therapeutic strategies.

## Conclusions

Our study highlights the importance of investigating the molecular mechanisms involved in oncogenesis in the Martinique population. Despite being a French department, this population possesses a genetic heritage from the African population. Furthermore, environmental factors such as exposure to specific pesticides and lifestyle factors likely contribute to the activation of oncogenic mechanisms unique to this population. The discovery of molecular markers that facilitate the characterization of the underlying causes of oncogenesis has ushered in targeted therapies that significantly advance the management of specific tumors [77–80]. Identifying the molecular mechanisms involved in the oncogenesis of endometrial tumors in Martinique is fundamental to optimizing patient care. The discovery of *CCNE1* amplification in most high-grade endometrial tumors provides a promising avenue for therapy. *TP53* mutations, often associated with this *CCNE1* amplification, cannot serve as the therapeutic target for these tumors. However, it could signify the involvement of replication stress in initiating oncogenesis. The identification of supplemental markers could help steer therapeutic strategies.

## Electronic supplementary material

Below is the link to the electronic supplementary material.

Supplementary Material 1

Supplementary Material 2

Supplementary Material 3

Supplementary Material 4

Supplementary Material 5

Supplementary Material 6

Supplementary Material 7

## Data Availability

No datasets were generated or analysed during the current study.
