# Supplementary material for: Copy number signatures and CCNE1 amplification reveal the involvement of replication stress in high-grade endometrial tumors oncogenesis
Source: Cell Oncol (Dordr). 2024 Apr 2;47(4):1441–57. doi: 10.1007/s13402-024-00942-w (PMC11322381; doi:10.1007/s13402-024-00942-w)
Supplement: Supplementary file 3 — Supplementary Material 3 [file 13402_2024_942_MOESM3_ESM.pdf]

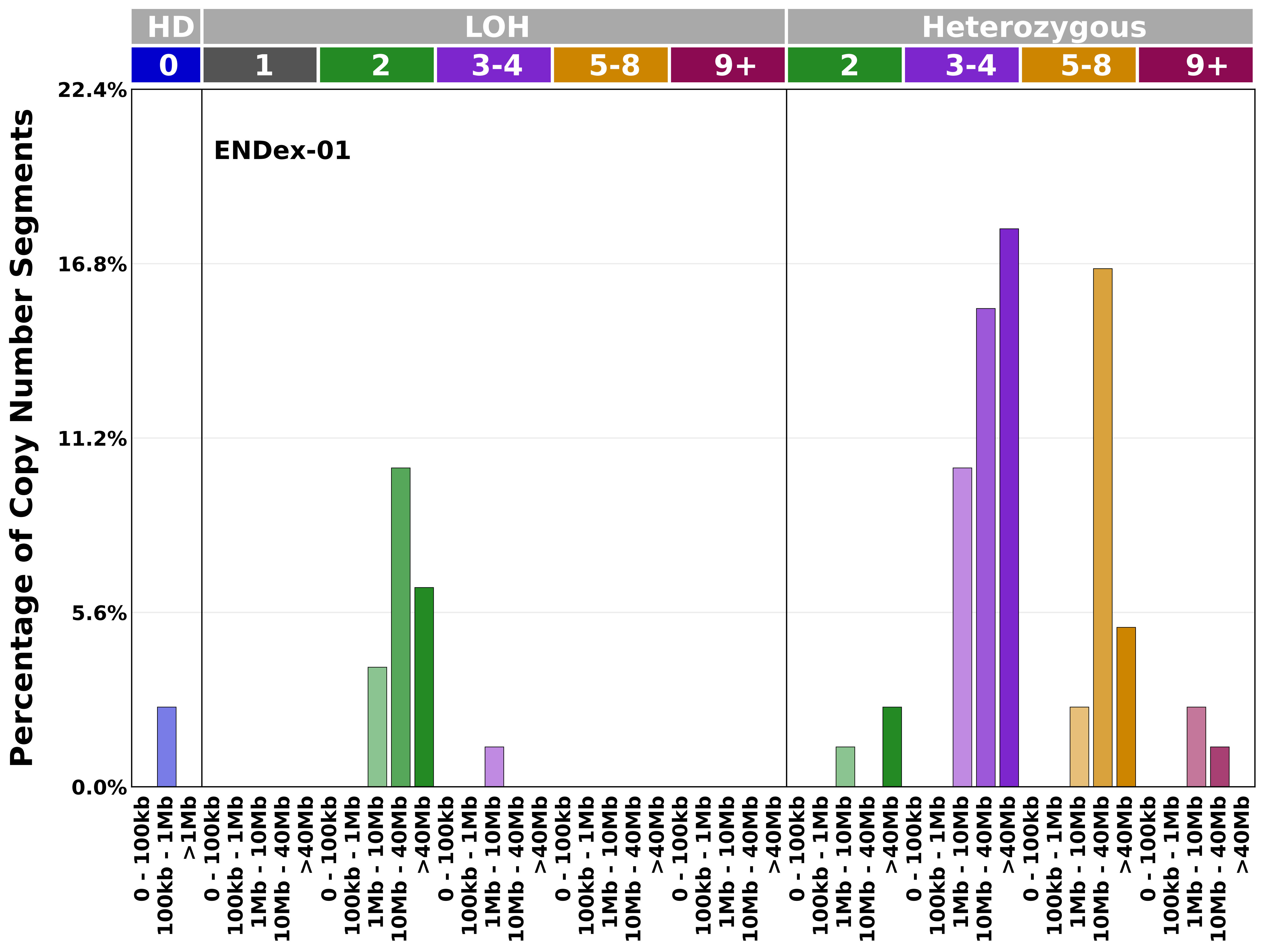
HD
LOH
Heterozygous
0
1
2
3-4
5-8
9+
2
3-4
5-8
9+
ENDex-01
Percentage of Copy Number Segments
22.4%
16.8%
11.2%
5.6%
0.0%
0 - 100kb
100kb - 1Mb
>1Mb
1Mb - 10Mb
10Mb - 40Mb
>40Mb

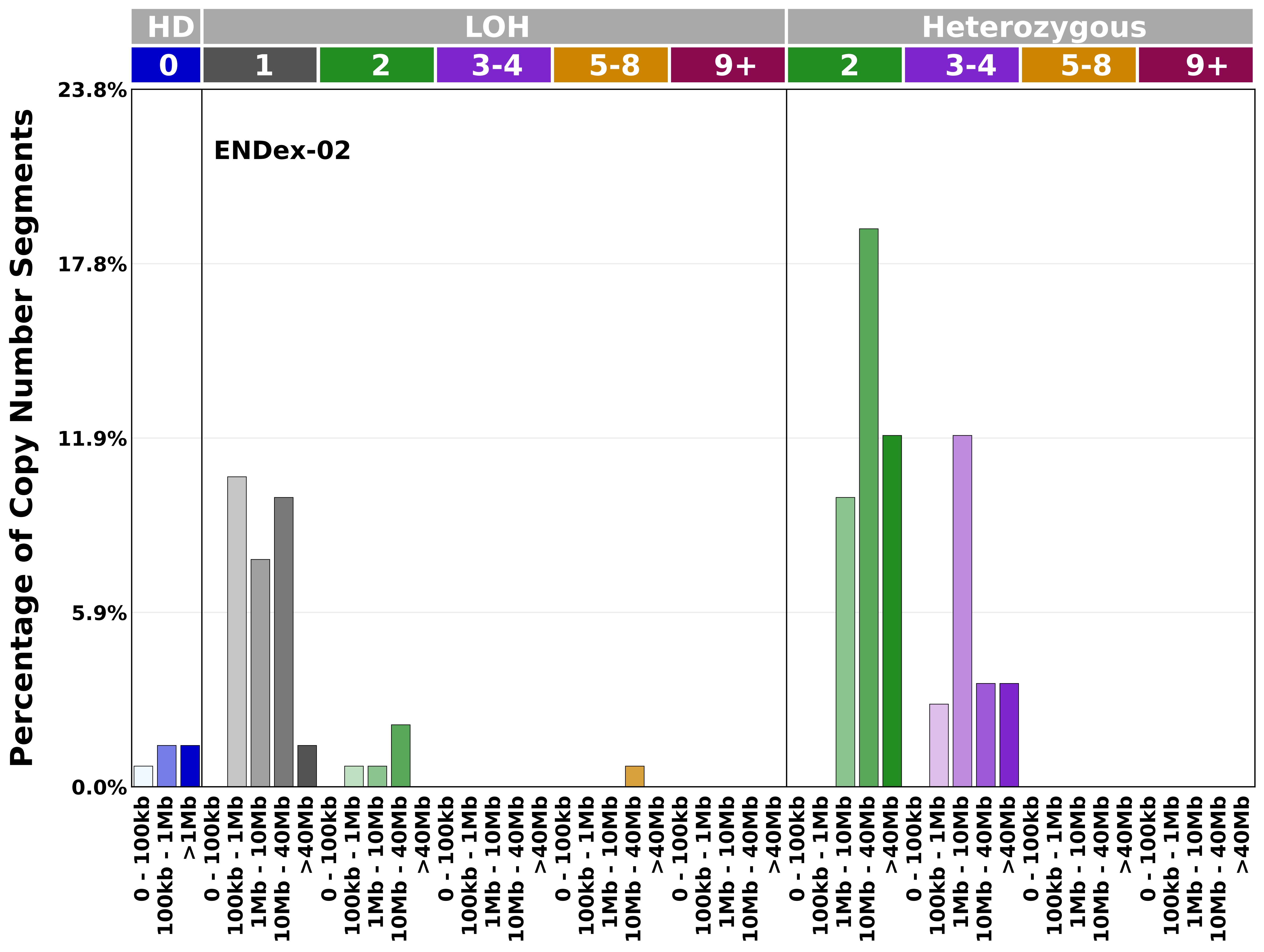

HD
LOH
Heterozygous
0
1
2
3-4
5-8
9+
2
3-4
5-8
9+
ENDex-02
Percentage of Copy Number Segments
23.8%
17.8%
11.9%
5.9%
0.0%
0 - 100kb
100kb - 1Mb
>1Mb
1Mb - 10Mb
10Mb - 40Mb
>40Mb

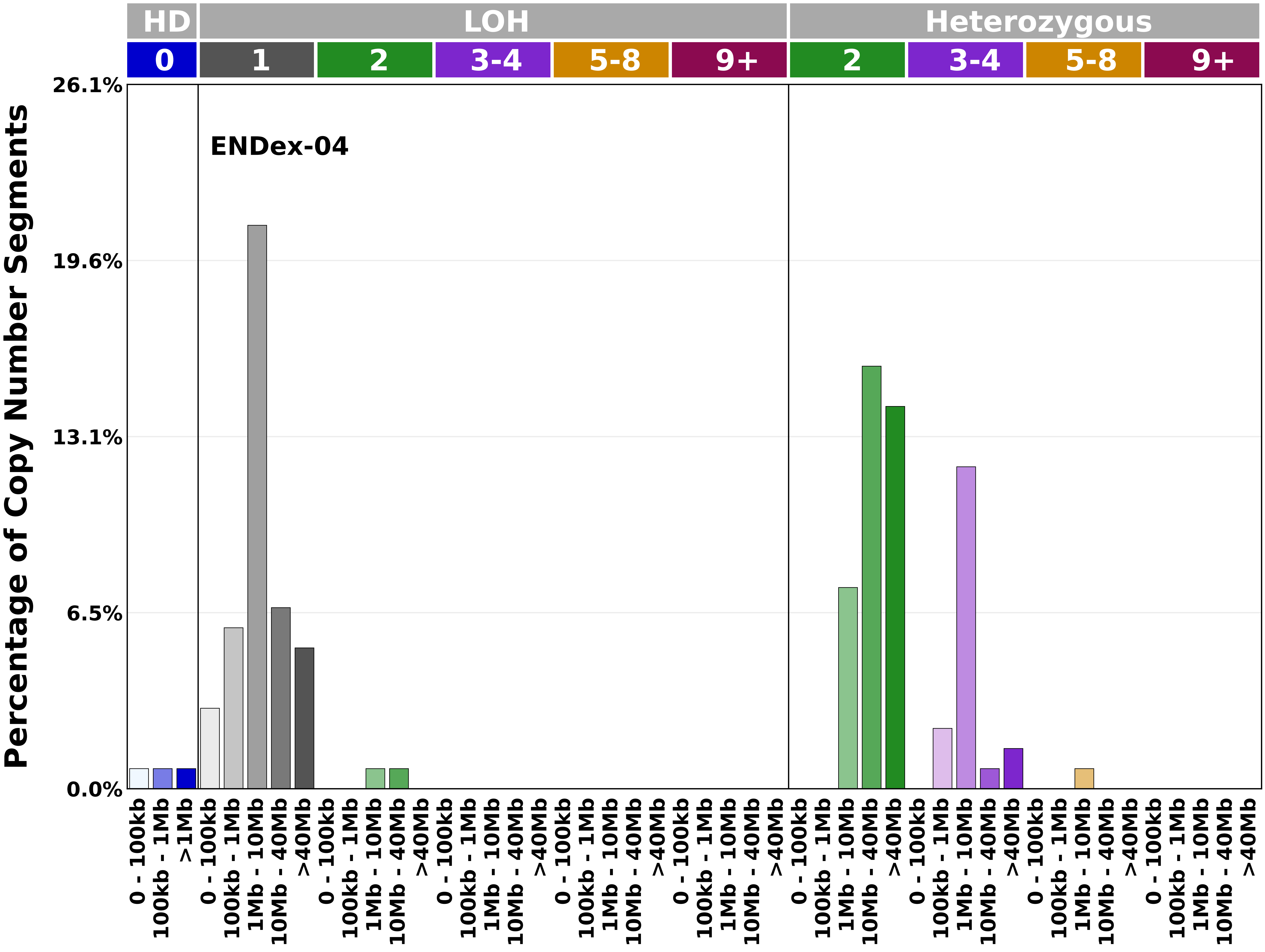

HD
LOH
Heterozygous
0
1
2
3-4
5-8
9+
ENDex-04
Percentage of Copy Number Segments
26.1%
19.6%
13.1%
6.5%
0.0%
0 - 100kb
100kb - 1Mb
>1Mb
1Mb - 10Mb
10Mb - 40Mb
>40Mb

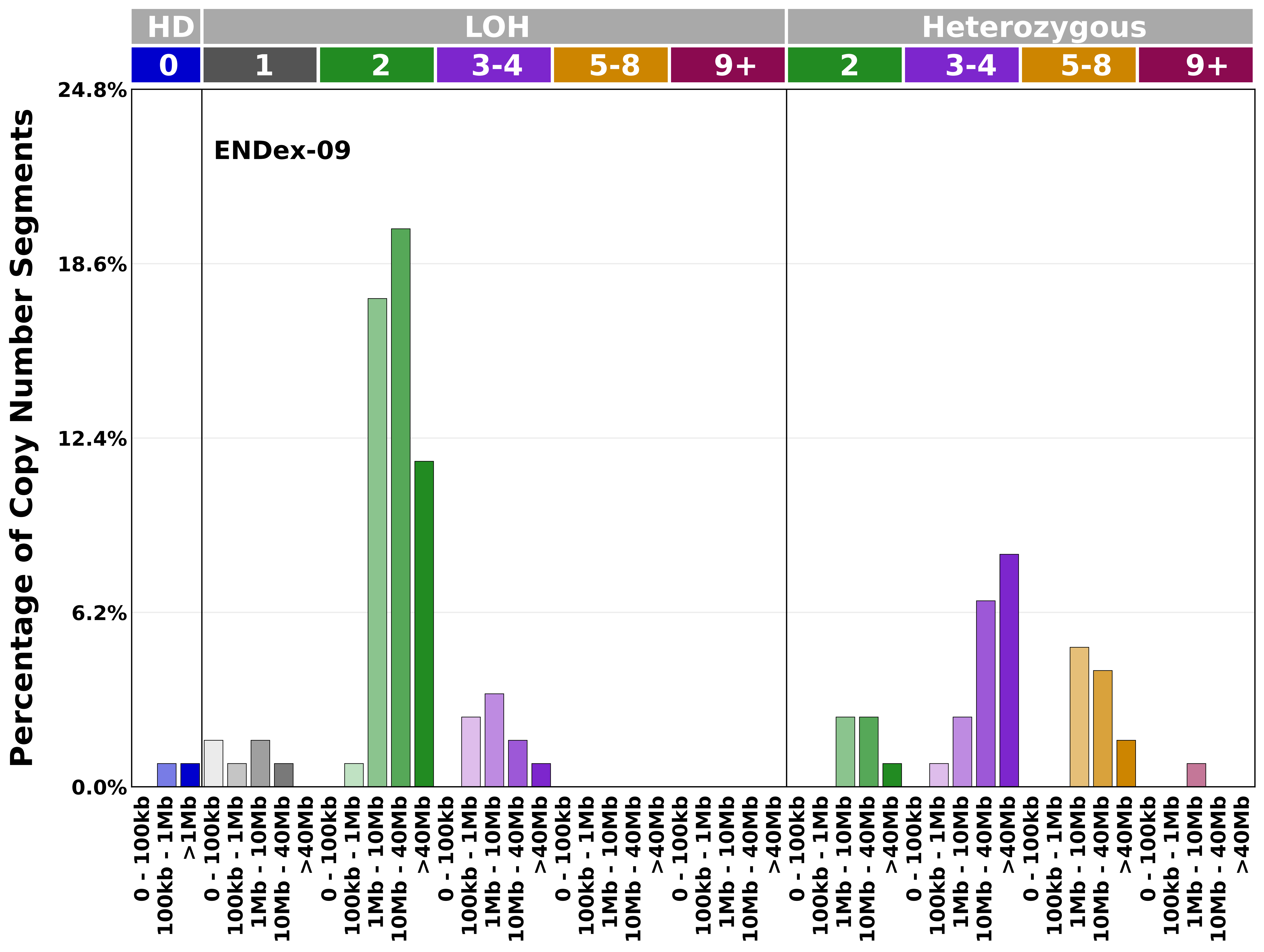
HD
LOH
Heterozygous
0
1
2
3-4
5-8
9+
2
3-4
5-8
9+
24.8%
18.6%
12.4%
6.2%
0.0%
Percentage of Copy Number Segments
ENDex-09
0 - 100kb
100kb - 1Mb
>1Mb
1Mb - 10Mb
10Mb - 40Mb
>40Mb

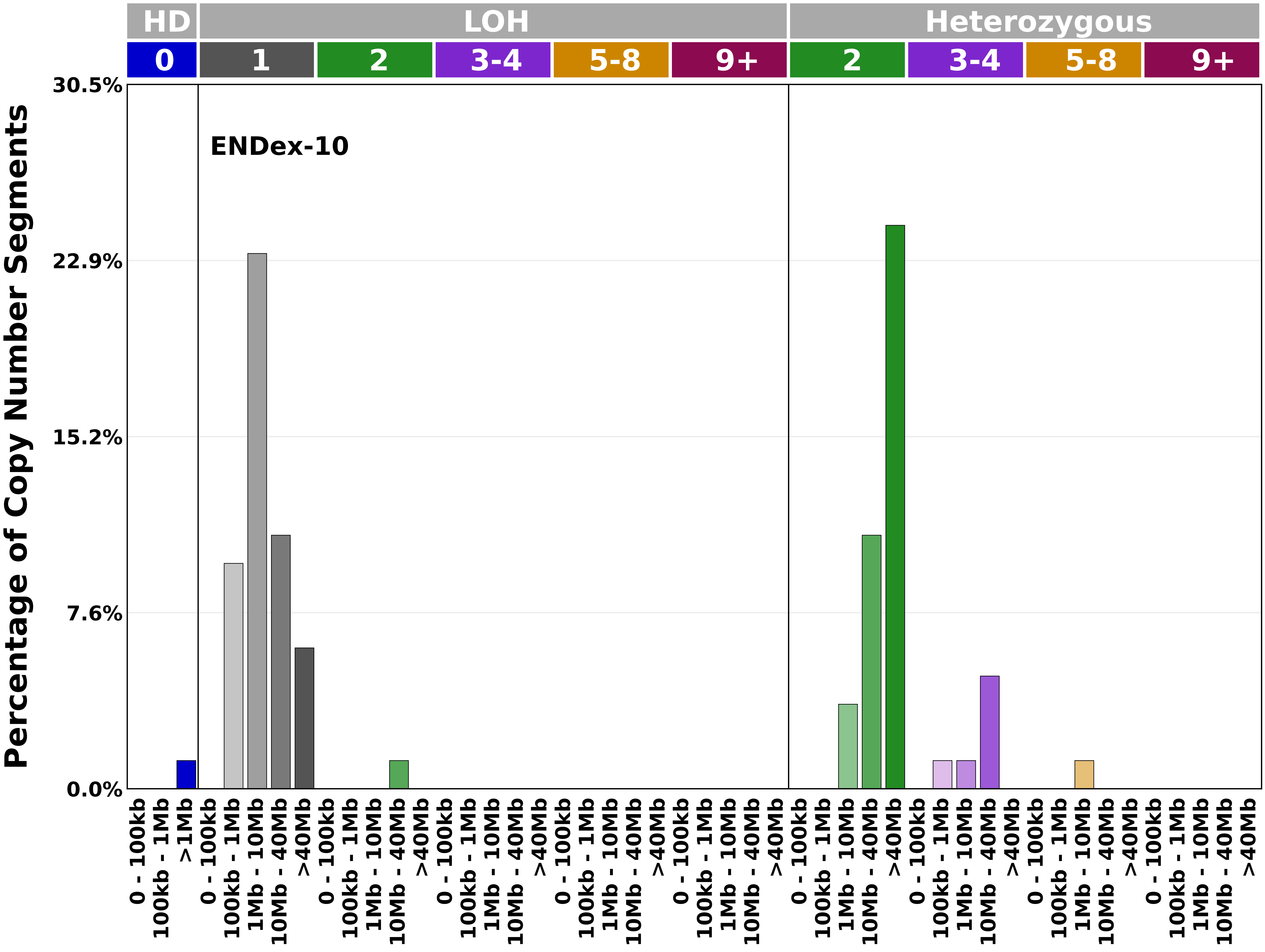

HD
LOH
Heterozygous
0
1
2
3-4
5-8
9+
2
3-4
5-8
9+
ENDex-10
Percentage of Copy Number Segments
30.5%
22.9%
15.2%
7.6%
0.0%
0 - 100kb
100kb - 1Mb
>1Mb
1Mb - 10Mb
10Mb - 40Mb
>40Mb

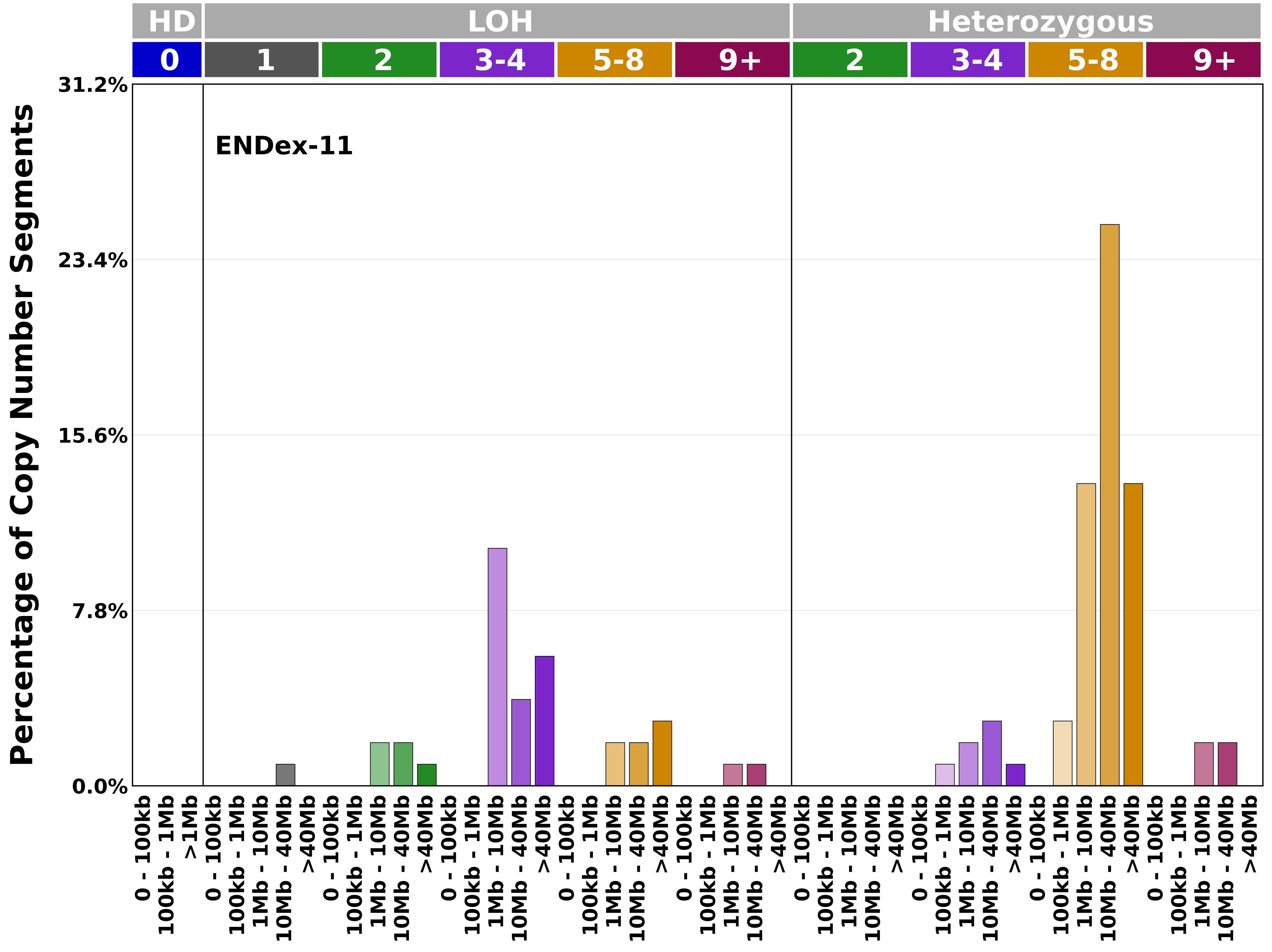
HD
LOH
Heterozygous
0
1
2
3-4
5-8
9+
2
3-4
5-8
9+
ENDex-11
Percentage of Copy Number Segments
31.2%
23.4%
15.6%
7.8%
0.0%
0 - 100kb
100kb - 1Mb
>1Mb
1Mb - 10Mb
10Mb - 40Mb
>40Mb

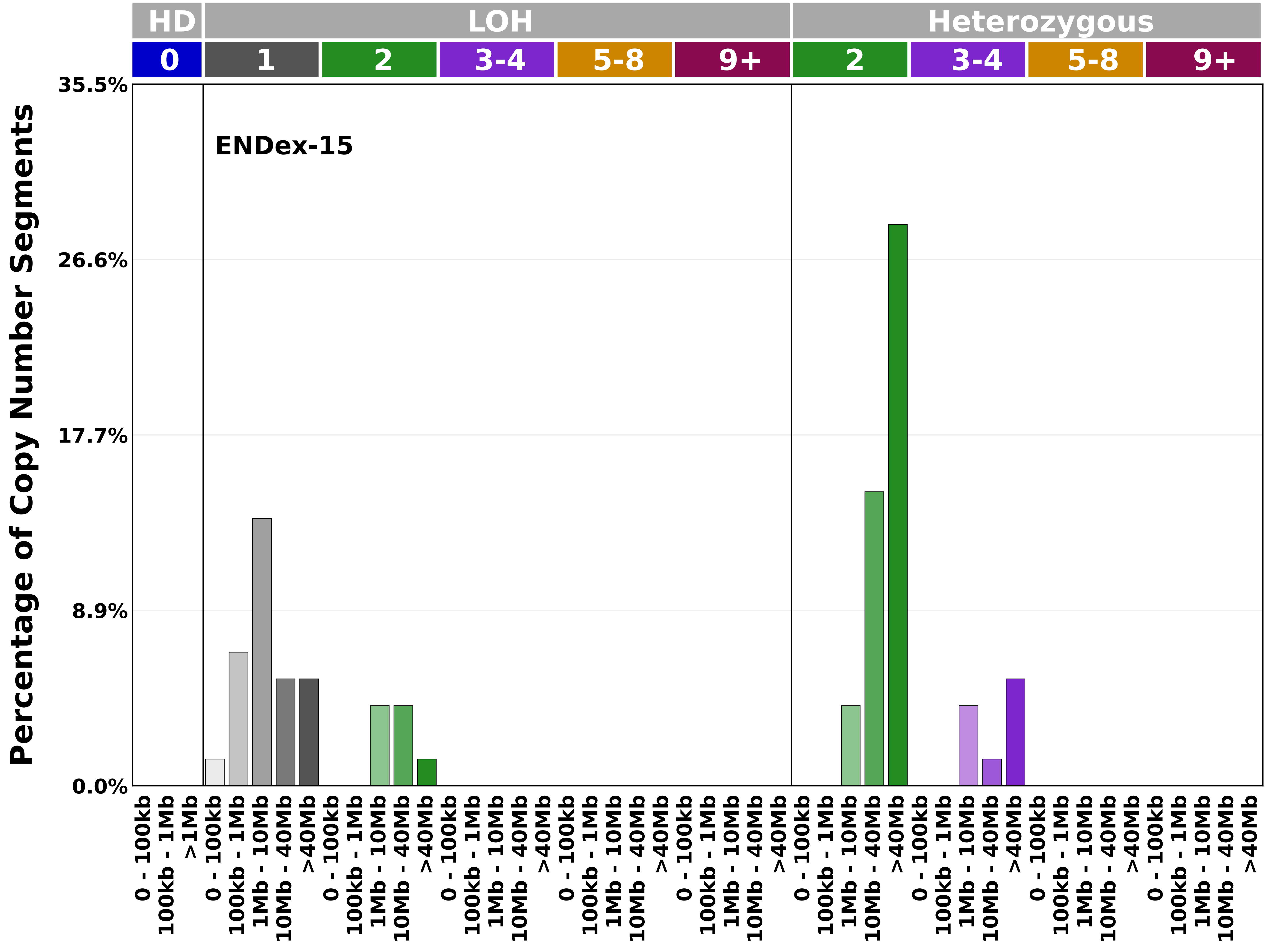

HD
LOH
Heterozygous
0
1
2
3-4
5-8
9+
2
3-4
5-8
9+
ENDex-15
Percentage of Copy Number Segments
35.5%
26.6%
17.7%
8.9%
0.0%
0 - 100kb
100kb - 1Mb
>1Mb
1Mb - 10Mb
10Mb - 40Mb
>40Mb

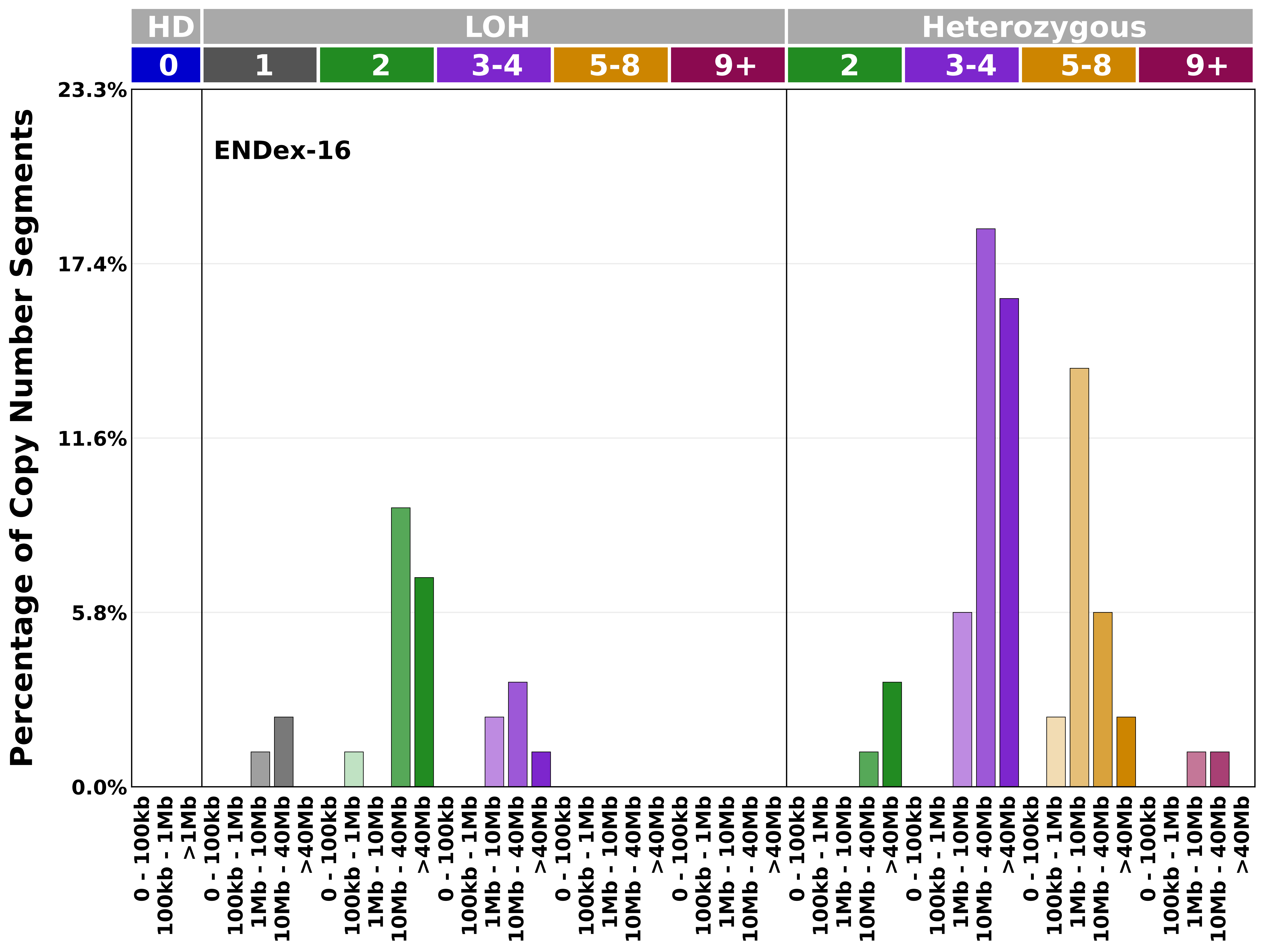

HD
LOH
Heterozygous
0
1
2
3-4
5-8
9+
2
3-4
5-8
9+
ENDex-16
Percentage of Copy Number Segments
23.3%
17.4%
11.6%
5.8%
0.0%
0 - 100kb
100kb - 1Mb
>1Mb
1Mb - 10Mb
10Mb - 40Mb
>40Mb

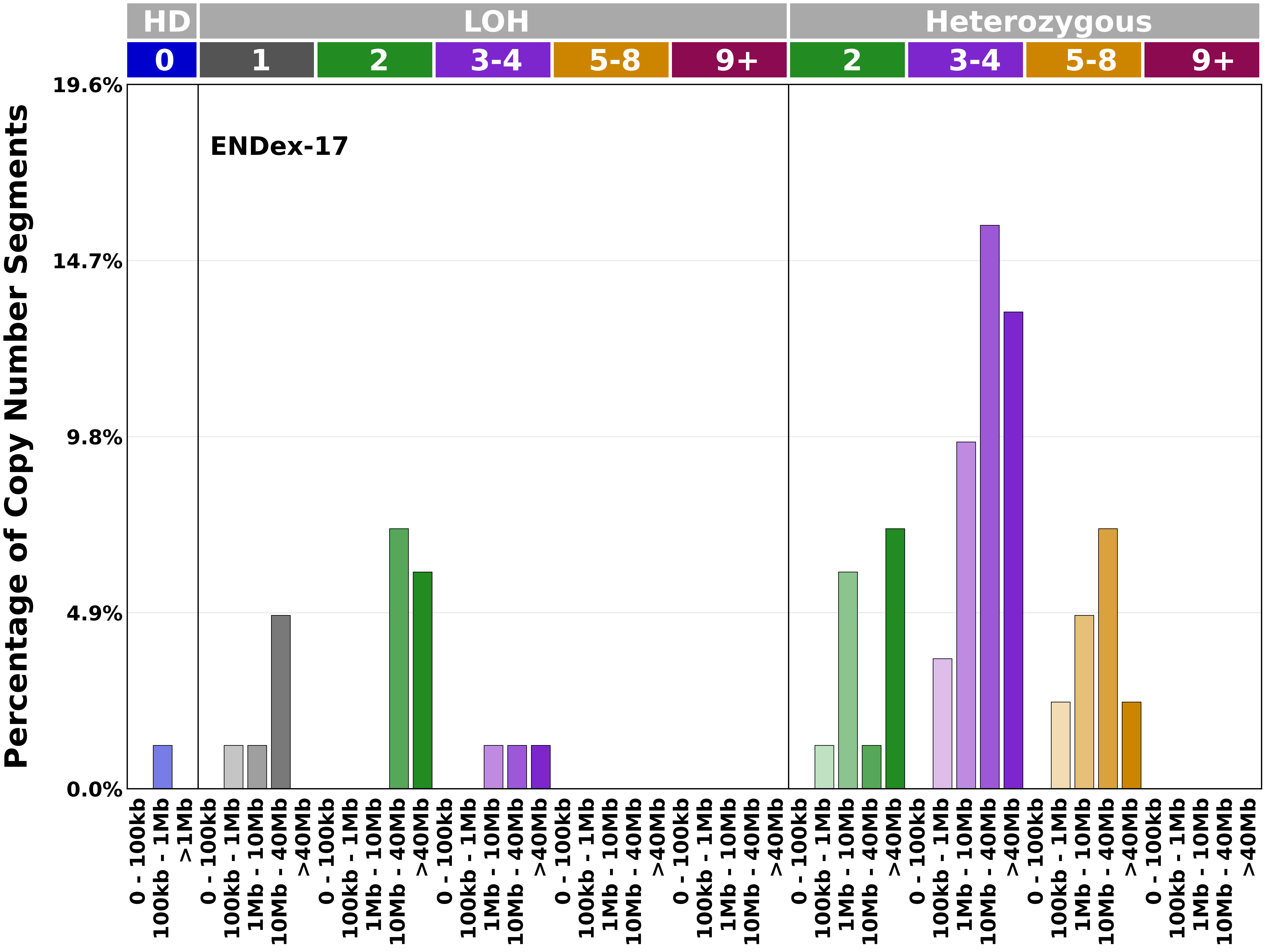

HD
LOH
Heterozygous
0
1
2
3-4
5-8
9+
2
3-4
5-8
9+
ENDex-17
Percentage of Copy Number Segments
19.6%
14.7%
9.8%
4.9%
0.0%
0 - 100kb
100kb - 1Mb
>1Mb
1Mb - 10Mb
10Mb - 40Mb
>40Mb

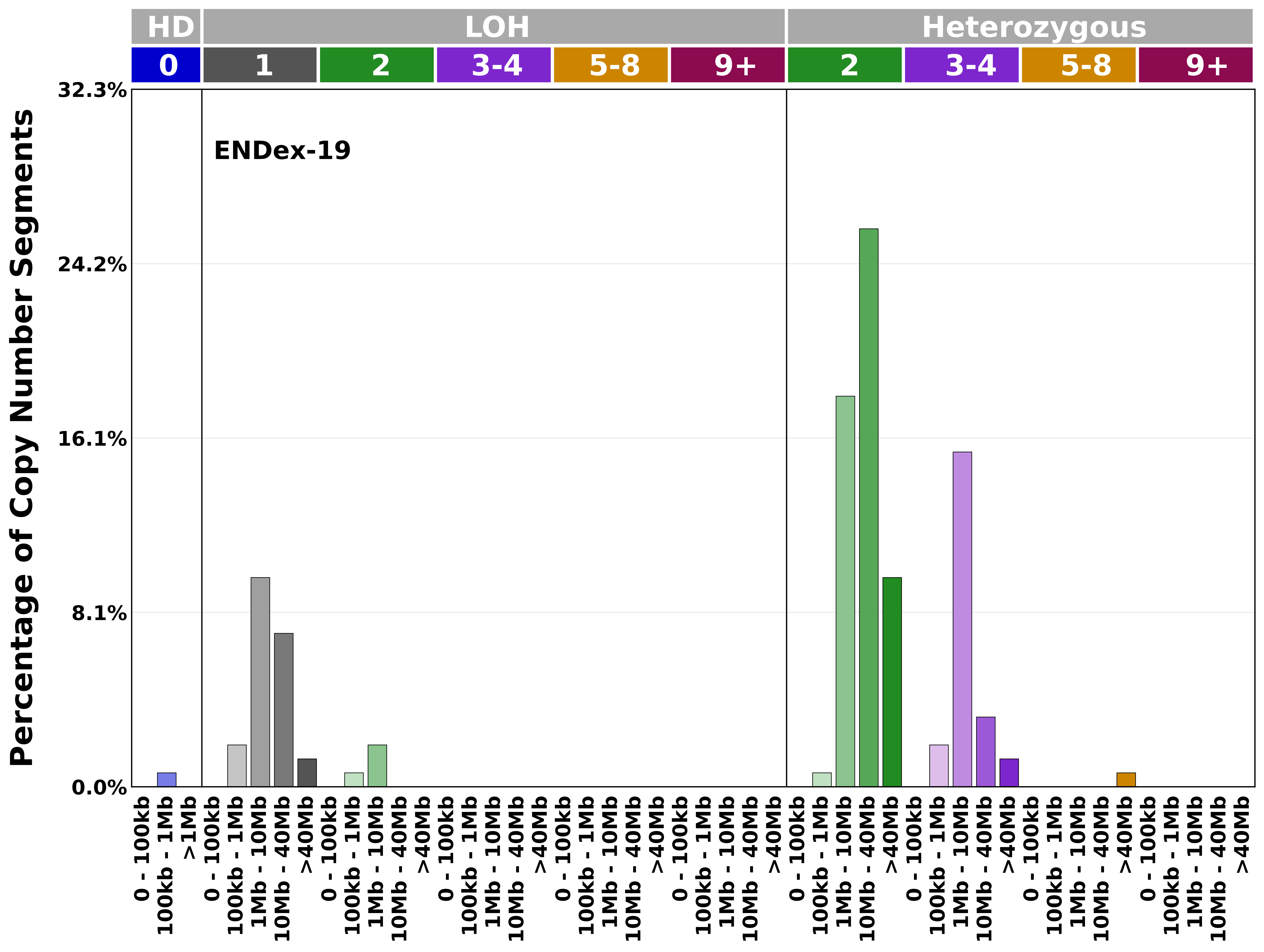
HD
LOH
Heterozygous
0
1
2
3-4
5-8
9+
2
3-4
5-8
9+
Percentage of Copy Number Segments
32.3%
24.2%
16.1%
8.1%
0.0%
ENDex-19
0 - 100kb
100kb - 1Mb
1Mb - 10Mb
10Mb - 40Mb
>40Mb
>1Mb

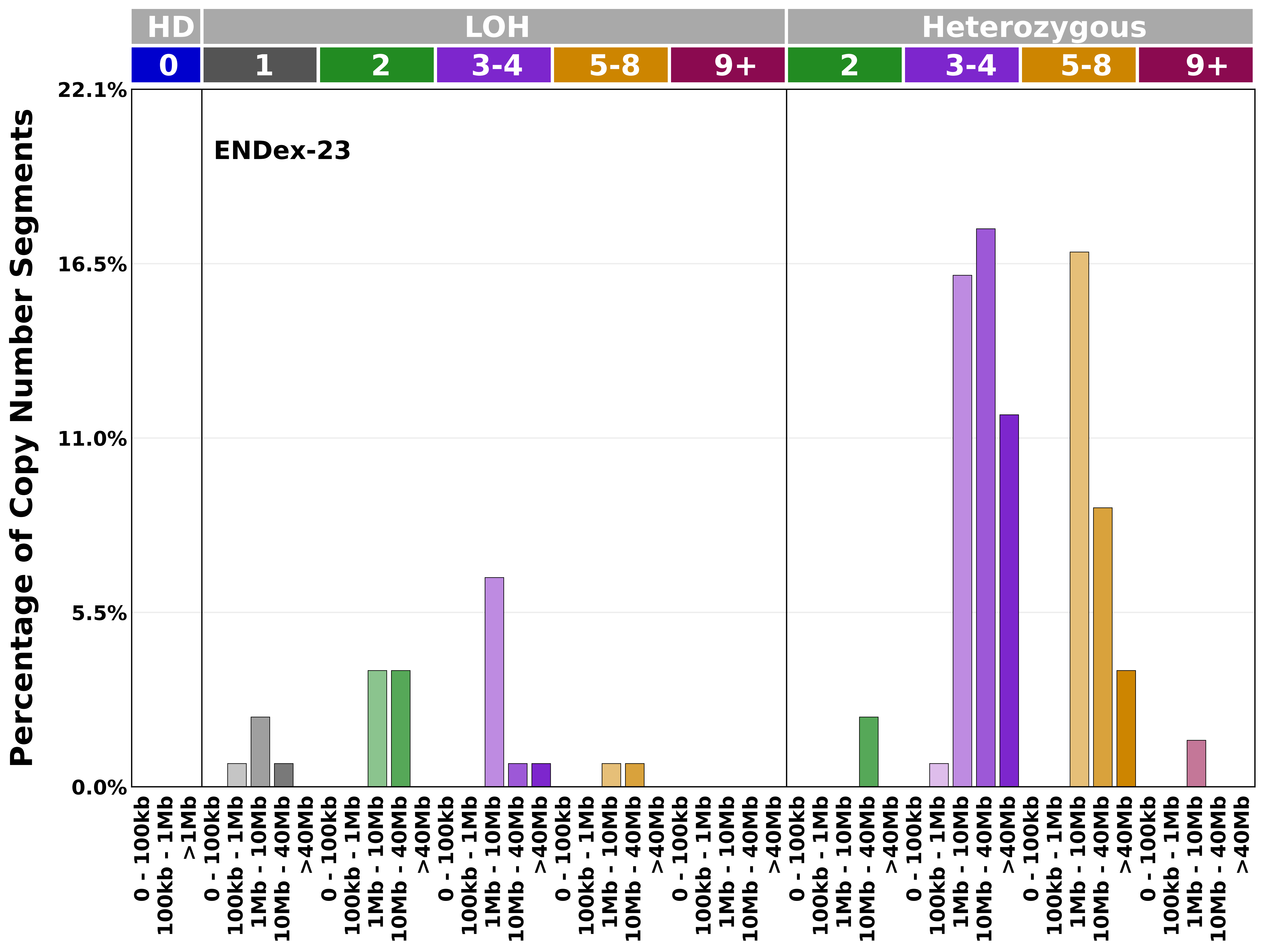
HD
LOH
Heterozygous
0
1
2
3-4
5-8
9+
2
3-4
5-8
9+
ENDex-23
Percentage of Copy Number Segments
22.1%
16.5%
11.0%
5.5%
0.0%
0 - 100kb
100kb - 1Mb
>1Mb
1Mb - 10Mb
10Mb - 40Mb
>40Mb

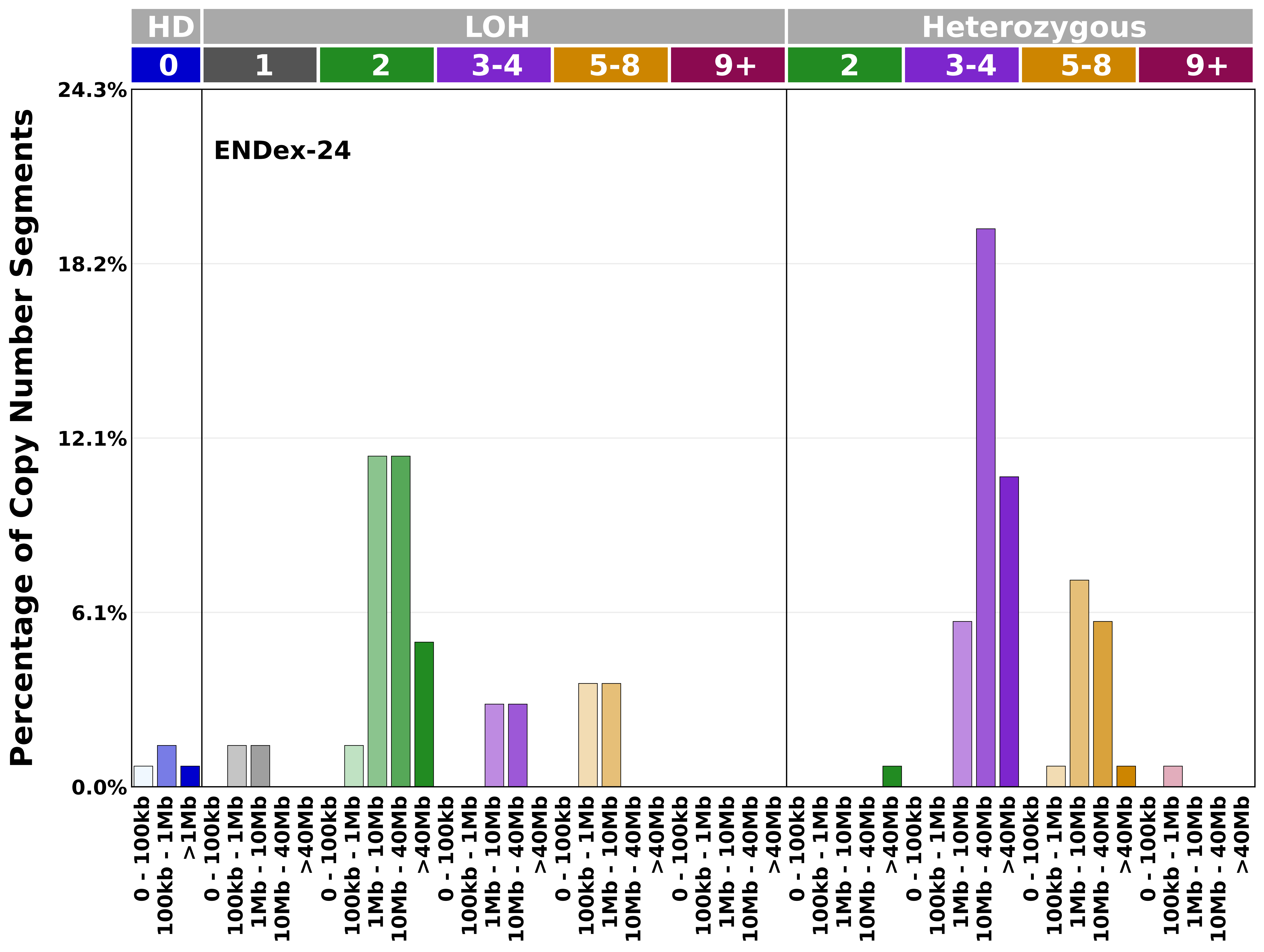
HD
LOH
Heterozygous
0
1
2
3-4
5-8
9+
2
3-4
5-8
9+
ENDex-24
Percentage of Copy Number Segments
24.3%
18.2%
12.1%
6.1%
0.0%
0 - 100kb
100kb - 1Mb
>1Mb
1Mb - 10Mb
10Mb - 40Mb
>40Mb

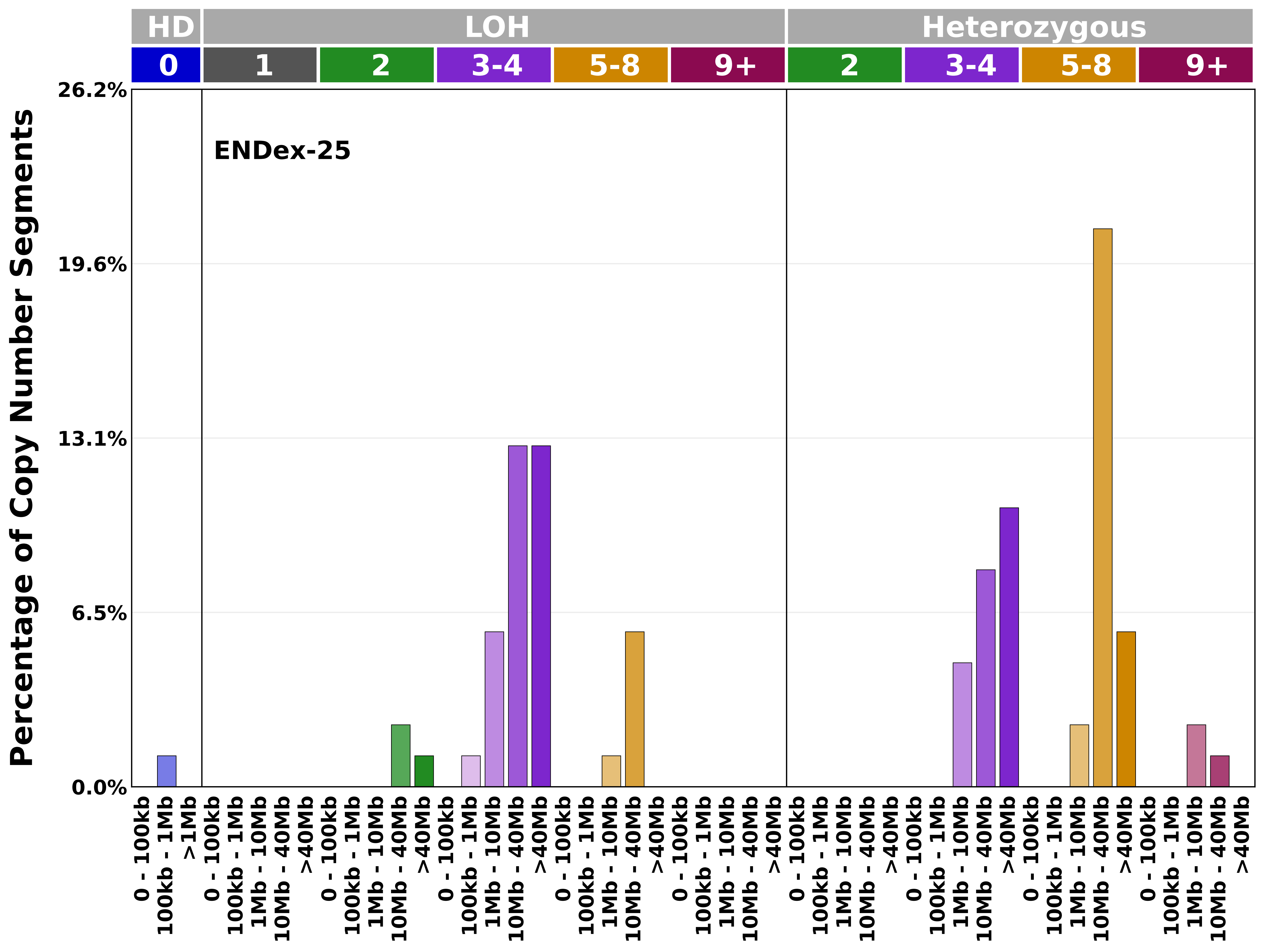

HD
LOH
Heterozygous
0
1
2
3-4
5-8
9+
2
3-4
5-8
9+
ENDex-25
Percentage of Copy Number Segments
26.2%
19.6%
13.1%
6.5%
0.0%
0 - 100kb
100kb - 1Mb
>1Mb
1Mb - 10Mb
10Mb - 40Mb
>40Mb

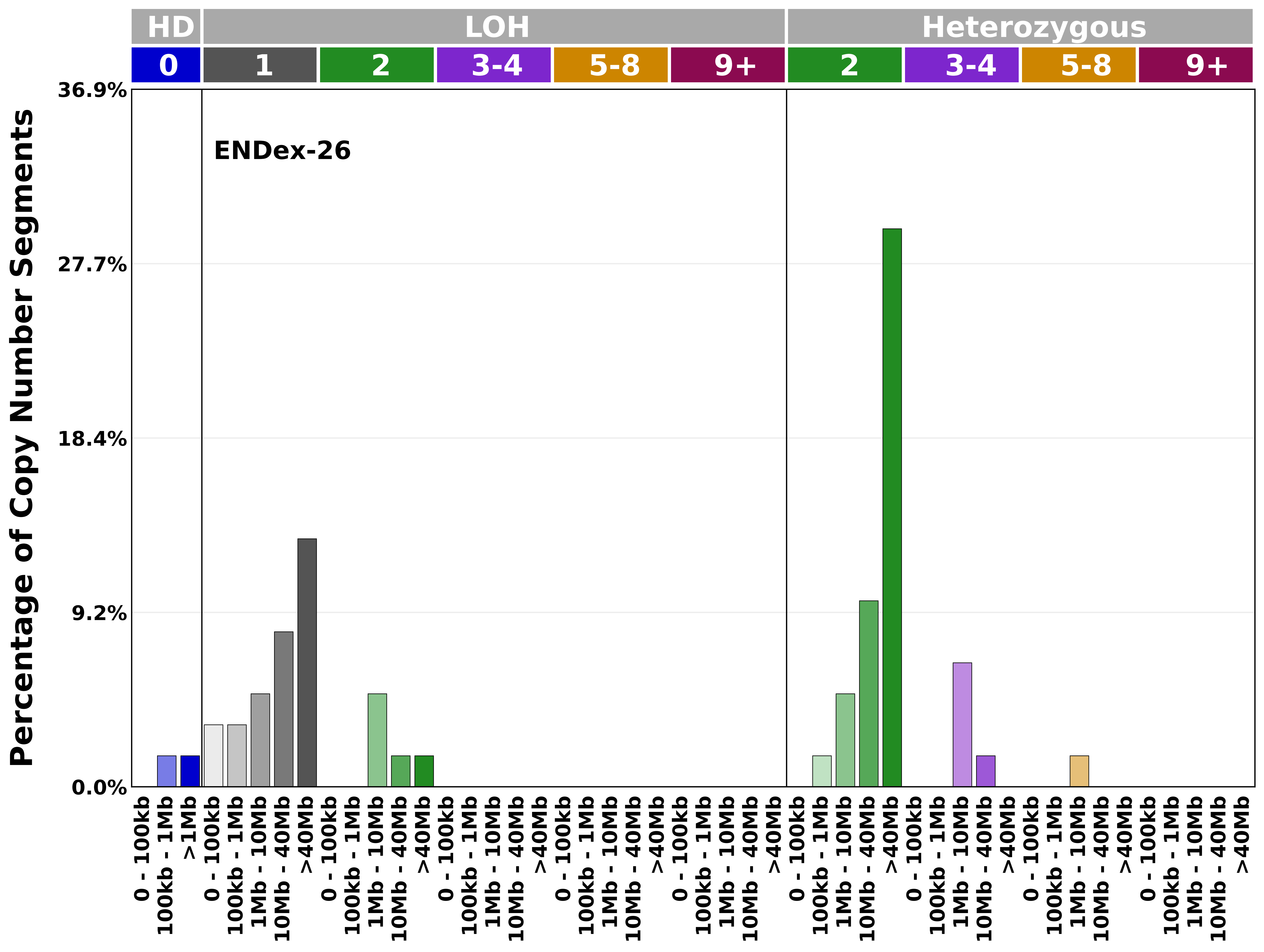
HD
LOH
Heterozygous
0
1
2
3-4
5-8
9+
2
3-4
5-8
9+
ENDex-26
Percentage of Copy Number Segments
36.9%
27.7%
18.4%
9.2%
0.0%
0 - 100kb
100kb - 1Mb
>1Mb
1Mb - 10Mb
10Mb - 40Mb
>40Mb

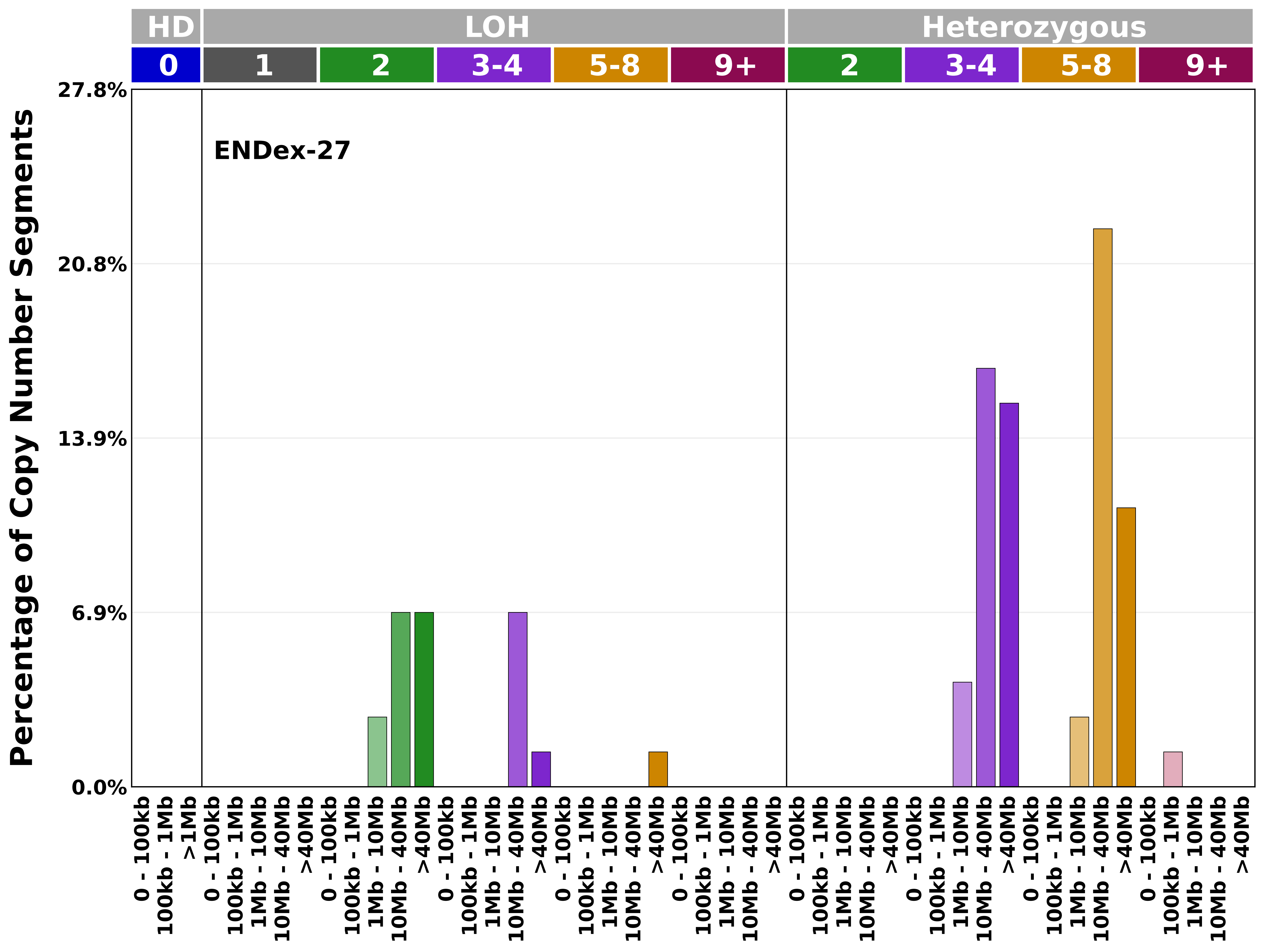
HD
LOH
Heterozygous
0
1
2
3-4
5-8
9+
2
3-4
5-8
9+
ENDex-27
Percentage of Copy Number Segments
27.8%
20.8%
13.9%
6.9%
0.0%
0 - 100kb
100kb - 1Mb
>1Mb
1Mb - 10Mb
10Mb - 40Mb
>40Mb

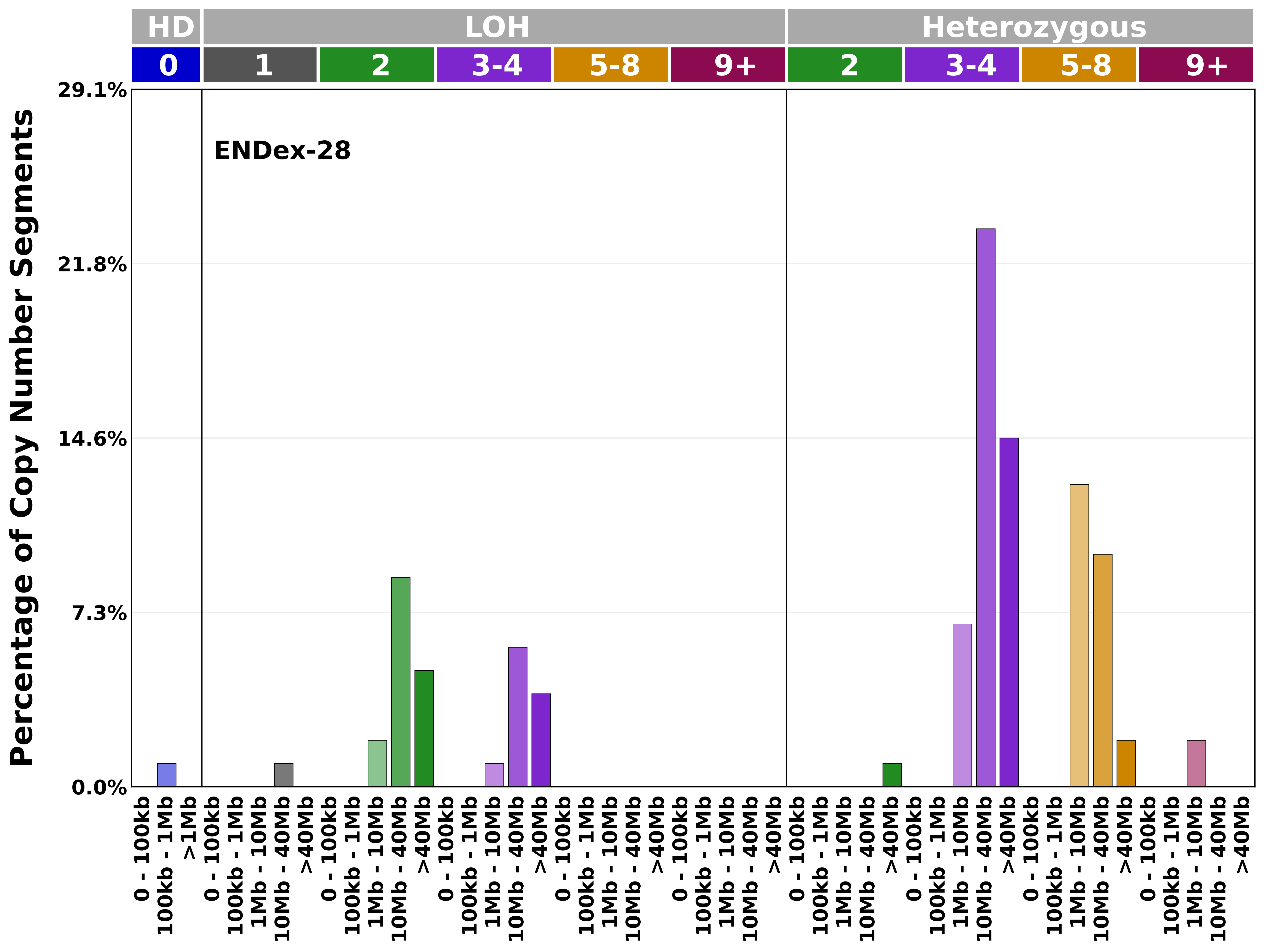

HD
LOH
Heterozygous
0
1
2
3-4
5-8
9+
2
3-4
5-8
9+
ENDex-28
Percentage of Copy Number Segments
29.1%
21.8%
14.6%
7.3%
0.0%
0 - 100kb
100kb - 1Mb
>1Mb
1Mb - 10Mb
10Mb - 40Mb
>40Mb

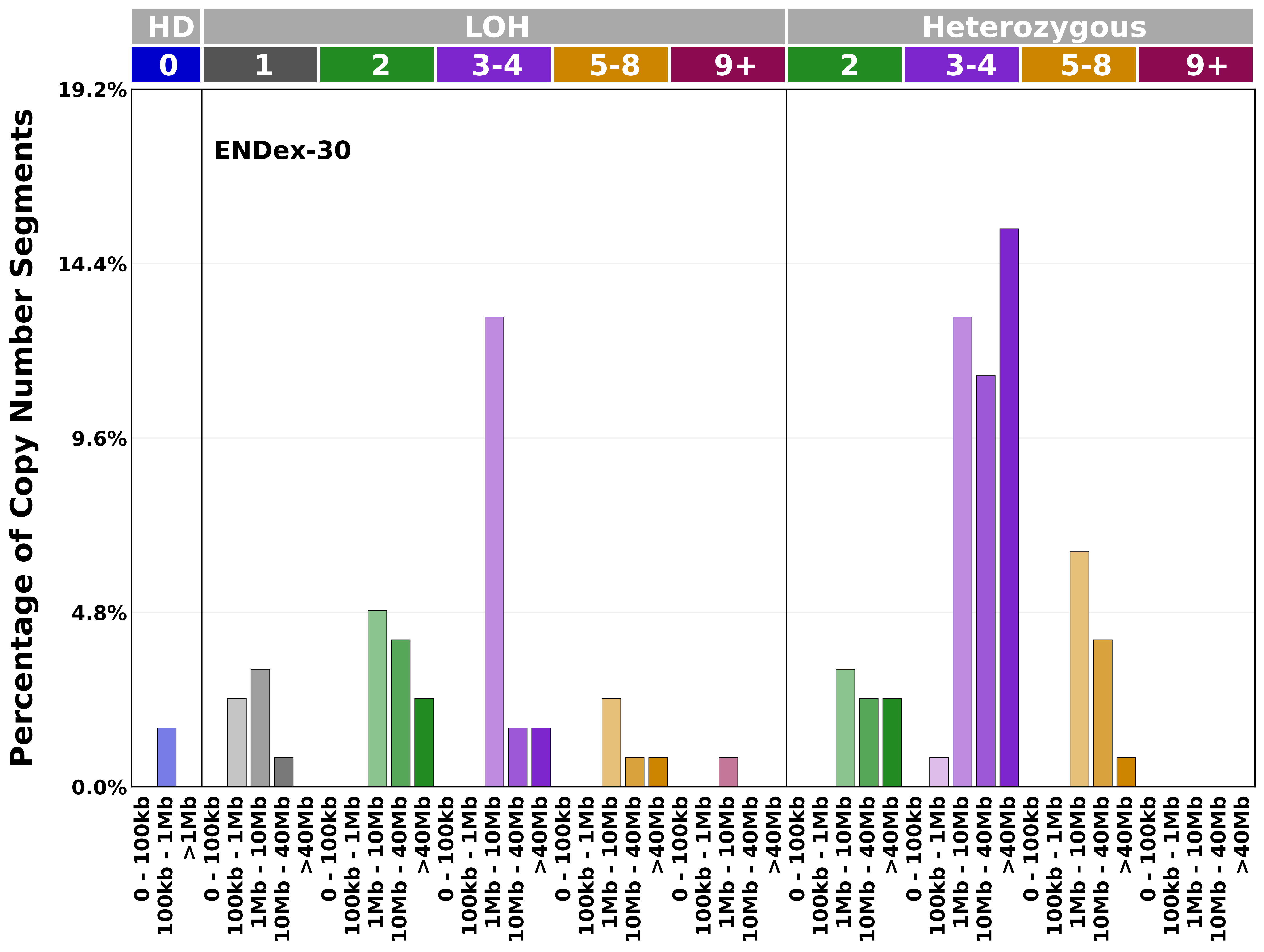
HD
LOH
Heterozygous
0
1
2
3-4
5-8
9+
2
3-4
5-8
9+
ENDex-30
Percentage of Copy Number Segments
19.2%
14.4%
9.6%
4.8%
0.0%
0 - 100kb
100kb - 1Mb
>1Mb
1Mb - 10Mb
10Mb - 40Mb
>40Mb
