# Supplementary figures and images for: Copy number signatures and CCNE1 amplification reveal the involvement of replication stress in high-grade endometrial tumors oncogenesis
Source: Cell Oncol (Dordr). 2024 Apr 2;47(4):1441–57. doi: 10.1007/s13402-024-00942-w (PMC11322381; doi:10.1007/s13402-024-00942-w)

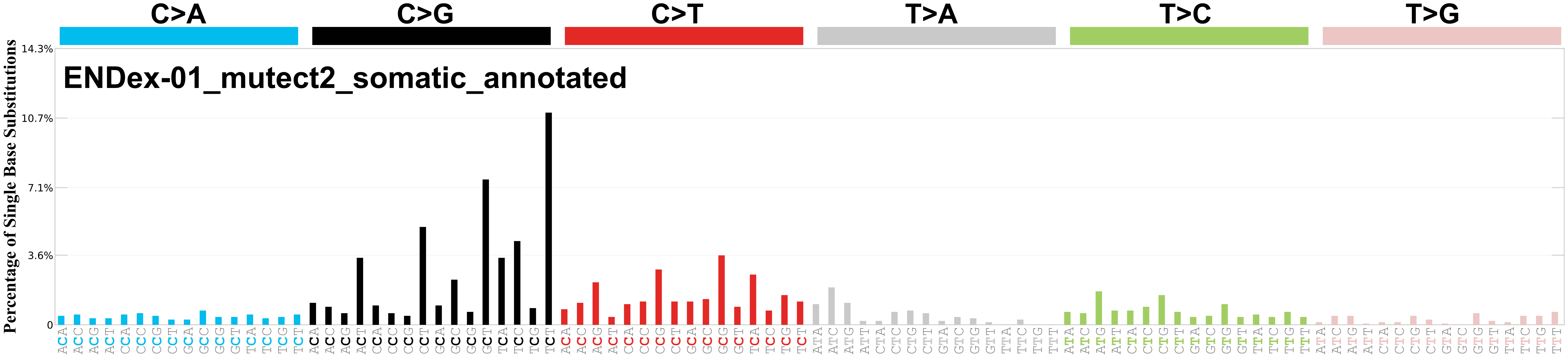

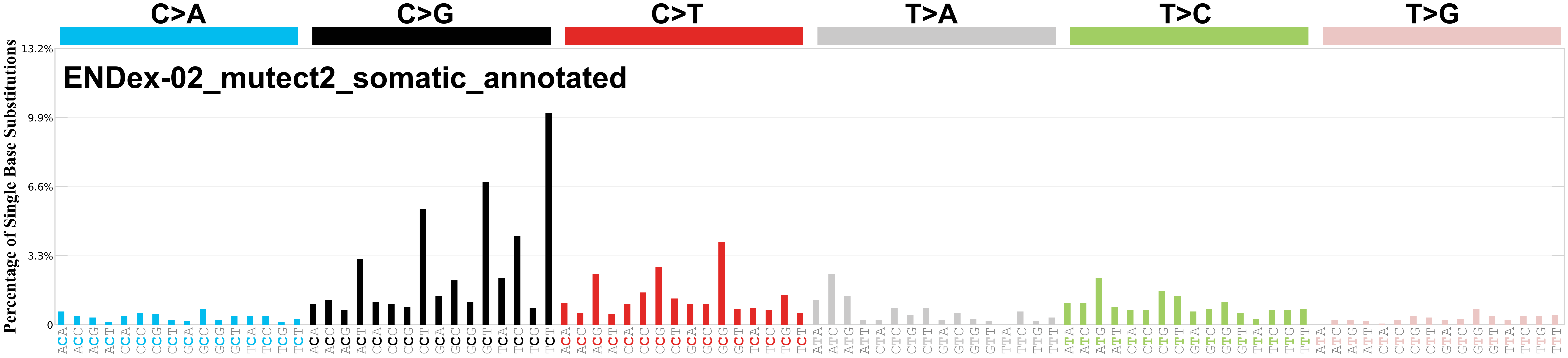

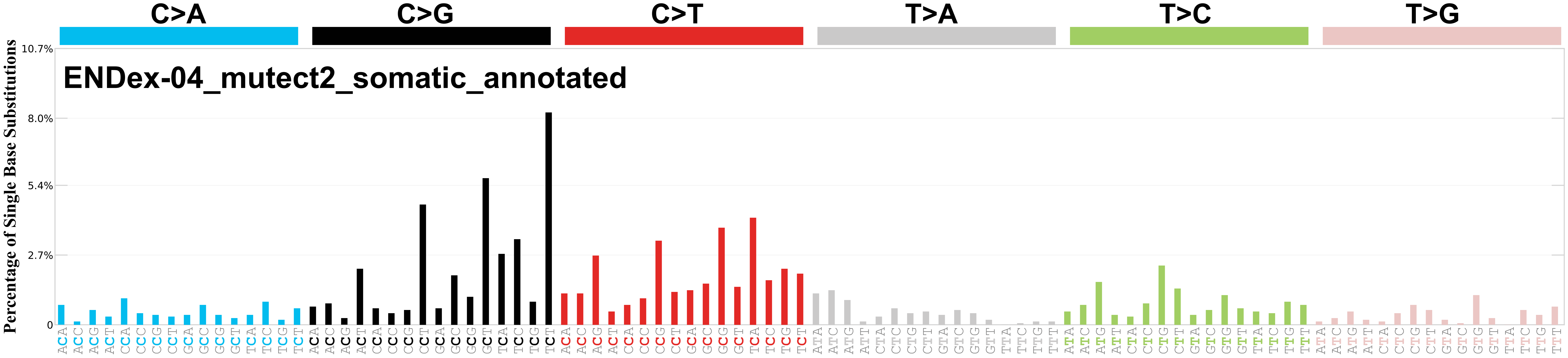

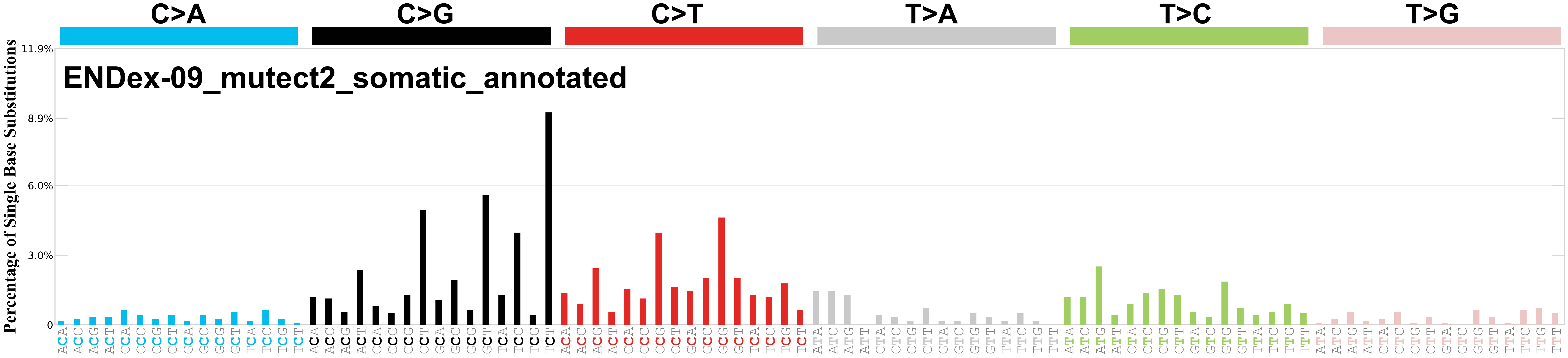

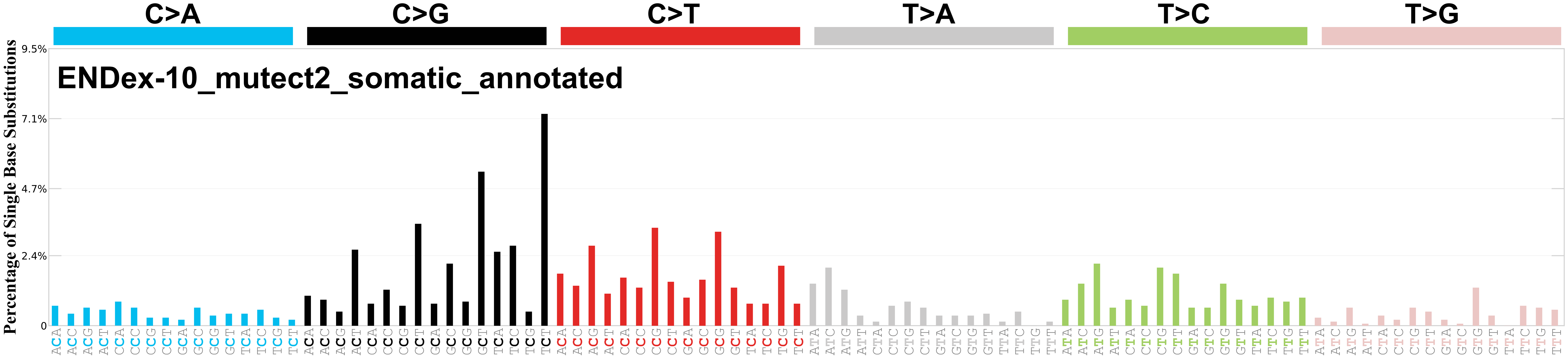

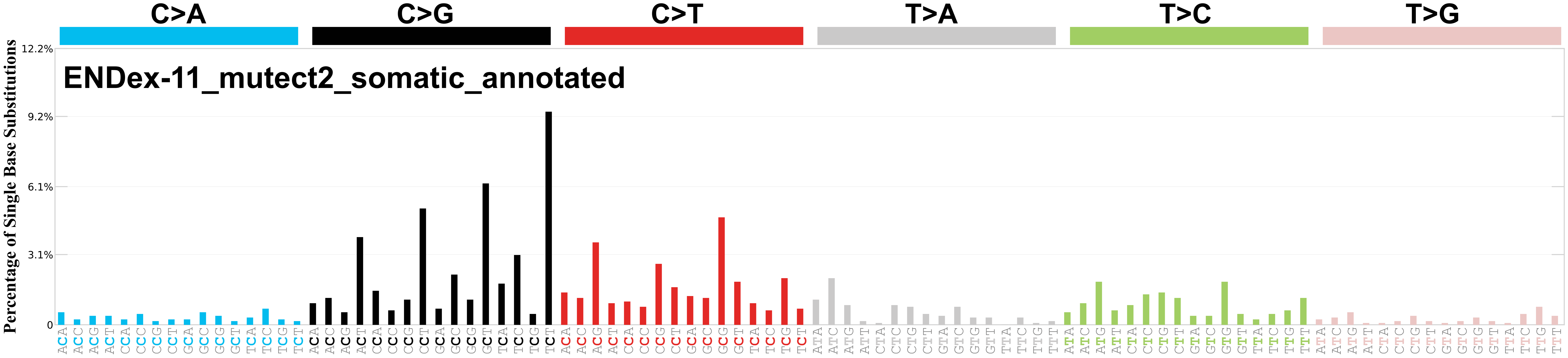

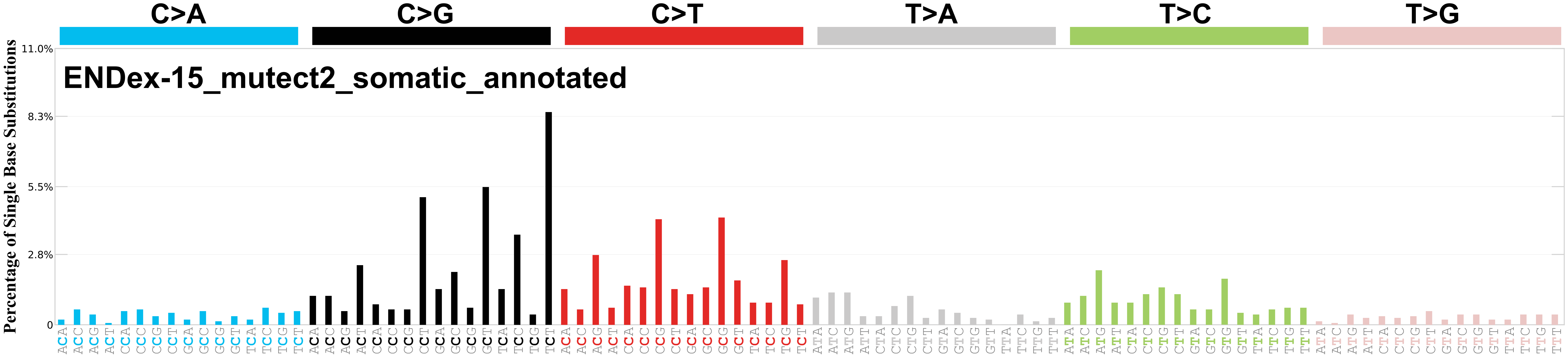

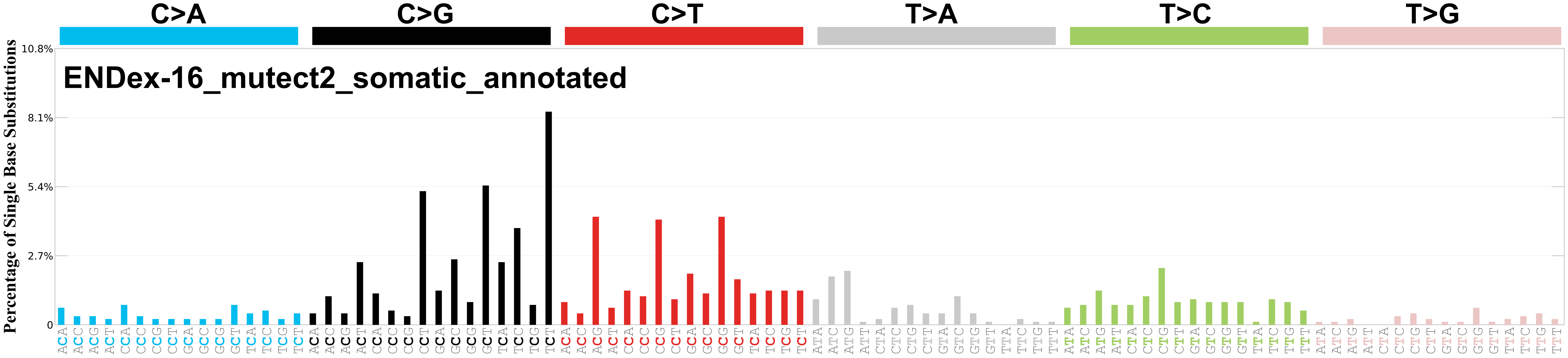

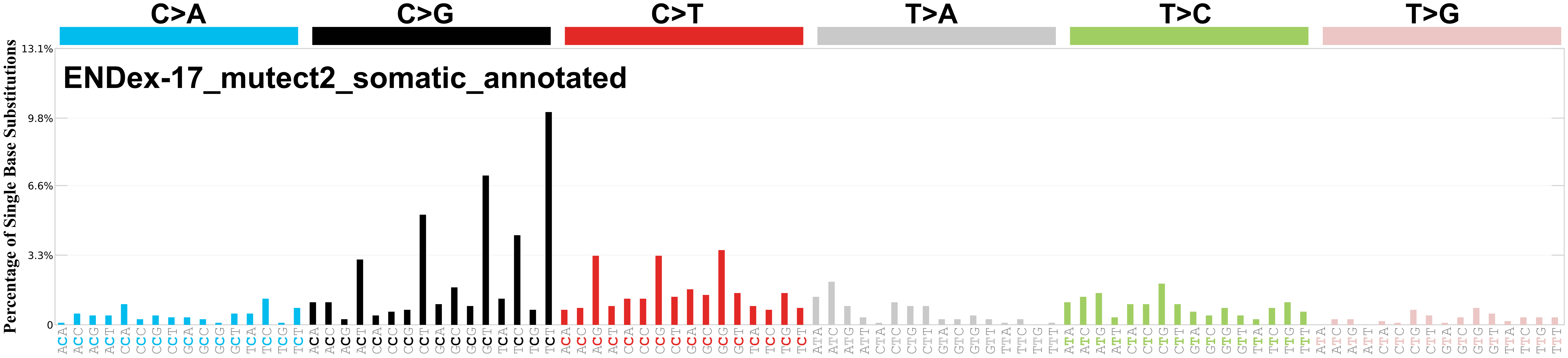

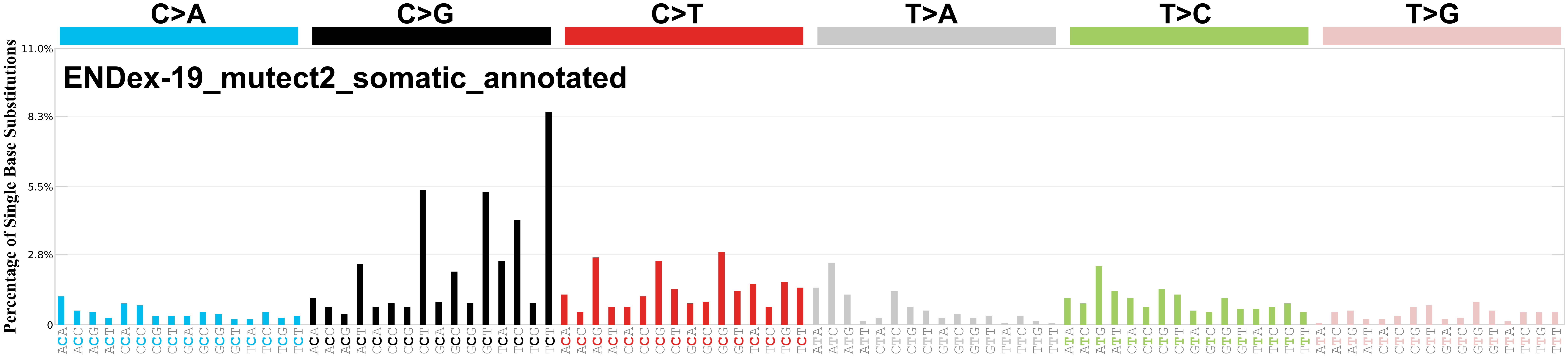

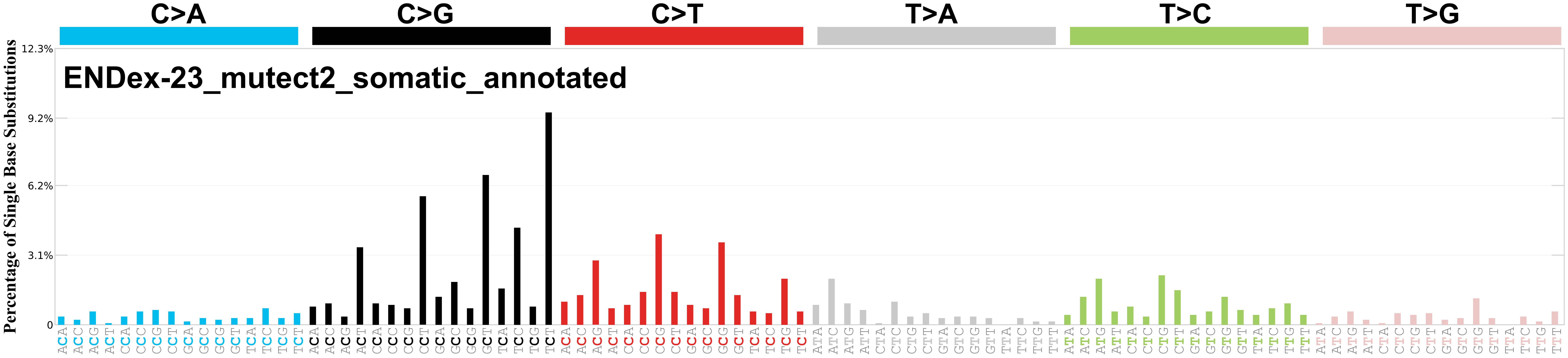

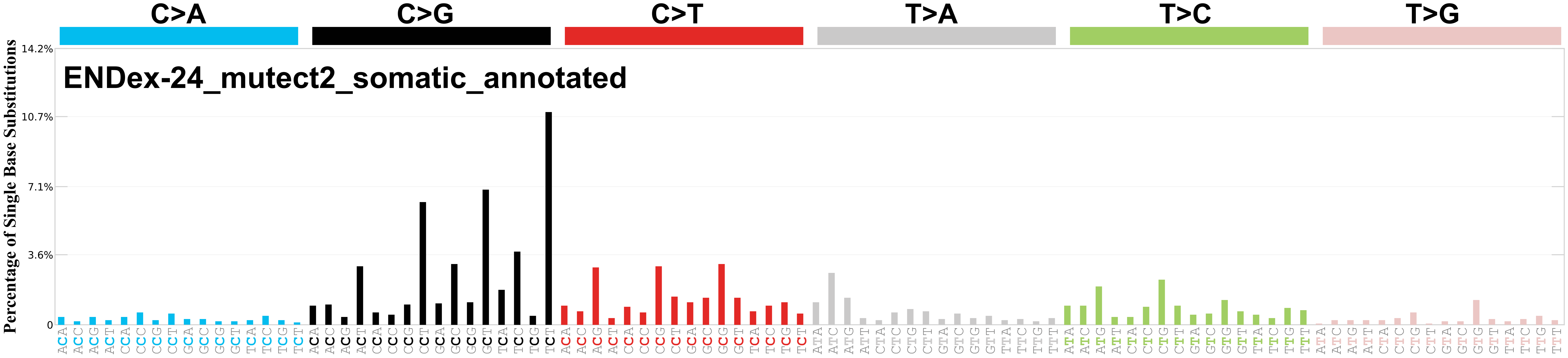

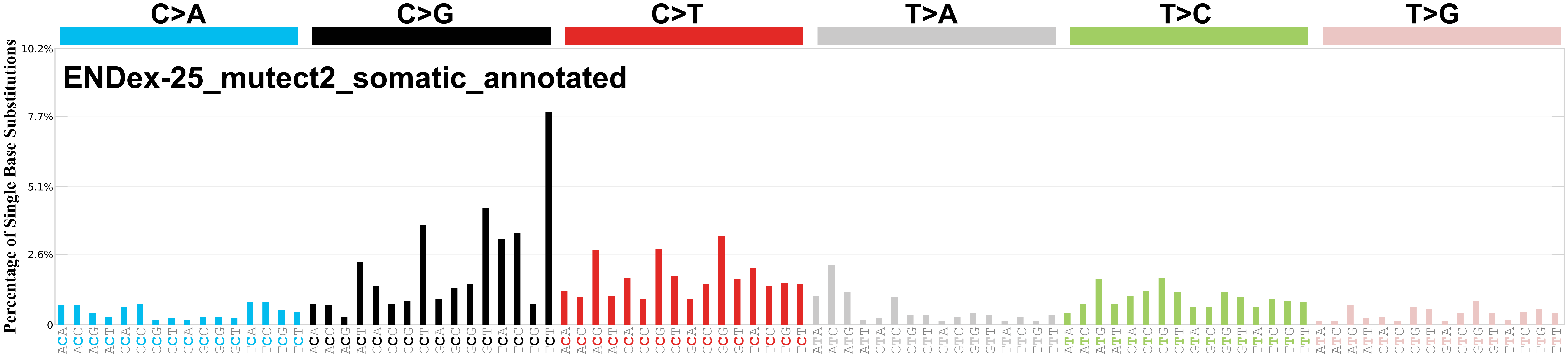

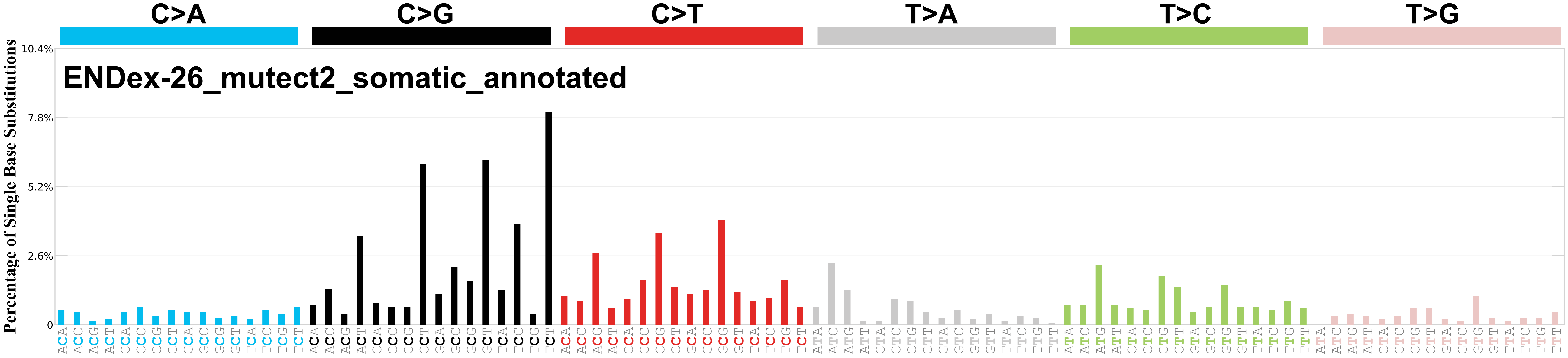

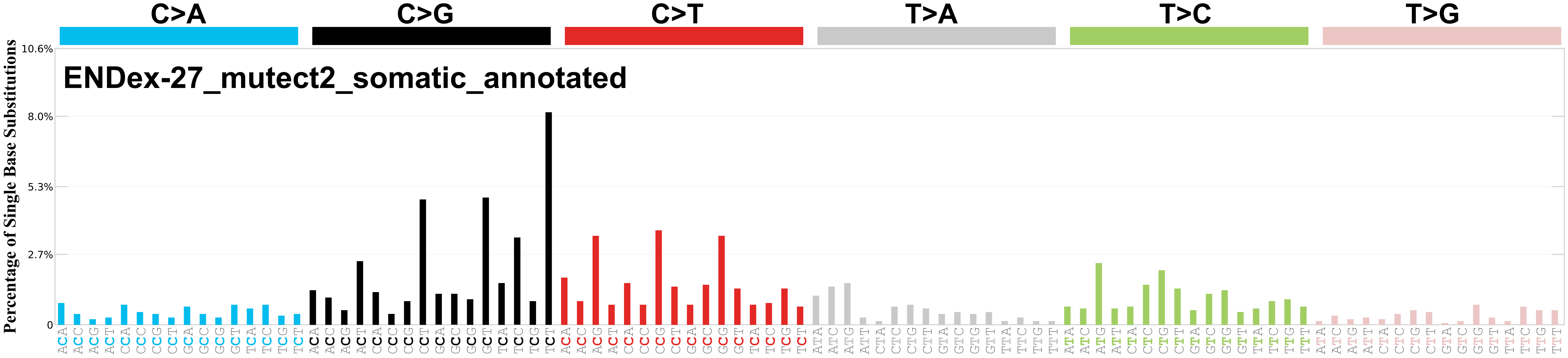

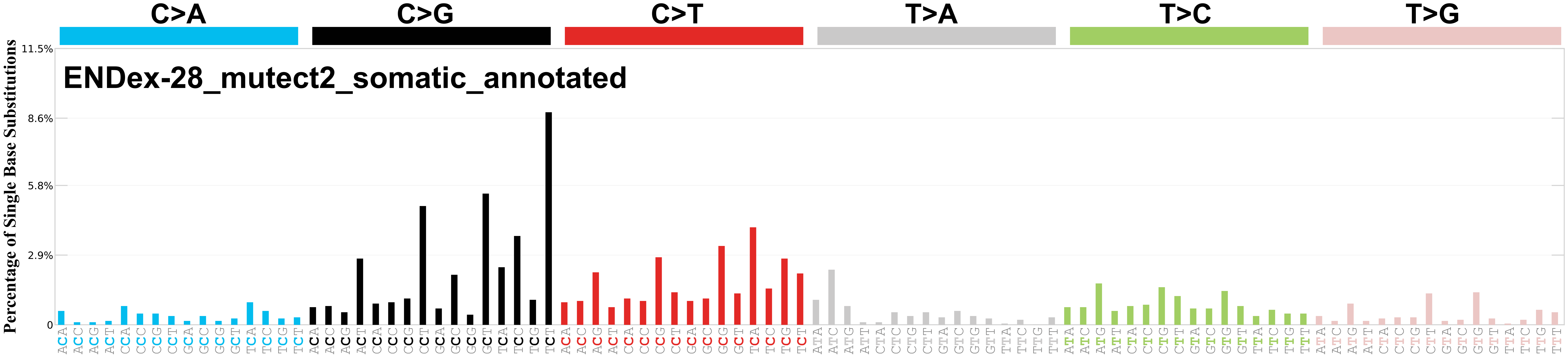

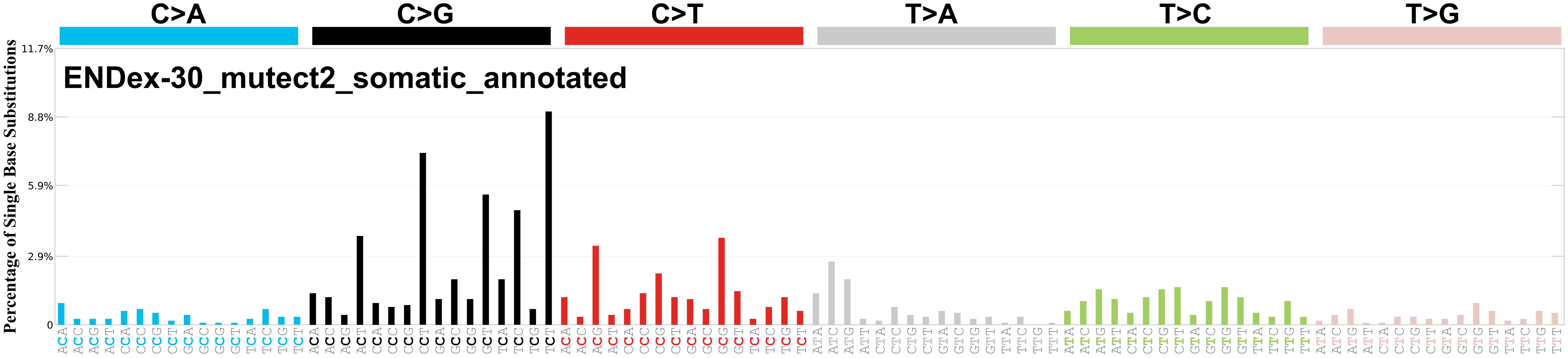

Supplement: Supplementary file 4 — Supplementary Material 4 [file 13402_2024_942_MOESM4_ESM.pdf]

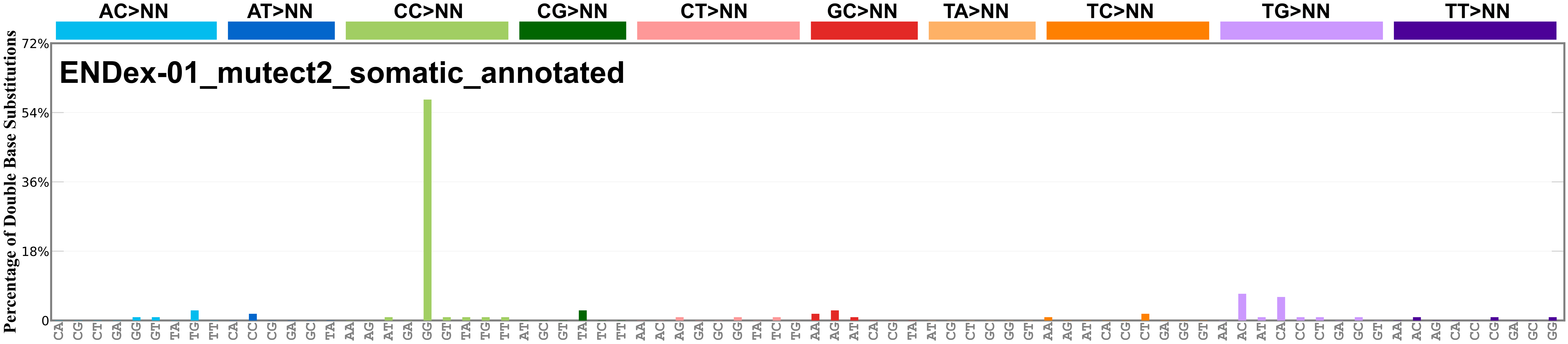

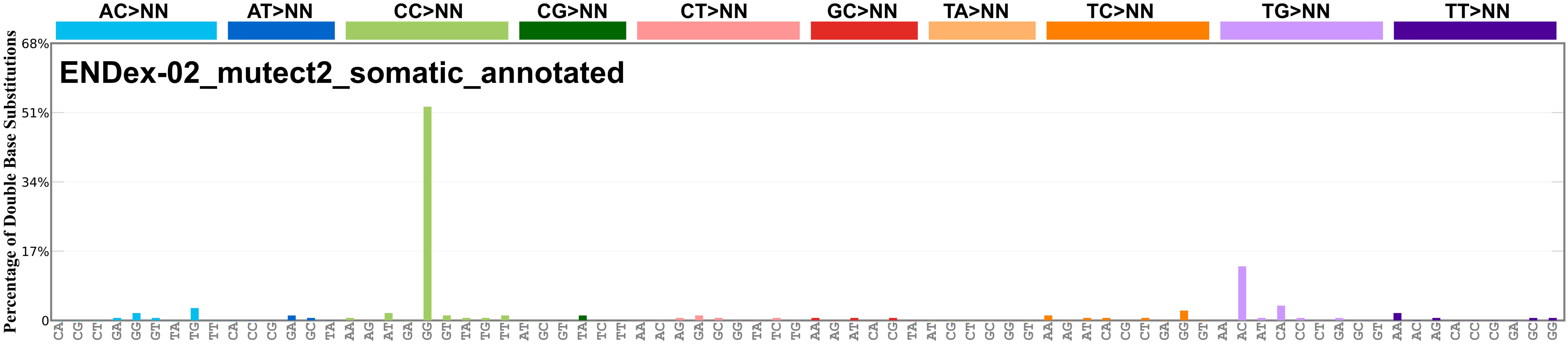

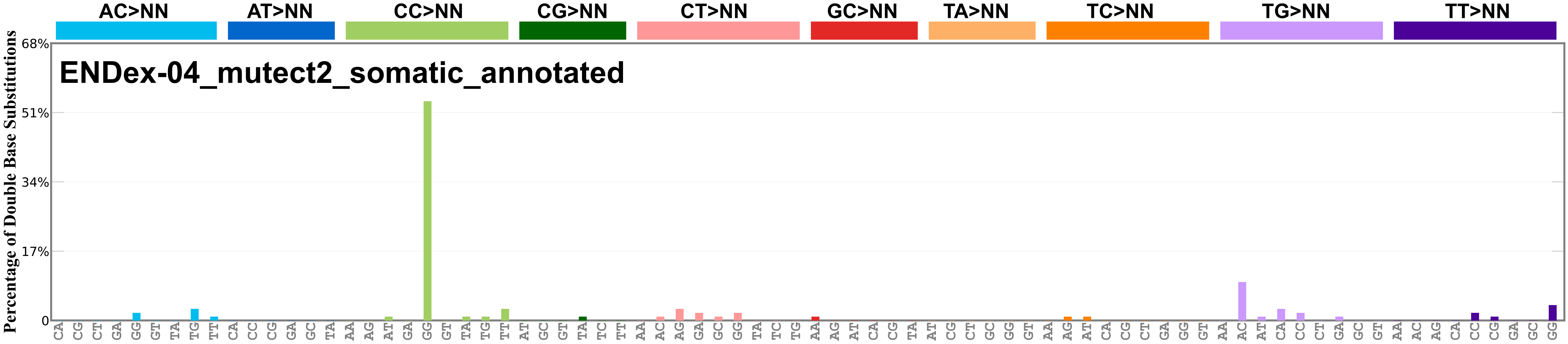

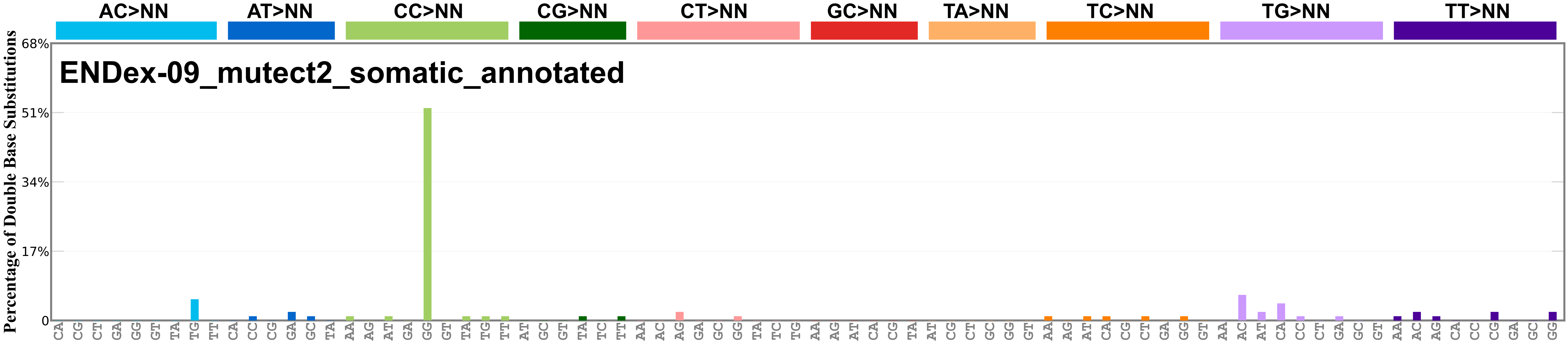

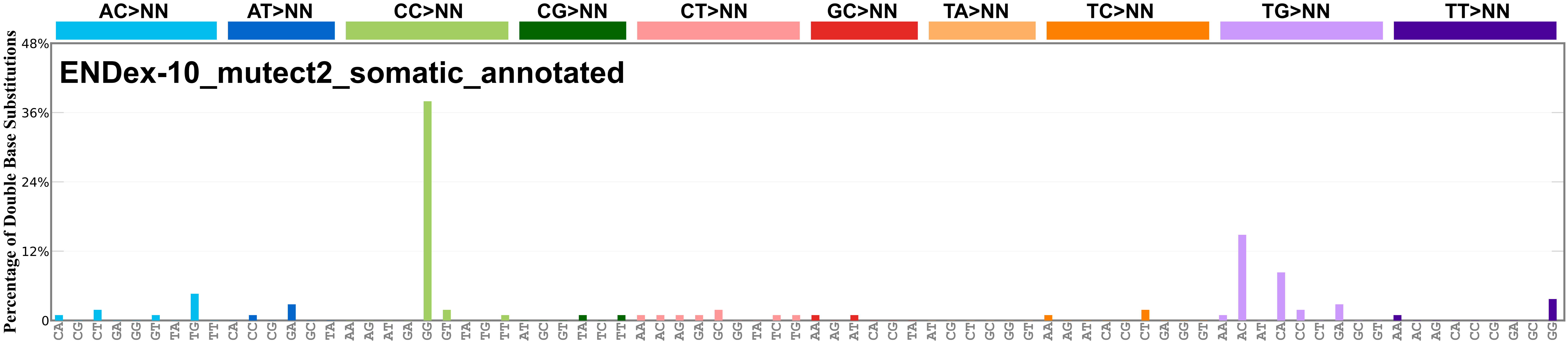

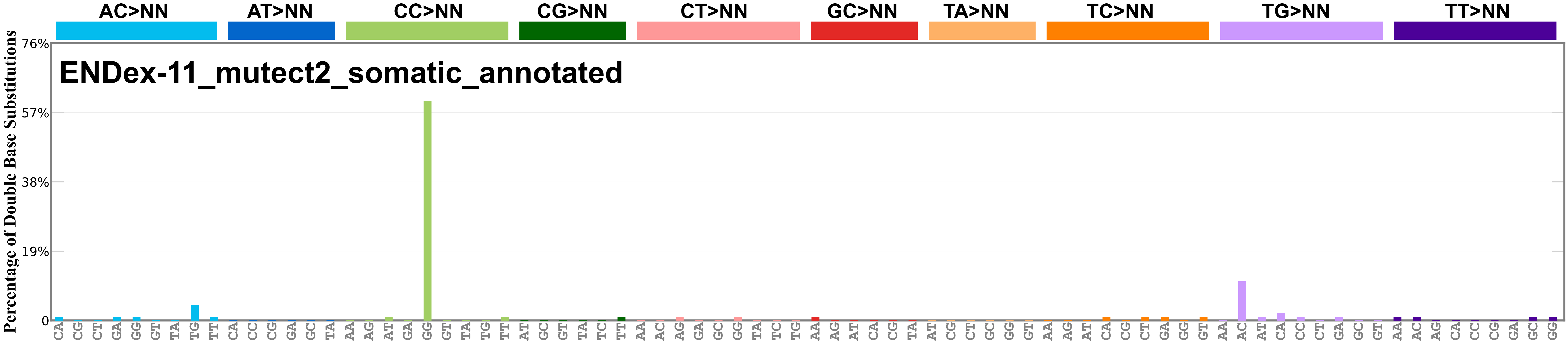

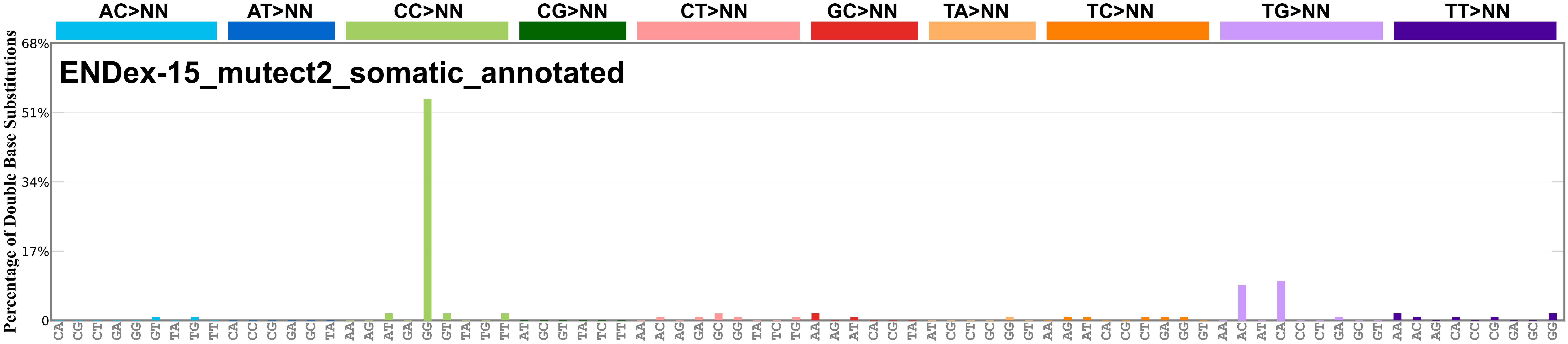

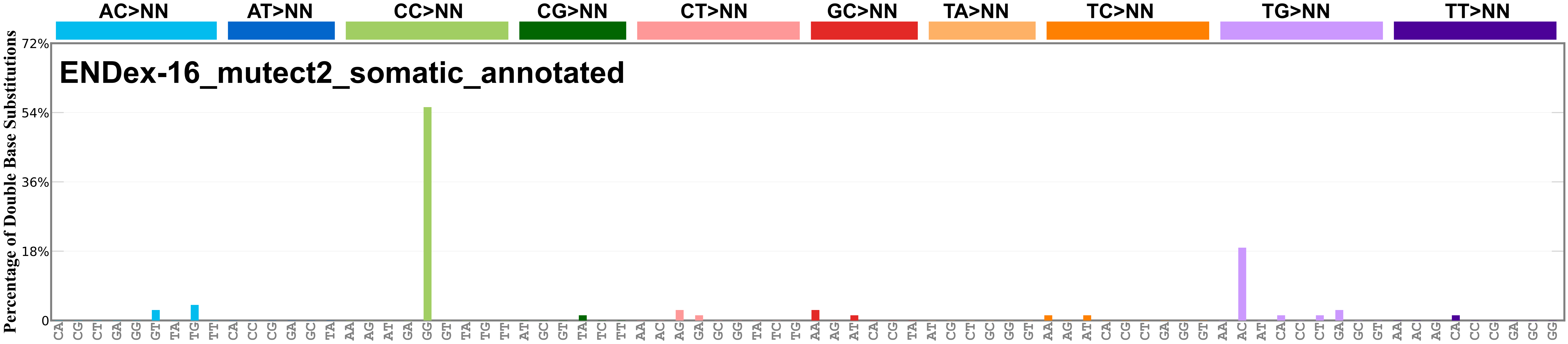

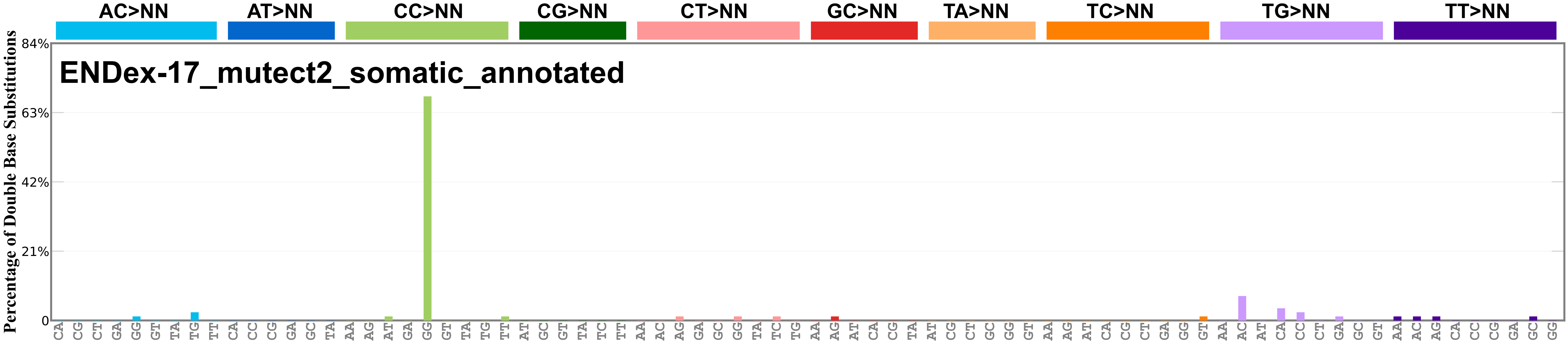

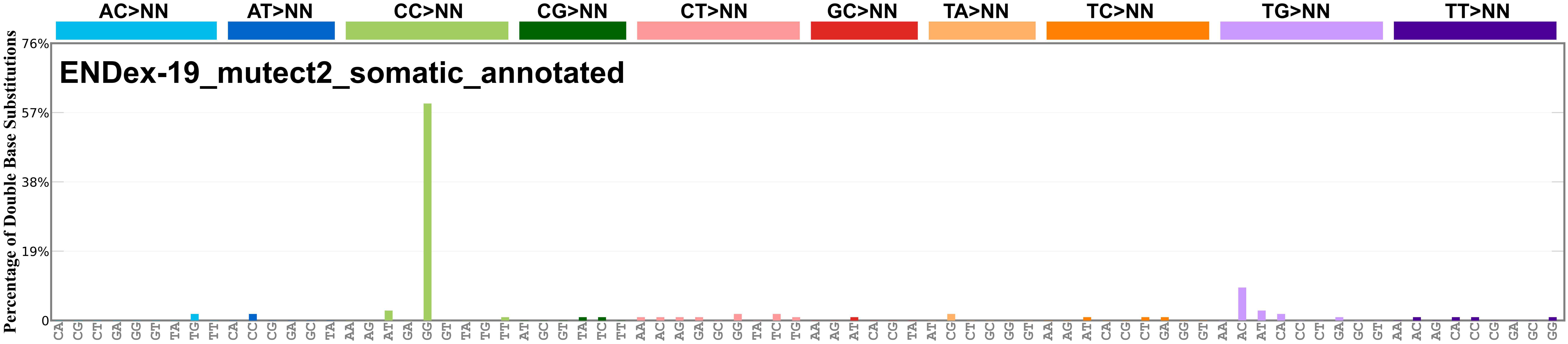

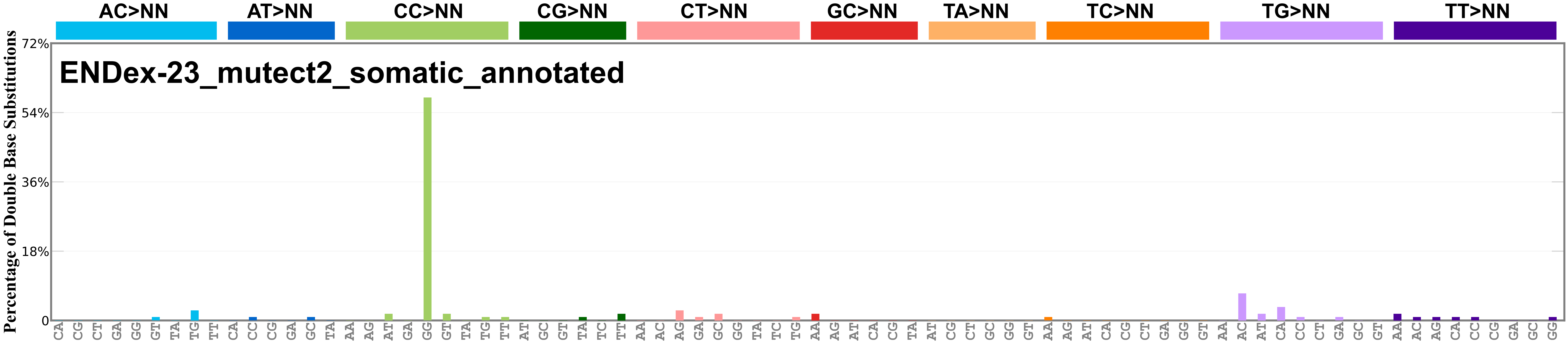

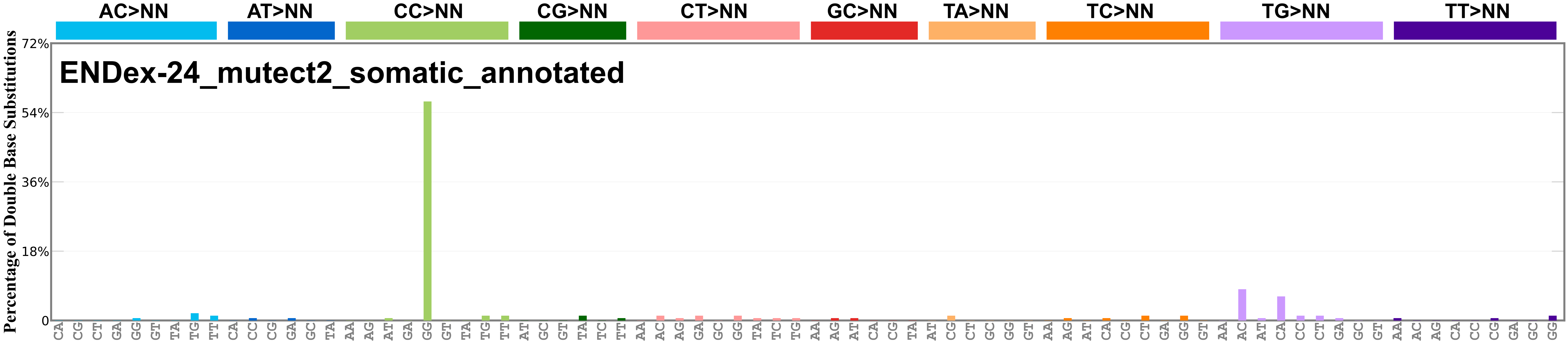

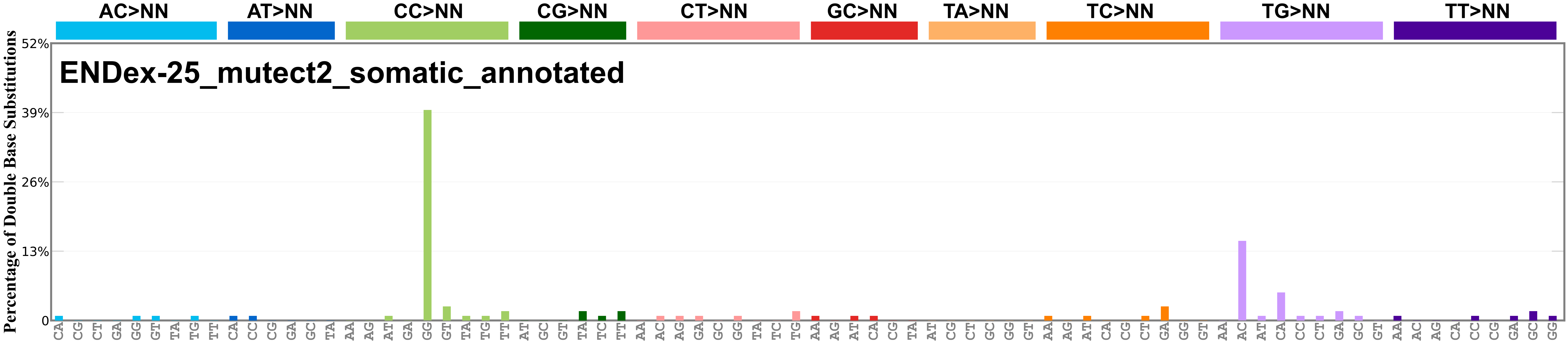

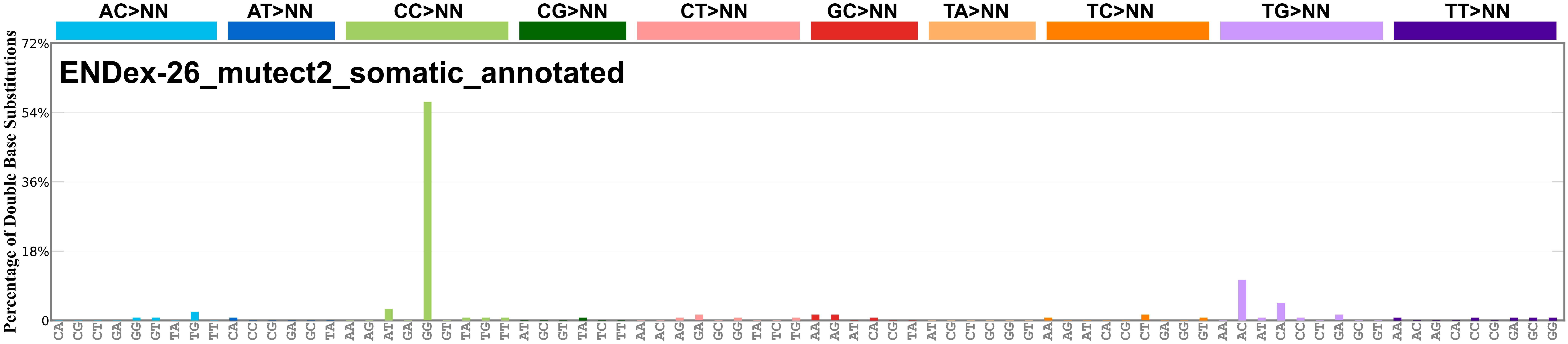

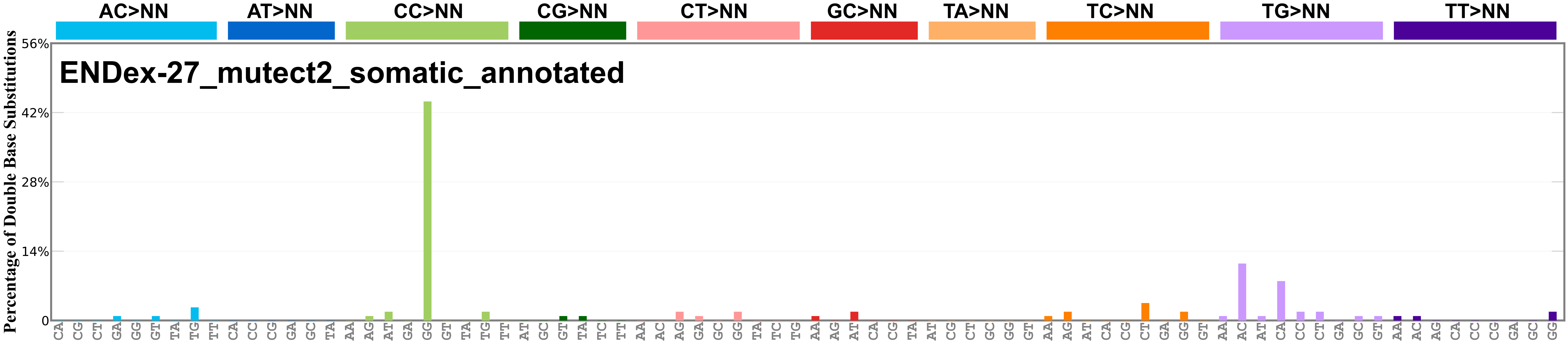

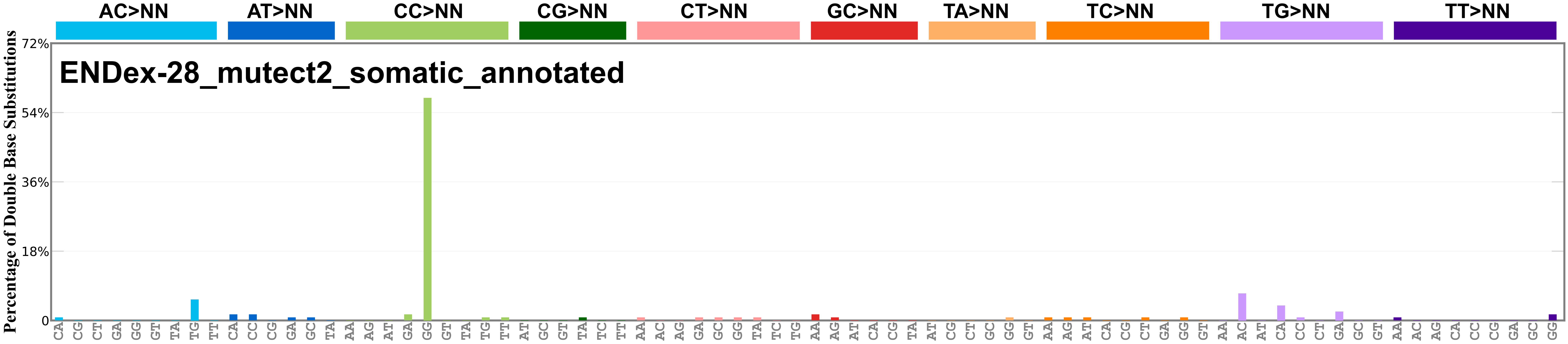

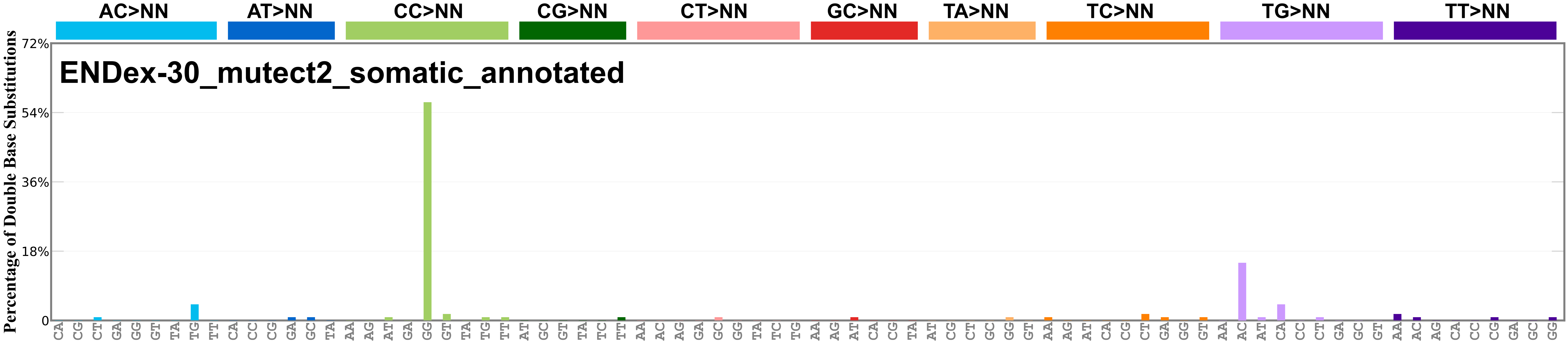

Supplement: Supplementary file 5 — Supplementary Material 5 [file 13402_2024_942_MOESM5_ESM.pdf]

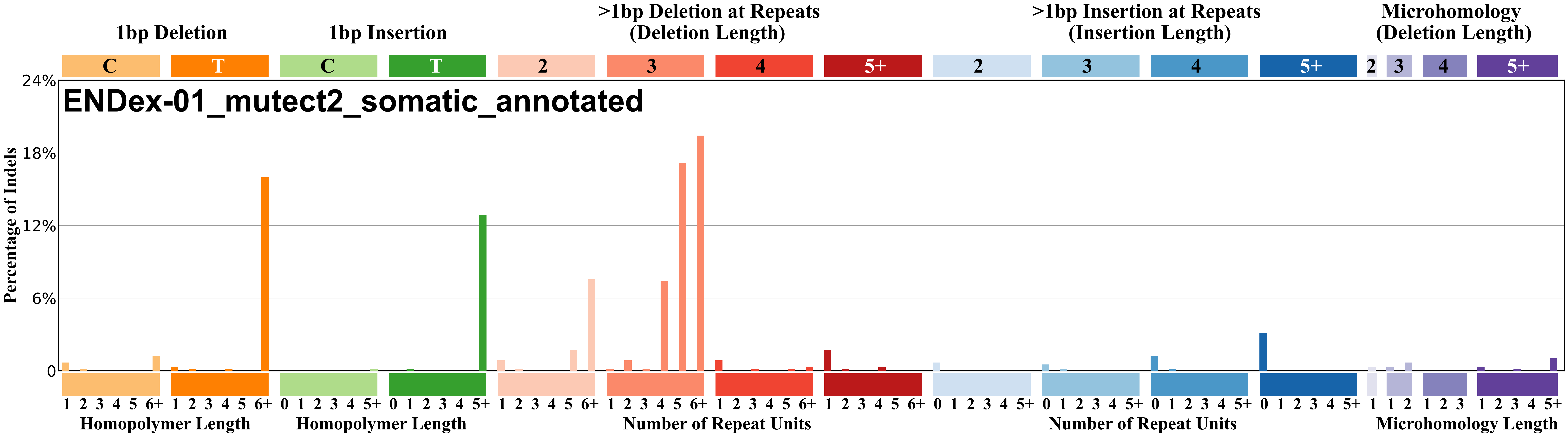

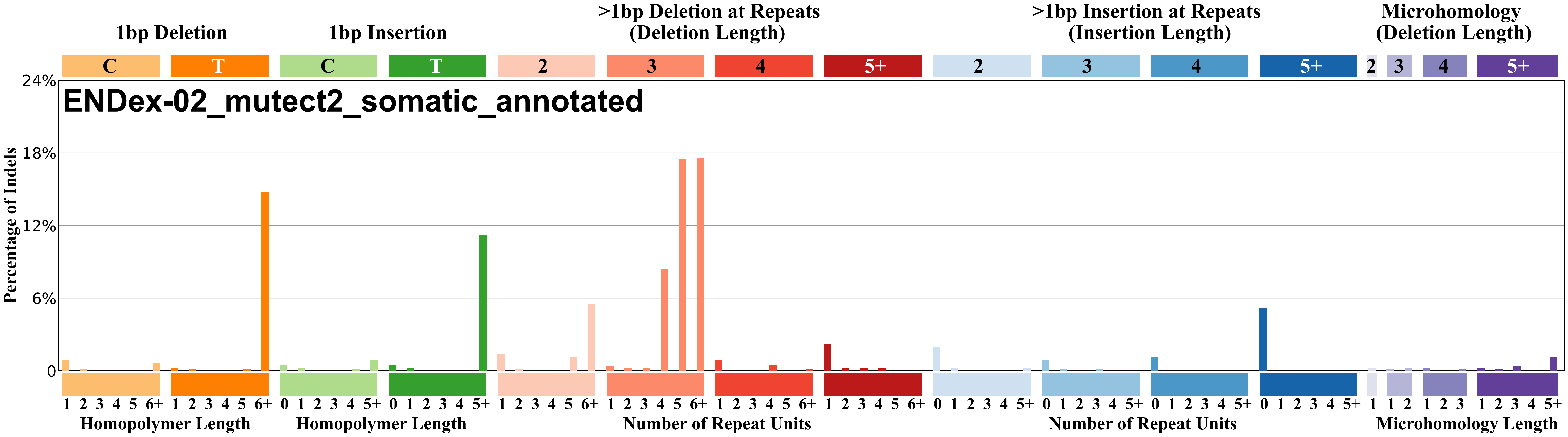

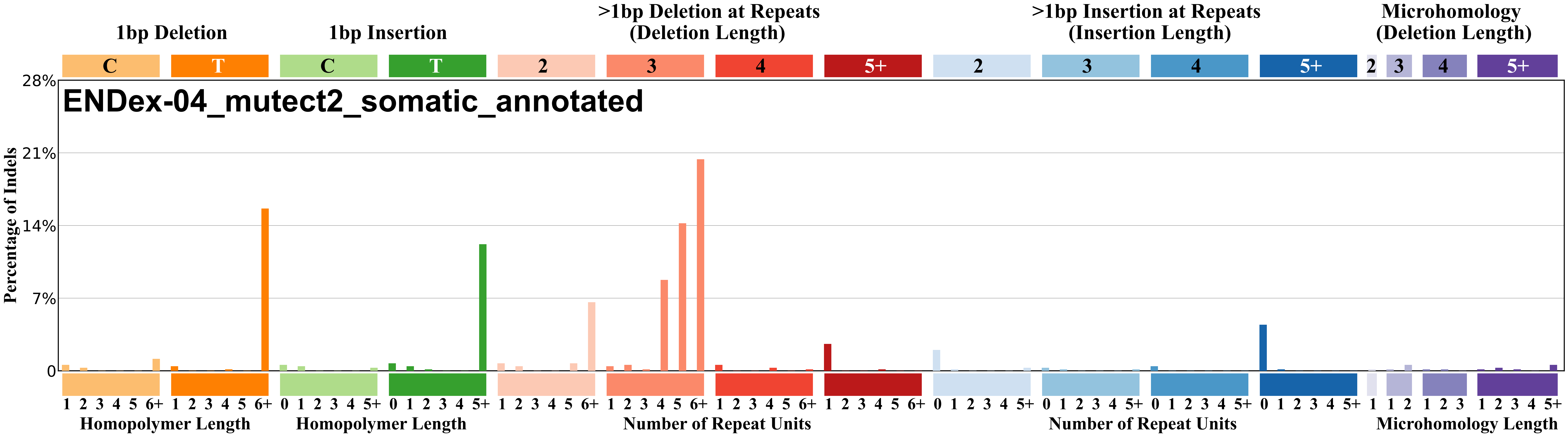

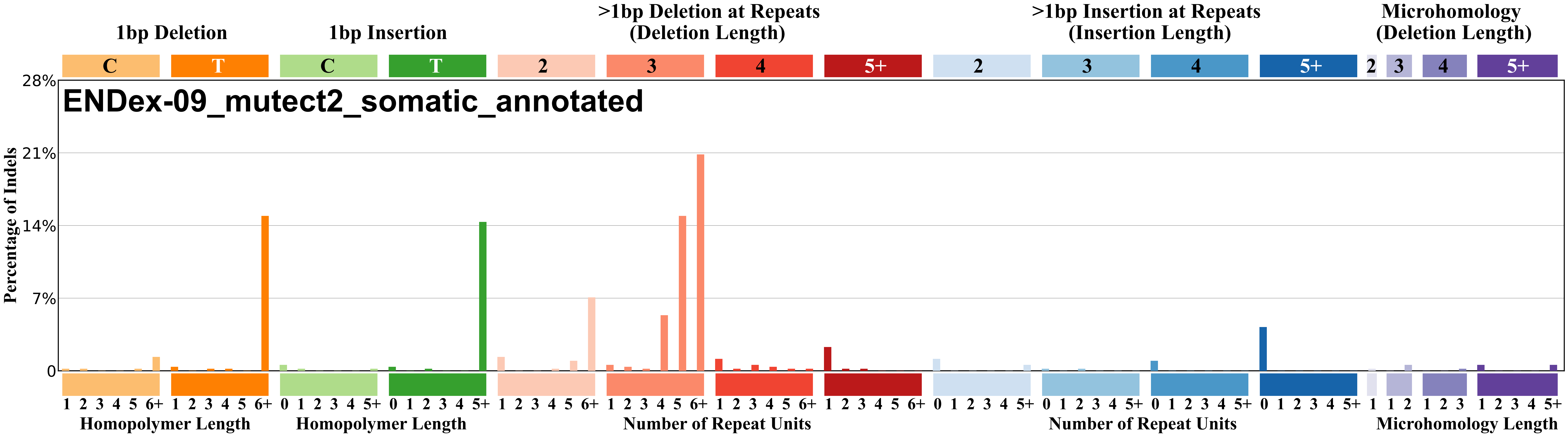

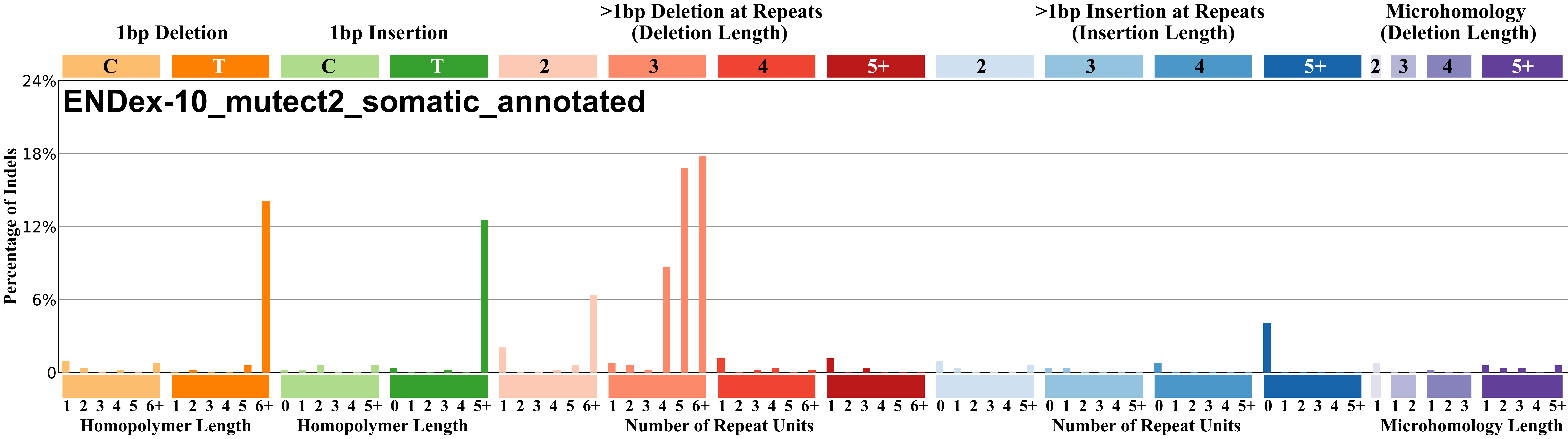

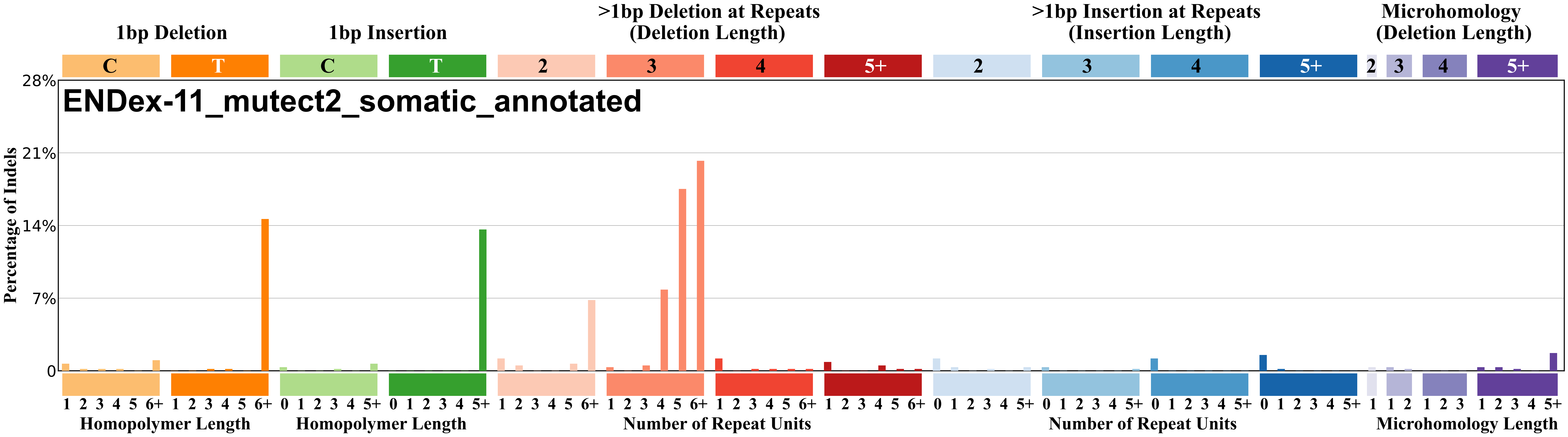

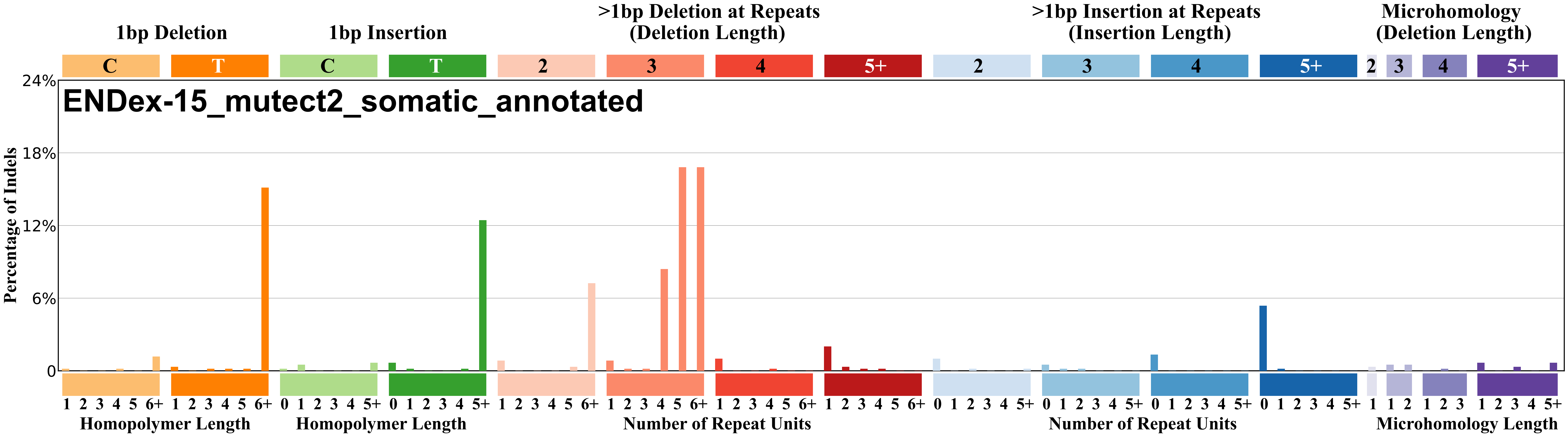

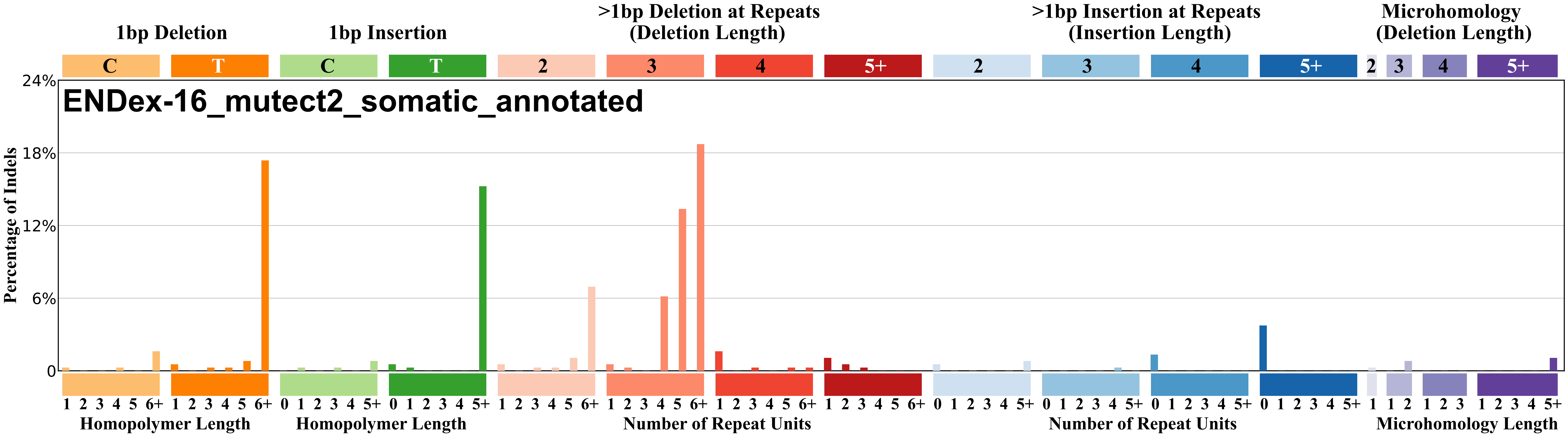

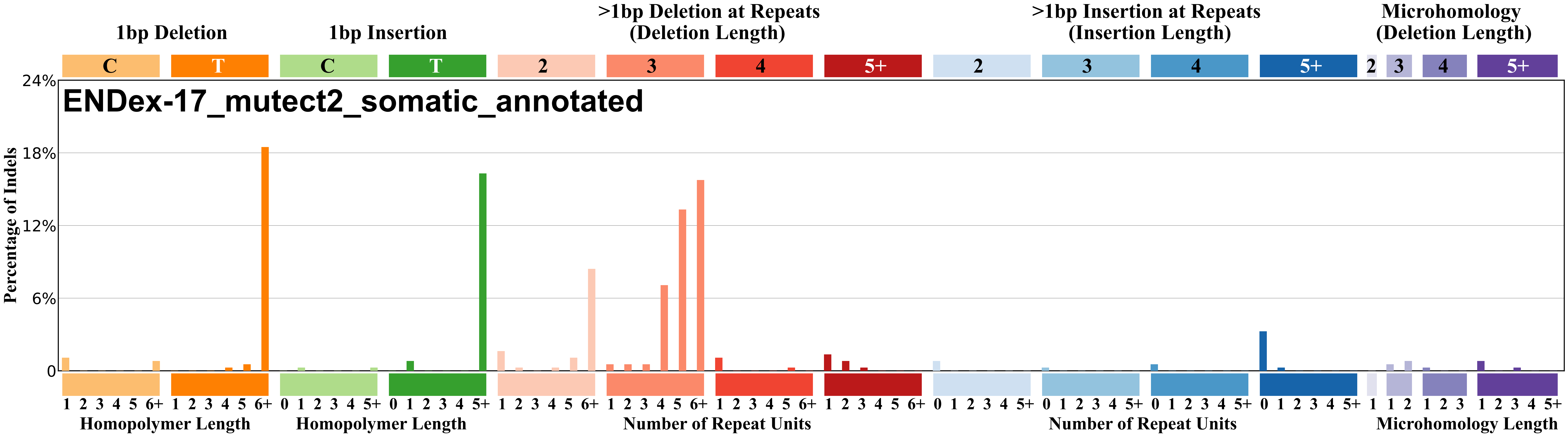

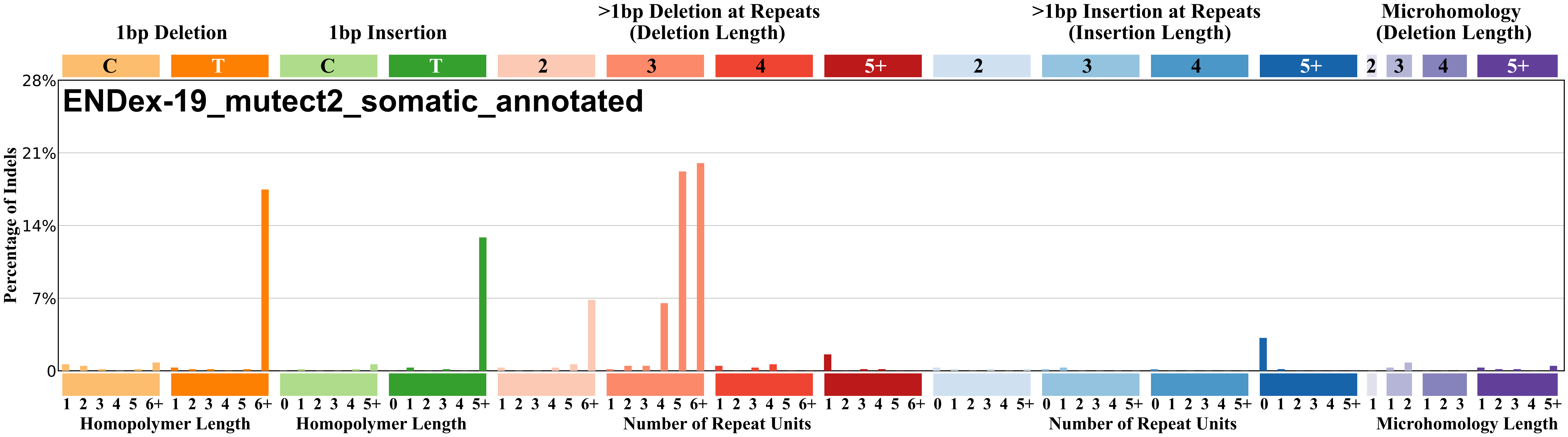

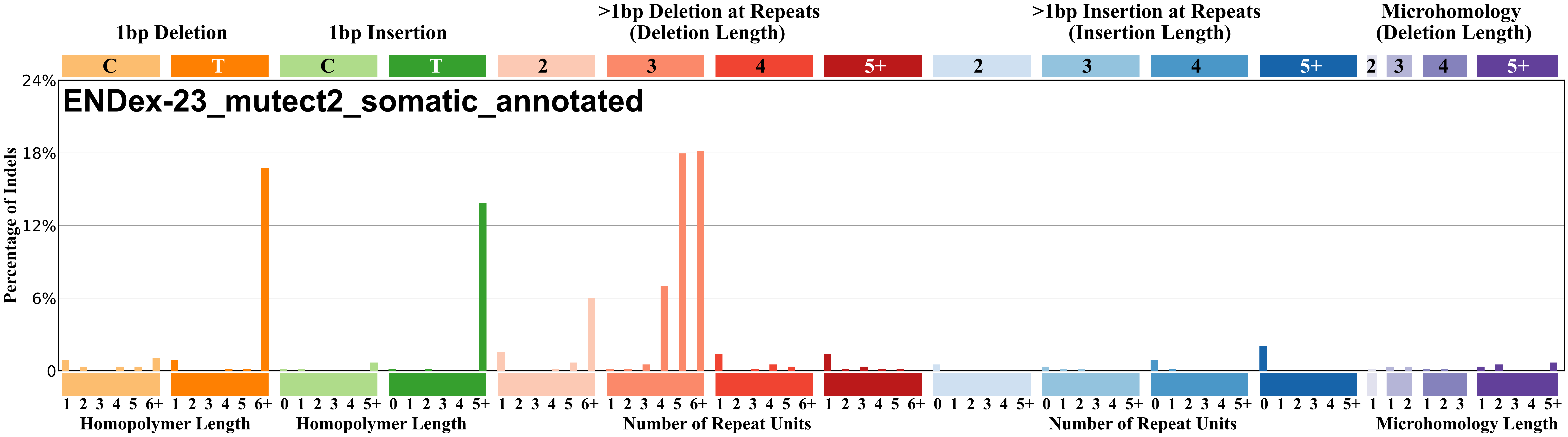

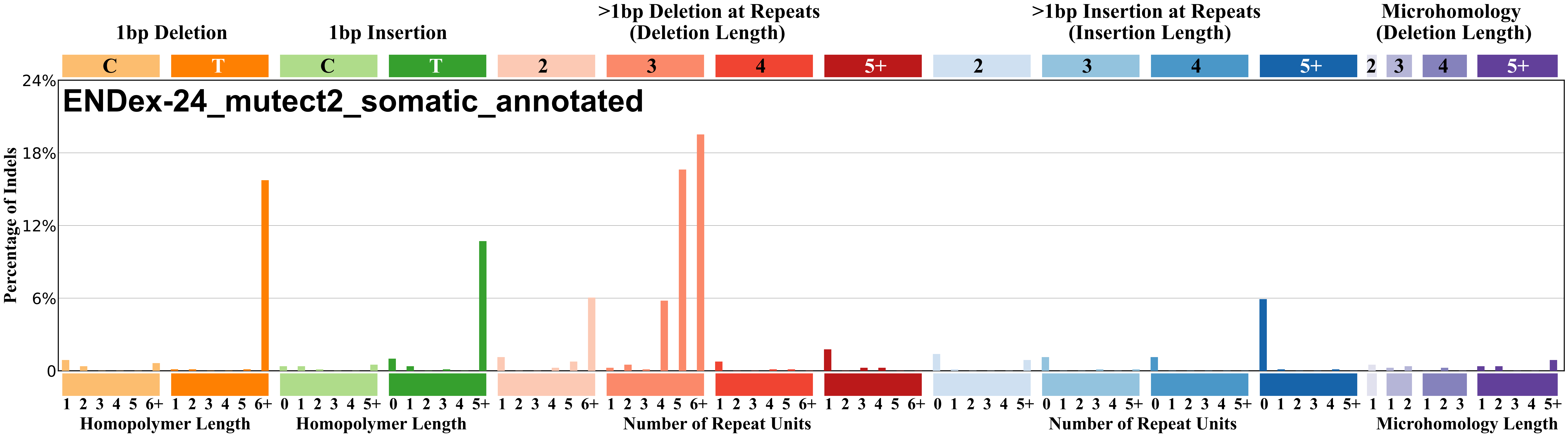

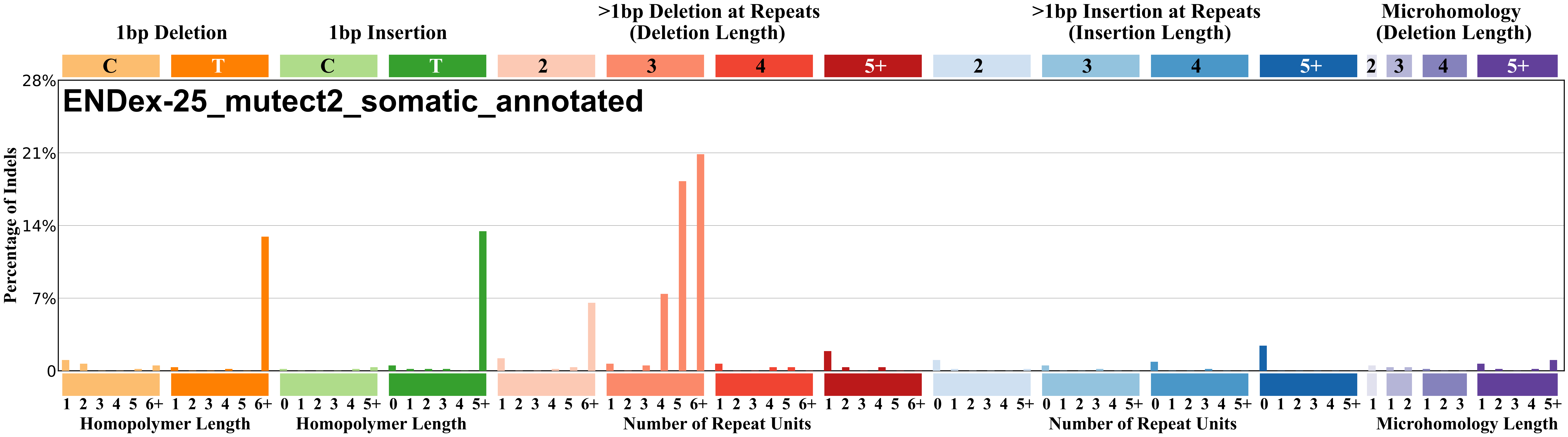

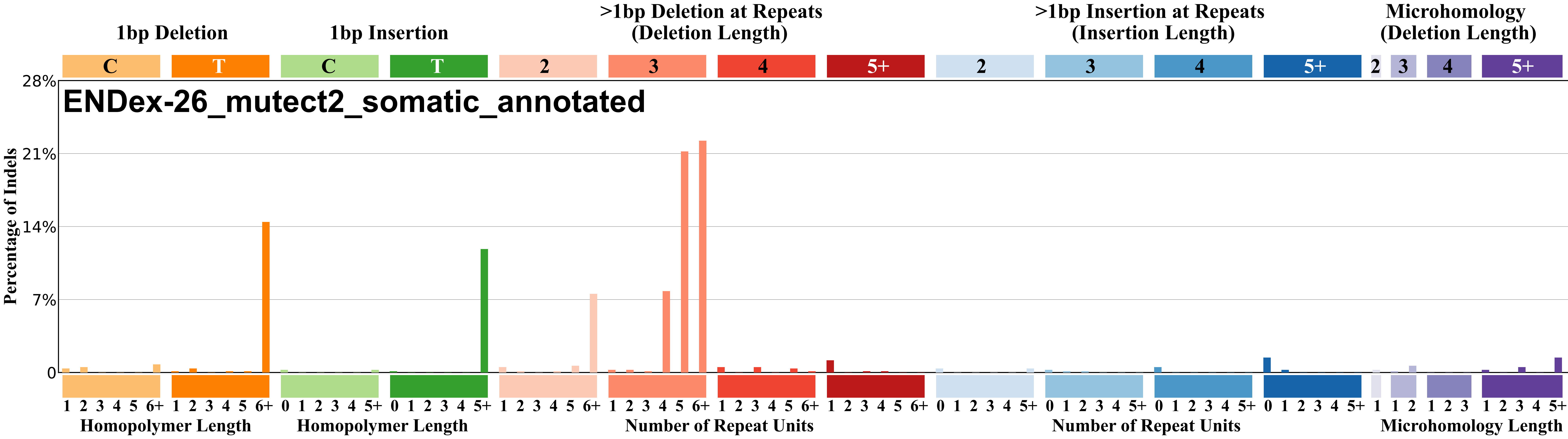

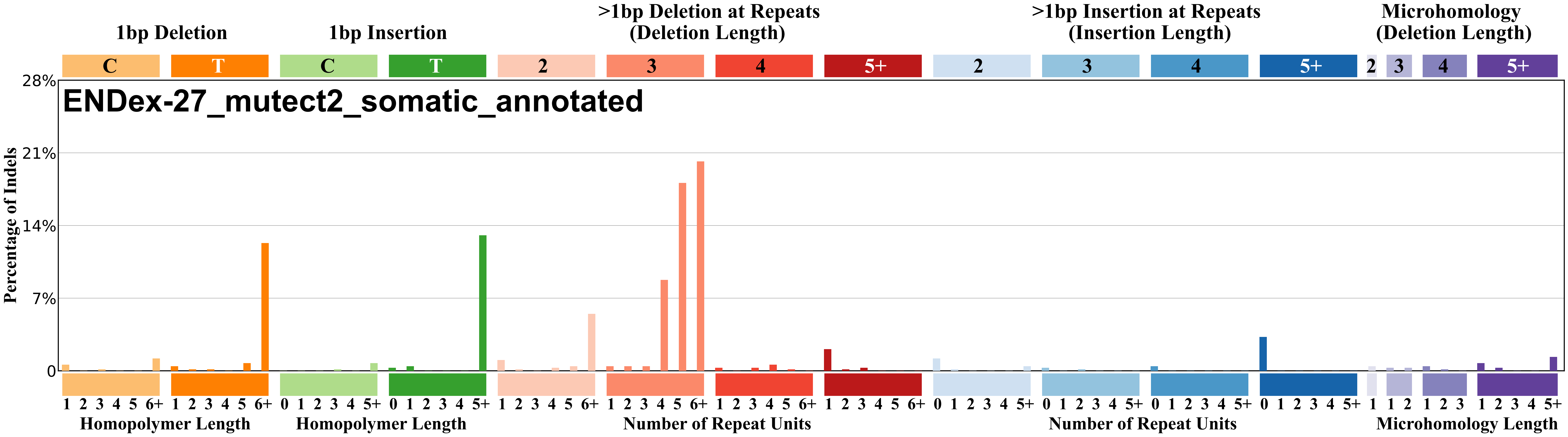

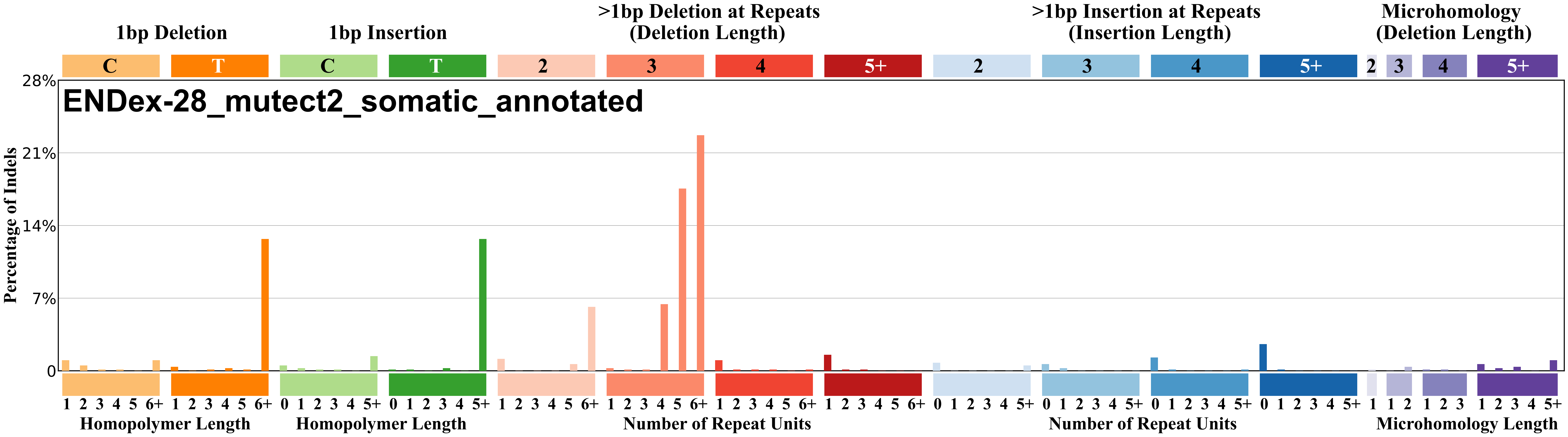

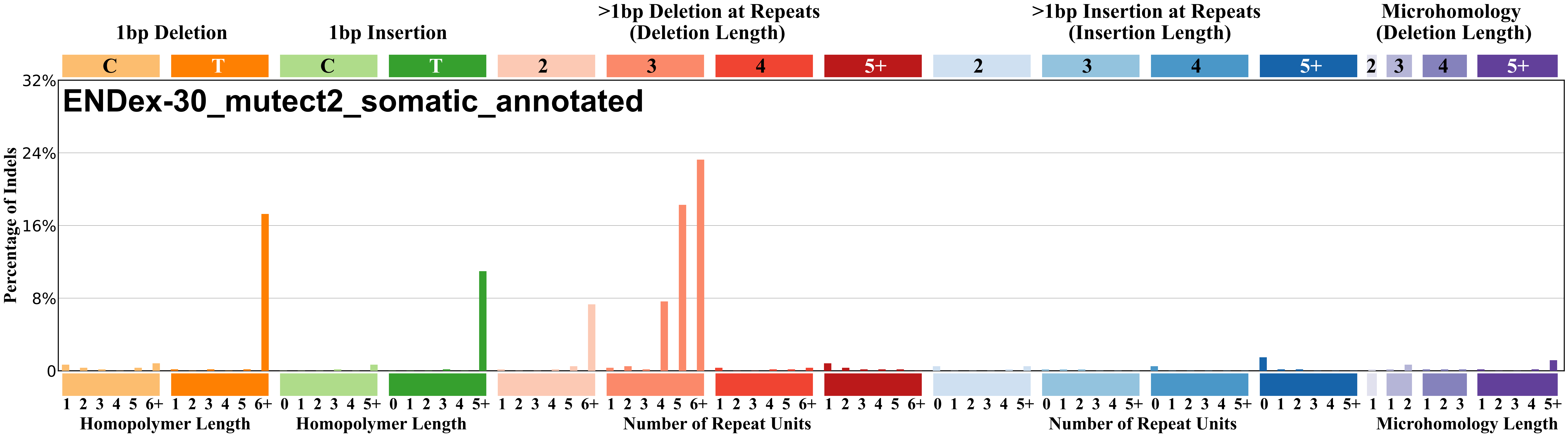

Supplement: Supplementary file 6 — Supplementary Material 6 [file 13402_2024_942_MOESM6_ESM.pdf]

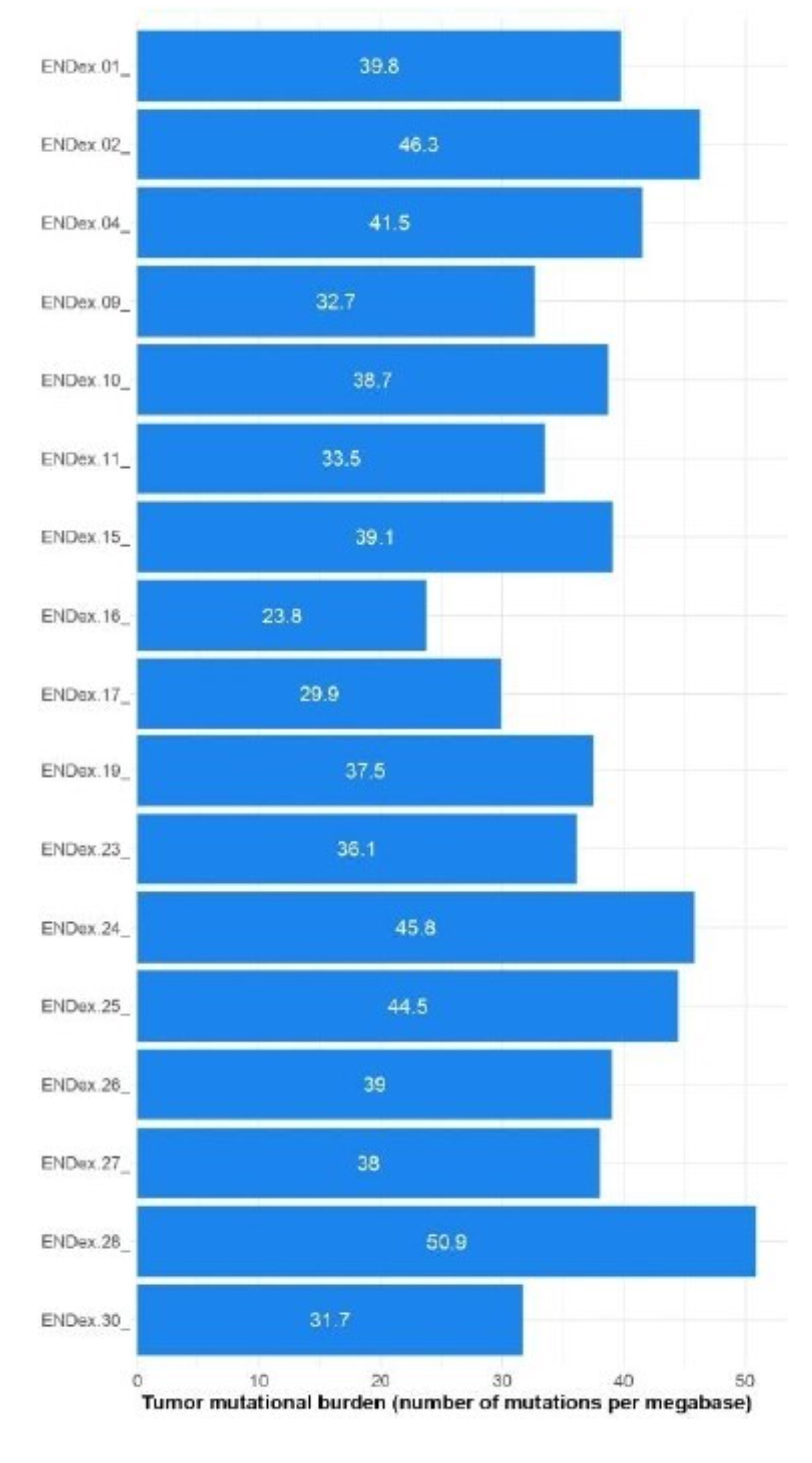

Supplement: Supplementary file 7 — Supplementary Material 7 [file 13402_2024_942_MOESM7_ESM.jpeg]
